# Light‐Driven Out‐of‐Equilibrium Helicity Control in a Diarylethene–Helicene Photosystem

**DOI:** 10.1002/anie.5252700

**Published:** 2026-08-19

**Authors:** Eric Sidler, Jinghao Wang, Christina Vallina, Ben L. Feringa

**Affiliations:** ^1^ Stratingh Institute for Chemistry University of Groningen Groningen the Netherlands; ^2^ Department of Chemistry University of Zurich Zürich Switzerland

**Keywords:** helicene, helicity, molecular switch, photoswitch

## Abstract

Molecular photoswitches are key tools to control responsive functions and to drive systems away from equilibrium by enabling kinetic asymmetry and energy ratchet mechanisms. Incorporating dynamic chirality control within such non‐equilibrium frameworks, however, remains challenging. Here, the responsive behavior of a diarylethene–[5]helicene photoswitch comprising multiple interconverting diastereomers is investigated. Distinct energy profiles govern the helicity dynamics in the open and closed forms of the switch. While helix inversion in the open state is kinetically hindered by a high energy barrier, the closed state allows rapid equilibration toward the thermodynamically favored diastereomer. Upon photochemical ring‐opening, the helicity acquired in the closed state is transferred and kinetically trapped in the open form, generating a persistent out‐of‐equilibrium configuration. This sequence constitutes a rare energy ratchet mechanism operating within a single covalently linked molecule, enabling directional control over helicity. These findings provide a concept for light‐driven helicity control in adaptive chiral systems.

## Graphical abstract

Distinct energy landscapes of different helicities in the open and closed states of a diarylethene–helicene photoswitch enable a light‐driven energy ratchet. Helix inversion occurs selectively in the closed form, and the resulting helicity is transferred and kinetically trapped upon ring opening, generating a persistent out‐of‐equilibrium chiral state and allowing directional helicity control.

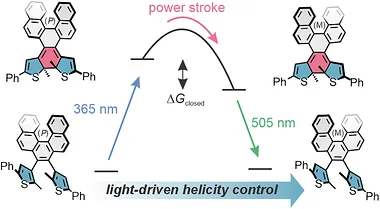

## 1 Introduction

Molecular switches are an important class of compounds that can reversibly alter their intrinsic properties by interconverting between two or more distinct states upon application of an external stimulus [1]. These changes can affect molecular geometry [2], absorption [3], luminescence [4], conductivity [5], supramolecular interactions [6], and magnetism [7], as well as other properties, enabling applications ranging from displays [8] and imaging [9] to sensing [10], computing [11], information storage [12], and photopharmacology [13]. Among these systems, molecular photoswitches form a particularly important subclass in which absorption of light induces excited‐state dynamics that lead to a new molecular state [14, 15]. Depending on the system, this state can either be thermodynamically stable, resulting in a bistable switch, or metastable and capable of reverting thermally or photochemically to the initial or another ground state. Access to such metastable states has been widely exploited in the design of light‐driven molecular motors, where photochemical excitation drives the system towards out‐of‐equilibrium states that enable directional motion through ratcheting mechanisms [16, 17, 18]. Beyond mechanical motion, light‐driven switching processes also provide opportunities to control stereochemical elements [19, 20, 21]. The ability to modulate chirality on demand is particularly attractive because it enables access to complementary chiral states without requiring reconfiguration of the entire system. For example, we have recently demonstrated that light‐driven population of a metastable state in a bis‐thioxanthylidene host enables efficient chiral induction and memory through a supramolecular deracemization process, where guest binding in the metastable state determines the helicity of the host after relaxation [22]. In a distinct approach, we showed that a helically extended molecular motor can control helicity through secondary helix inversion processes as the motor cycles through its four states [23]. These studies illustrate how photochemical switching can be harnessed to regulate stereochemical elements both inter‐ and intramolecularly. Diarylethenes represent particularly attractive photoswitching units due to their high robustness, low fatigue, and often quantitative switching behavior [24]. In addition, their efficient photochemistry allows access to kinetically trapped and out‐of‐equilibrium states, making them promising candidates for dynamic stereochemical control [25]. To use diarylethenes as stereochemical elements, an important requirement is the presence of configurationally stable atropisomers. In typical dithienylethenes (DTE) with five‐membered bridging rings, the atropisomers of the open form are configurationally labile and thus any chiral information stored in the closed form is lost upon ring‐opening (Figure 1a). Our group has demonstrated that the atropisomers of phenanthrene‐based diarylethene photoswitches interconvert slowly, enabling their isolation and thus the formation of the first diarylethene‐based chiroptical switch, where ring‐closure is associated with a helix inversion of the laterally formed helical units in the closed state (Figure 1b) [26]. We have also shown that the associated helix inversion upon ring closure of isolated atropisomers allows its translation at a supramolecular level in liquid crystals [27]. Extending the aromatic core from a phenanthrene to a [5]helicene unit can produce photochemically active systems that undergo efficient ring‐closure and ring‐opening reactions accompanied by changes in the aromatic character of the central helicene unit [28]. We envisioned that due to the presence of multiple stereochemical elements, such diarylethene–helicene hybrids can exist as several diastereomers and enantiomers in both the open and closed forms, which may interconvert through bond rotations, helix inversions, and photochemical transformations. Intrigued by this rich stereodynamic landscape, we investigated the thermal and photochemical interplay in such DTE‐helicene conformers, with particular emphasis on the control of helicity and access to out‐of‐equilibrium chiral states. Our study reveals that substantial energy differences between the diastereomers in the open and closed states enable efficient light‐driven helicity control through an energy ratchet mechanism [29]. As a result, the helicity of the [5]helicene moiety can be kinetically trapped by photochemical switching, providing access to an out‐of‐equilibrium helicity distribution as illustrated in Figure 1c.

**FIGURE 1:**
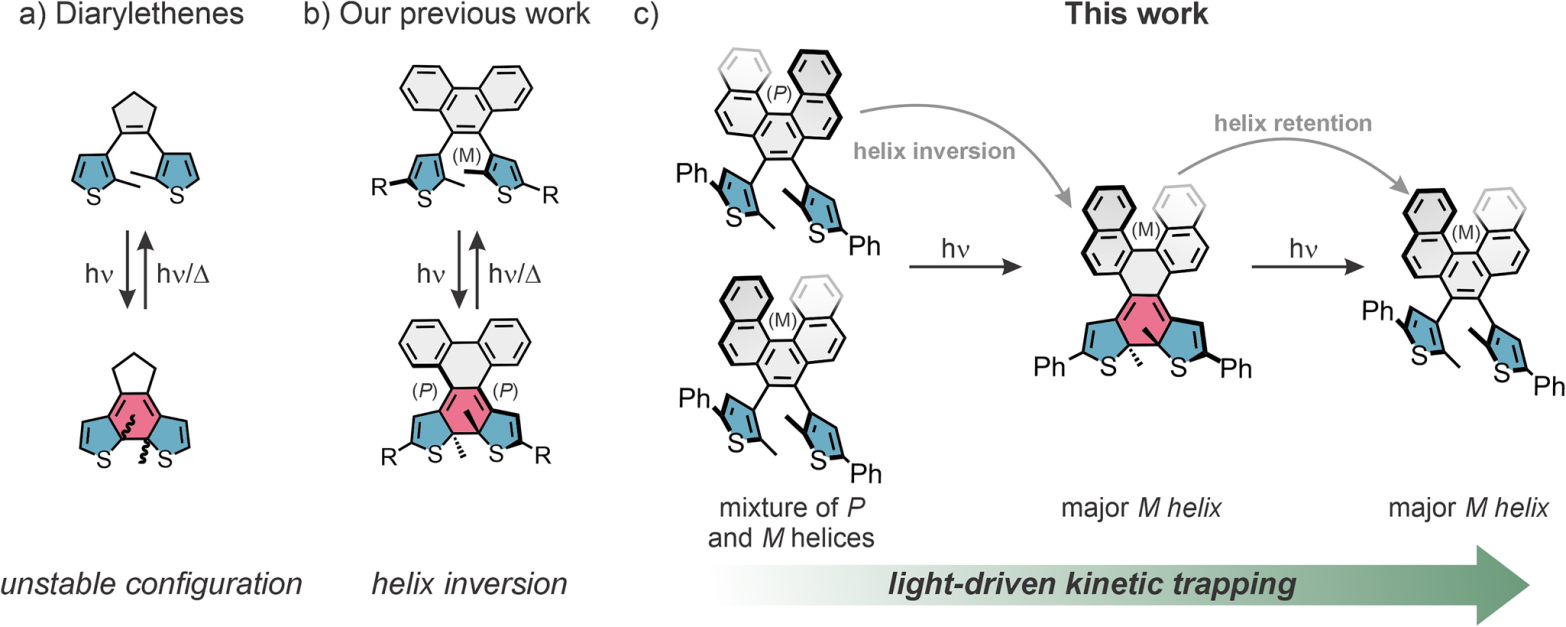
(a) Open and closed form of a DTE switch with a five‐membered bridging ring. (b) Our previously reported phenanthrene‐bridged DTE switch. Ring‐closure of enantiomerically pure open states results in helix inversion. (c) Overview of the herein presented light‐driven helicity control of the [5]helicene moiety through kinetic trapping.

## 2 Results and Discussion

Our design combines a DTE photoswitch, known for its reversible and efficient photochemical interconversion and rich stereochemistry, with a pentahelicene unit capable of undergoing helix inversion. This integration enables coupling between photoinduced switching and dynamic chirality, providing a platform to modulate helicity through external stimuli. The synthesis of the helicene diarylethene switch **1** is presented in the Supporting Information and was carried out following a recent procedure [28]. The different conformers of **1**, which form diastereomeric pairs of enantiomers, are displayed in Figure 2. For reasons of simplicity, the experimental results herein are discussed only for the lower row of enantiomers of Figure 2. Similarly, only the [5]‐helicene helicity descriptor (*M*/*P*) of the lower row is used for shorthand notation of each diastereomeric pair of enantiomers.

**FIGURE 2:**
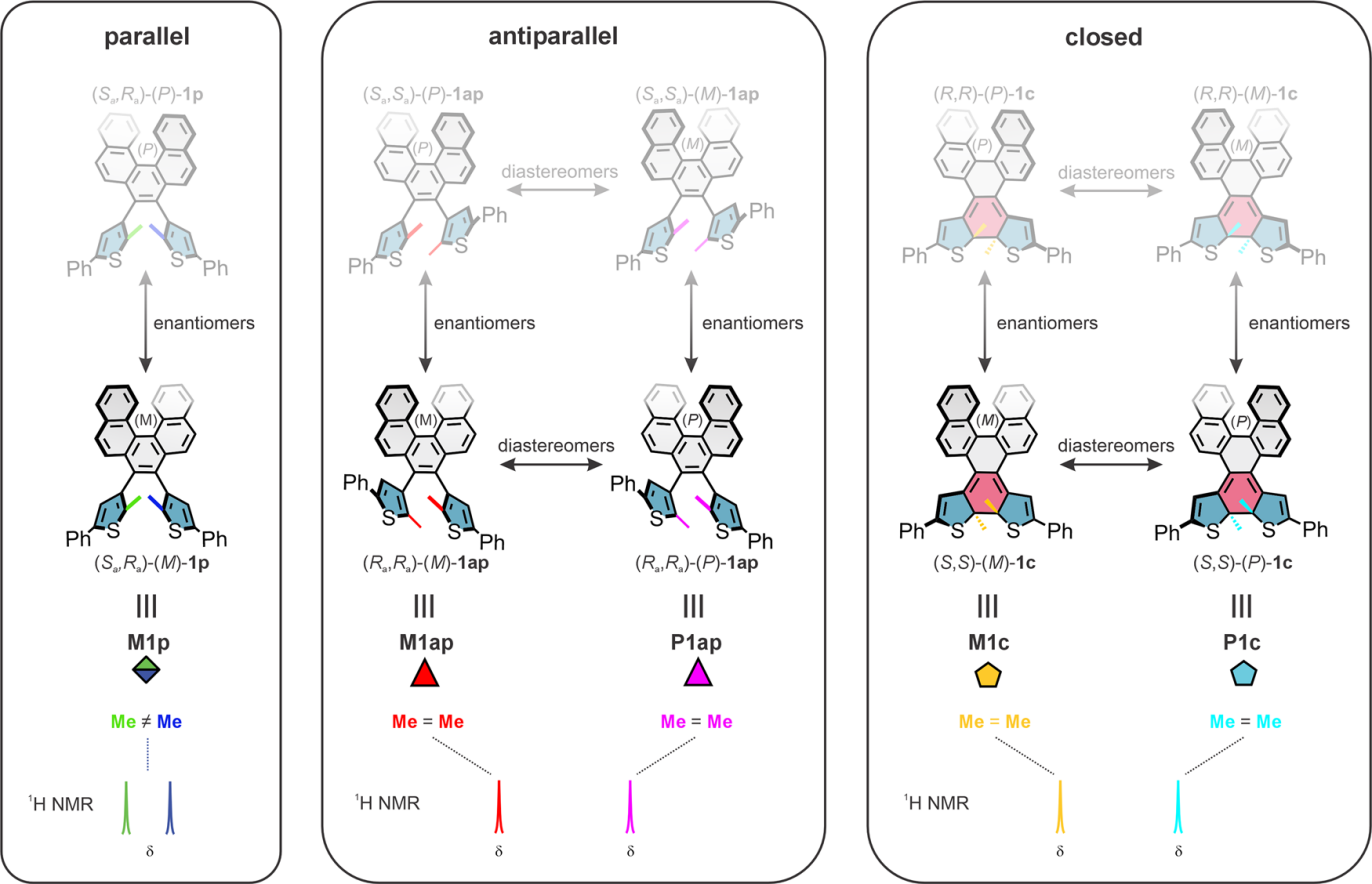
Overview of the different diastereomeric pairs of enantiomers of the parallel (left), antiparallel (middle), and closed (right) conformations. The ^1^H NMR signals of the methyl groups are qualitatively showcased at the bottom to highlight the different symmetries of the conformers. For reasons of simplicity, the shorthand denotations (**M1p**, **M1ap**, **P1ap**, **M1c**, and **P1c**) only represent the helicity descriptor of the compounds of the lower row of enantiomers. However, the experiments were performed on racemic samples, unless otherwise stated. Each group of enantiomers is also depicted with colored symbols in ^1^H NMR spectra discussed herein.

In the open state, the thienyl substituents can be arranged either as parallel or antiparallel atropisomers, wherein the former is typically known as the photochemically‐inactive and the latter as the photochemically‐active conformer [24, 26]. In the parallel conformer, the only chirality arises from the central [5]‐helicene unit due to the mirror plane between the thienyl substituents, resulting in a pair of enantiomers (*S*_a_,*R*_a_)‐(*P*)‐**1p**/(*S*_a_,*R*_a_)‐(*M*)‐**1p** (henceforth collectively labeled as **M1p**; Figure 2, left). In the antiparallel conformation, two different atropisomers of the thienyl substituents can be adopted ((*S*_a_,*S*_a_) and (*R*_a_,*R*_a_)) along with the chirality of the 5‐helicene, which leads to two diastereomeric pairs of enantiomers (*S*_a_,*S*_a_)‐(*P*)‐**1ap**/(*R*_a_,*R*_a_)‐(*M*)‐**1ap** and (*S*_a_,*S*_a_)‐(*M*)‐**1ap**/(*R*_a_,*R*_a_)‐(*P*)‐**1ap** (henceforth labeled as **M1ap** and **P1ap**, respectively; Figure 2, middle). Structurally, the methyl groups of **M1ap** point to opposite sides relative to the helicene moieties, whereas the methyl groups in **P1ap** point to the same side relative to the helicene units. In the closed state, the axial chirality of the thienyl moieties is transformed into a fixed point chirality, leading to two diastereomeric pairs of enantiomers (*R*,*R*)‐(*P*)‐**1c**/(*S*,*S*)‐(*M*)‐**1c** and (*R*,*R*)‐(*M*)‐**1c**/(*S*,*S*)‐(*P*)‐**1c** (henceforth labeled as **M1c** and **P1c**, respectively; Figure 2, right). Like in the antiparallel conformers, the methyl groups of **M1c** point towards opposing sides and the methyl groups of **P1c** point towards the same side relative to the helicene halves.

Figure 3a displays the methyl region of the ^1^H NMR spectrum of a thermally relaxed mixture of **1** in the open state. Conveniently, the different conformers of the open and closed states can be distinguished in the ^1^H NMR spectra by analysis of their methyl resonance signals. The methyl substituents on the thiophene rings in the parallel conformation are in a chemically and magnetically inequivalent position, resulting in two separate signals in the ^1^H NMR spectrum for the methyl substituents. Integration of the methyl peaks thus allows direct assignment of the two signals present in a 1:1 ratio to the parallel isomer **M1p**. In contrast, the high symmetry of the antiparallel conformation renders the methyl substituents both chemically and magnetically equivalent, leading to a single resonance for both methyl groups. However, due to the presence of the helical unit, the antiparallel framework exists as two diastereomeric forms, **M1ap** and **P1ap**, which give rise to two distinct methyl signals in the ^1^H NMR spectrum. The ratio of these two separate methyl resonance signals is not necessarily 1:1 but rather reflects the energy difference of **M1ap** and **P1ap**. This becomes eminent by the two remaining methyl signals in Figure 3a (see also Figure S2), which have a ratio of 43:57 and thus indicate a slight energy difference of the two diastereomers. Boltzmann‐weighted single‐point energies computed at the ωB97X‐D4/def2‐TZVPP/CPCM(toluene) [30, 31, 32] level of theory, based on geometries optimized at the r^2^SCAN‐3c/CPCM(toluene) [33] level, indicate that **P1ap** is slightly more stable than **M1ap**, with Δ*G*_o_ = 2 kJ/mol (see Figures 4b and S39; computational details are provided in the Supporting Information). The more downfield‐shifted signal with a lower integral was therefore assigned to **M1ap**, while the upfield‐shifted signal was attributed to **P1ap**. This assignment is also confirmed by the structural features of the two conformers: In **P1ap**, the methyl groups are oriented toward the helicene units and thus experience the anisotropic shielding effects of the helicene ring currents, resulting in an upfield shift compared to **M1ap**. Conversely, in **M1ap** the thiophene protons are positioned closer to the helicene framework, which accounts for their relative upfield shift compared to **P1ap** (Figures S5 and S6). This assignment is further supported by DFT calculations using the GIAO method at the ωB97X‐D4/def2‐TZVPP/CPCM(toluene) level of theory, which predict larger Boltzmann‐weighted isotropic shielding values for the methyl protons of **P1ap** than for those of **M1ap**, consistent with the experimentally observed upfield shift. Conversely, the thiophene proton is predicted to exhibit a slightly larger Boltzmann‐weighted isotropic shielding in **M1ap** than in **P1ap**, in agreement with its relative upfield shift (see Table S3). Similarly to the antiparallel conformers, the methyl substituents in the closed state are chemically and magnetically equivalent and thus appear as a single singlet, one for each diastereomeric pair of enantiomers. Interestingly, for the closed state, DFT calculations predict **M1c** to be significantly more stable than **P1c**, with a Δ*G*_c_ = 39 kJ/mol (see also Figures 4b and S39; computational details are provided in the Supporting Information), which already indicates potential helicity control in the closed state versus the open state (*vide infra*).

**FIGURE 3:**
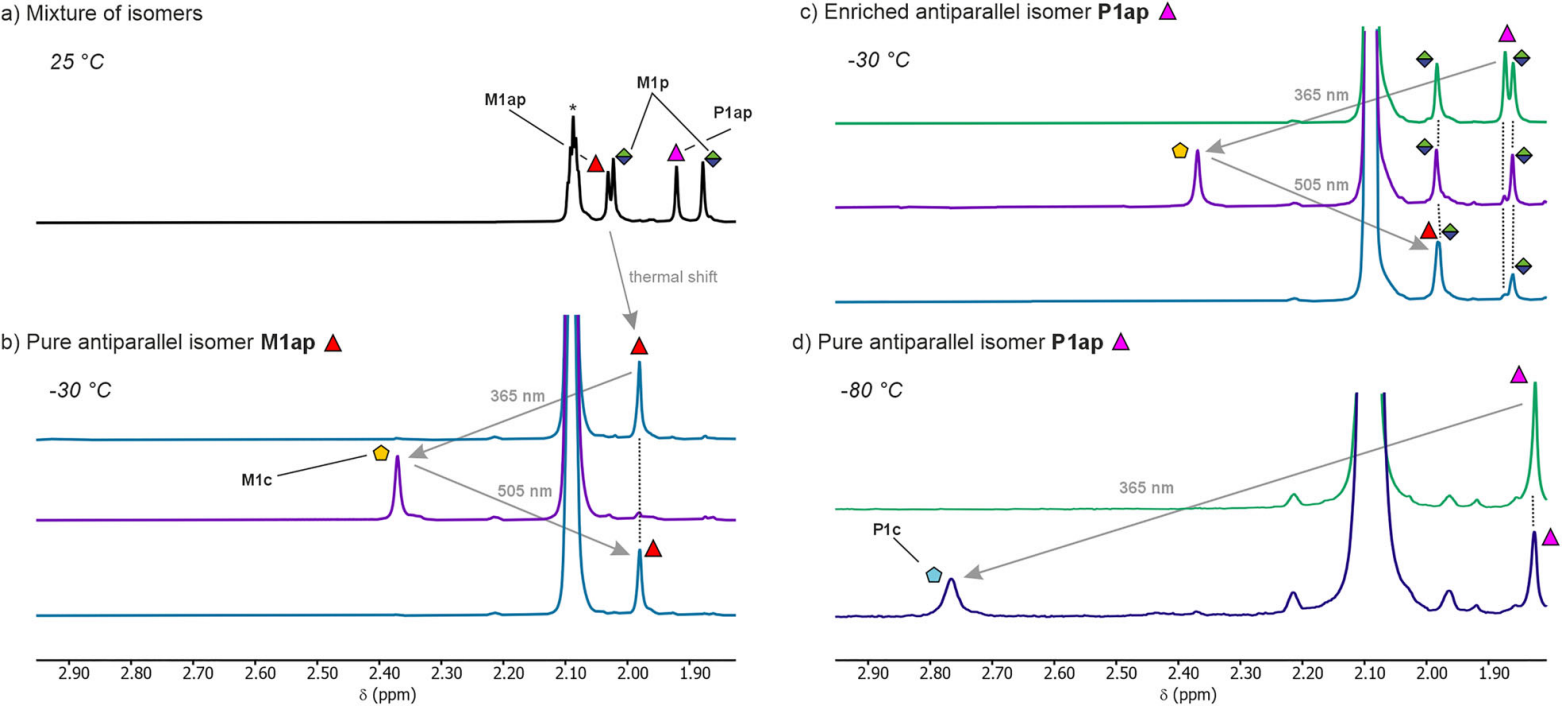
Methyl region of the ^1^H NMR (500 MHz, toluene‐*d*_8_) spectra of different mixtures of isomers and their respective changes upon light irradiation are displayed. (a) Thermally relaxed mixture of switch **1** at 25°C. (b) Pure antiparallel isomer **M1ap** at −30°C and its changes upon irradiation with 365, followed by 505 nm. (c) Enriched antiparallel isomer **P1ap** at −30°C and its changes upon irradiation with 365, followed by 505 nm. (d) Pure antiparallel isomer **MP1ap** at −80°C and its changes upon irradiation with 365 nm.

**FIGURE 4:**
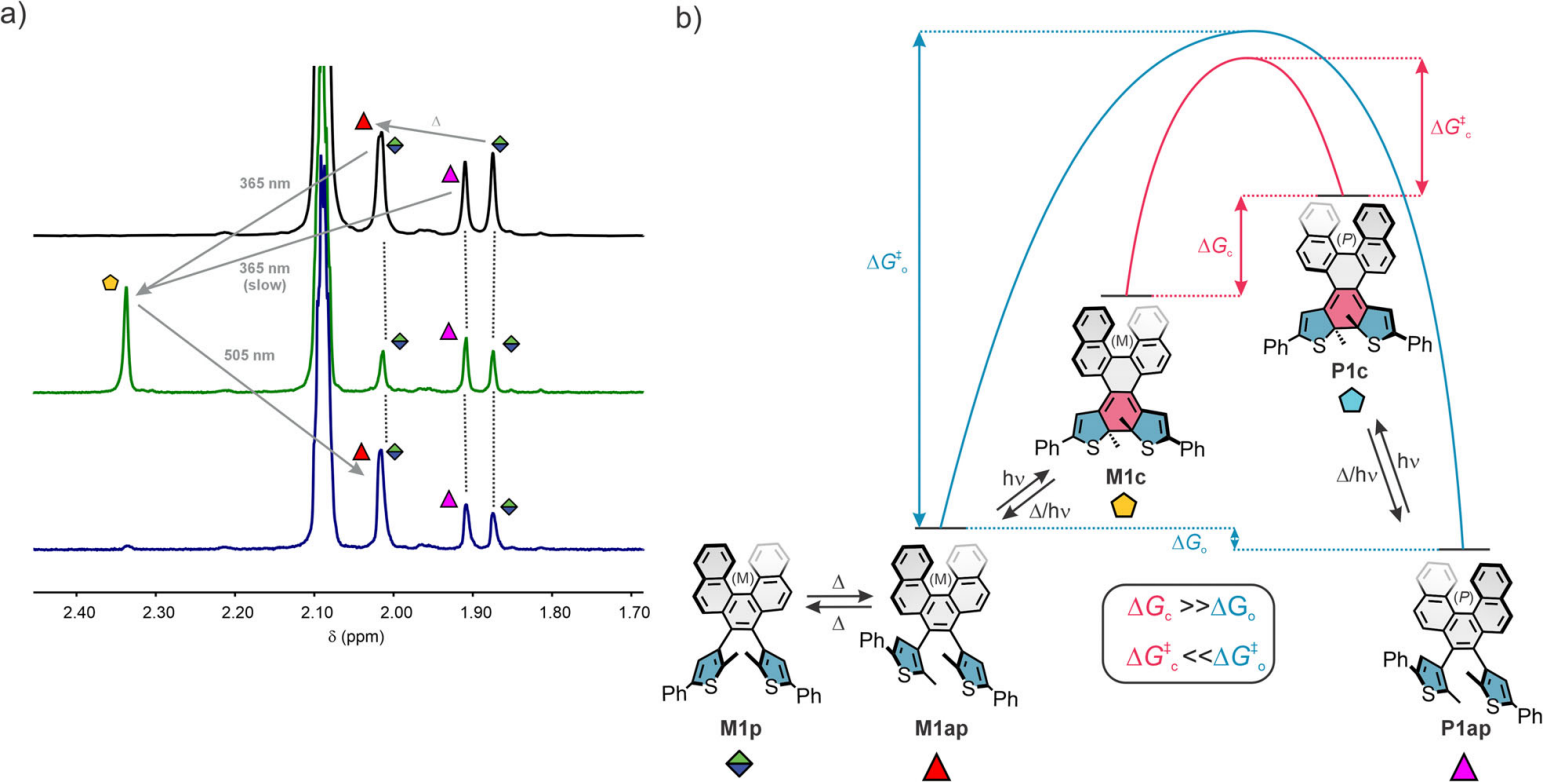
(a) Methyl region of the ^1^H NMR (500 MHz, toluene‐*d*_8_) spectrum of a thermally relaxed mixture of switch 1 and its changes upon irradiation with 365 nm and 505 nm at 0°C. (b) Diagram displaying the various thermal and photochemical interconversions between the different conformers, as well as the energy requirements for the kinetic trapping.

Figure 3b displays the methyl region of the ^1^H NMR spectrum of **M1ap**, which was isolated via HPLC (see Supporting Information for details). When the sample was irradiated with 365 nm at −30°C, a new downfield‐shifted signal corresponding to the methyl substituents of the closed species **M1c** appeared with a near‐quantitative photostationary state (PSS). Ring‐opening induced by irradiation with 505 nm resulted in the reformation of the initial diastereomer **M1ap** without any detectable side‐products (see also Figures S22–S25). The ring‐closure of **M1ap** is also associated with significant changes in the absorption spectrum, consistent with the literature report (Figure S10) [28]. The quantum yield of photoswitching was determined to be Φ**_M1ap_**_→_ **_M1c_** = 0.43 (see Supporting Information for details). Furthermore, thermal ring‐opening at 20°C allowed the determination of its energy barrier of Δ*G* ^‡^ **_M1c_**_→_ **_M1ap_** = 88 kJ/mol by following the absorption time traces (Figures S11 and S12). Photoswitching also leads to considerable changes in the circular dichroism (CD) spectra of enantiopure samples of **M1ap** (Figure S16).

Interestingly, when irradiating a mixture enriched with **P1ap** (and with **M1ap** absent) with 365 nm at −30°C in the NMR, the same closed species **M1c** is formed (Figure 3c). Notably, however, the ring‐closing reaction of **P1ap** to **M1c** was significantly slower than for **M1ap** (*vide infra*). Ring‐opening with 505 nm subsequently led to the formation of the **M1ap** isomer that was initially absent in the reaction mixture, while the photochemically inactive parallel isomer **M1p** remained at a relatively constant concentration (Figures 3c and S26–S29). Mechanistically, this implies that a helix inversion of the [5]‐helicene unit occurs in the closed state, whereby the initially formed diastereomer **P1c** is only transiently populated and thermally relaxes to the more stable **M1c** isomer via helix inversion. Essentially, **P1ap** is thus funneled into the nearly isoenergetic **M1ap** conformer through ring closure to **P1c**, followed by helix inversion in the light‐induced closed state to **M1c**, and subsequent ring opening at longer wavelengths to generate **M1ap** (Figure 4b). As mentioned above, there is a significant energy difference between the diastereomers in the closed state of Δ*G*_c_ = 39 kJ/mol, consistent with the experimental observation and thus explaining the thermal relaxation of **P1c** to **M1c**. The higher energy of **P1c** relative to **M1c** also explains why photochemical switching appears much slower. Because **P1c** is destabilized, its thermal ring‐opening back to **P1ap** is greatly accelerated compared to the ring‐opening of **M1c**. This was confirmed by low‐temperature UV–vis experiments at −80°C that allowed the determination of the thermal ring‐opening barrier of Δ*G* ^‡^ **_P1c_**_→_ **_P1ap_** = 54 kJ/mol, which is considerably smaller than for **M1c** (Figures S14 and S15). As a consequence, **P1c** reopens rapidly and cannot accumulate as quickly at −30°C in the NMR experiment. Only upon relaxation to **M1c** is **P1c** essentially removed from the photochemical equilibrium between **P1ap** and **P1c**, thus driving the ring‐closure and consumption of **P1ap**. To confirm the rapid relaxation of **P1c** to **M1c** at −30°C through a helix inversion, the transition state geometry was optimized through DFT calculations at the r^2^SCAN‐3c/CPCM(toluene) level of theory and its energy subsequently refined using single‐point calculations at the ωB97X‐D4/def2‐TZVPP/CPCM(toluene) level, revealing a low inversion barrier of Δ*G*_c_^‡^ = 84 kJ/mol when going from **P1c** to **M1c** (Figures S38 and S39). This is considerably lower than the helix inversion in the open antiparallel conformation, which was calculated to be Δ*G*_o_^‡^ = 114 kJ/mol (see Supporting Information). This value is higher than the racemization barrier of parent [5]‐helicene (Δ*G*_rac_^‡^ = 100.8 kJ/mol) [34, 35]. To further confirm the existence and transient population of **P1c**, the irradiation experiment was repeated at −80°C (Figure 3d), which slows down both the thermal ring‐opening back to **P1ap** and thermal relaxation to **M1c** in the closed state. After irradiating a sample of **P1ap** with 365 nm at this temperature, a new, more downfield‐shifted signal is observed, corresponding to **P1c** with a PSS of 67:33 (**P1c**:**P1ap**) (see also Figures S36 and S37). The quantum yield of photoswitching for **P1ap** was determined to be Φ**_P1ap_**_→_ **_P1c_** = 0.60 (see Supporting Information for details).

Next, we examined the ring‐closing/ring‐opening photochemical cycle at 0°C starting from the thermally relaxed mixture of isomers **M1p**, **M1ap**, and **P1ap** (Figures 4a and S30–S33). As expected, the *M*‐configured open form (**M1ap**) was readily consumed upon irradiation and quickly reached a PSS with the closed isomer **M1c**. In contrast, ring‐closure of the *P*‐configured open form (**P1ap**) was extremely sluggish, and only slow conversion was detected. This pronounced difference in apparent reactivity can be explained by the much faster thermal ring‐opening of **P1c** (*vide supra*). Consequently, the equilibrium and therefore the **P1ap**/**P1c** photostationary ratio at 0°C is strongly biased toward **P1ap** (Figure 4b). In addition, once the PSS of **M1ap**/**M1c** is established, the high steady‐state concentration of **M1c** further suppresses effective funneling from **P1c** to **M1c** through the helix inversion. Any **P1c** formed must compete with this large reservoir of **M1c**, making net relaxation from **P1c** to **M1c** slow. As a result, thermal reopening of **P1c** back to **P1ap** dominates over helix inversion to **M1c** on the experimental timescale, causing **P1ap** to react very slowly under continuous irradiation. In the low‐temperature experiment at –30°C (Figure 3c), where no **M1ap** was present, consumption of **P1ap** proceeded efficiently, albeit slowly, because any **P1c** formed could relax to **M1c** without being suppressed by an already established **M1ap**/**M1c** PSS. In other words, the absence of **M1c** accumulation via **M1ap** cyclization eliminates the competitive equilibrium that normally disfavors stereochemical relaxation of **P1c**, allowing the *P*→*M* funneling pathway to operate effectively in the closed state. Interestingly, at 0°C, slow conversion of the parallel isomer **M1p** into **M1ap** was also observed. This can be deduced from the time‐dependent integration curve, which shows slow consumption of parallel isomer **M1p** and additional formation of **M1c** after its initial quick build‐up through the ring‐closing step of **M1ap** (Figure S31). Mechanistically, the consumption of **M1p** occurs via a thermal interconversion to **M1ap [** 26, 36] followed by the light‐driven ring‐closure to **M1c**. Upon ring‐opening with 505 nm, all formed **M1c** is quickly consumed to get the ring‐opened product **M1ap**. The remarkable observation is that overall, a thermally equilibrated mixture of all isomers (Figure 4a, top) is essentially funneled to an out‐of‐equilibrium state enriched in **M1ap** (Figure 4a, bottom) through a helix inversion in the closed state and thermal interconversions of the parallel conformers in the open state. The amount of funneled compound could be even increased by repeating the same experiment at 45°C, where the rate of interconversion of the parallel → antiparallel conformers was considerably increased (Figures S34 and S35). However, at this temperature, slight decomposition was also observed, which led to an overall decrease in product. The diagram in Figure 4b summarizes the calculated energy values of the different conformers and visualizes the various interconversions between the different states. In the open state, there is only a minor energy difference between **M1ap** and **P1ap** (Δ*G*_o_ = 2 kJ/mol) and their interconversion through a helix‐inversion has a relatively high barrier (Δ*G*_o_^‡^ = 114 kJ/mol). In the closed state, however, there is a significant energy difference between **M1c** and **P1c** (Δ*G*_c_ = 39 kJ/mol) accompanied by a considerably lower barrier for the helix‐inversion (Δ*G*_c_^‡^ = 84 kJ/mol). These ground‐state and transition‐state energy differences essentially allow to kinetically trap the *M* helicity in the open state through a helix inversion in the closed state. The overall switching cycle thus fundamentally constitutes a molecular energy ratchet mechanism that couples photochemical and thermal processes to achieve directional helicity control [29, 37]. Irradiation breaks balance and drives the system out of equilibrium. In the closed state, the lowered helix‐inversion barrier allows relaxation toward the thermodynamically preferred diastereomer, whereas subsequent fast ring opening kinetically traps this helicity in the open state. In this way, helix inversion in the closed form acts as a directional bias (power‐stroke‐like step), while photochemical switching provides the energy input required for ratcheting.

## 3 Conclusion

Combining a DTE switch with a [5]helicene gives rise to a dynamic chiral structure with multiple diastereomeric pairs of enantiomers that are interconnected through thermal and photochemical pathways, enabling an intricate interplay between conformational chiral states. In the open form, the two helicities of the helicene unit are nearly isoenergetic, and their interconversion is slow due to a high helix‐inversion barrier. In contrast, in the closed form, one helicity is strongly favored, and the inversion barrier is significantly reduced, allowing rapid equilibration. Consequently, irradiation of an open‐state mixture of helicities leads to helix inversion in the closed state toward the thermodynamically preferred diastereomer. Subsequent ring opening at longer wavelengths occurs fast and without compromising the helicity that was maintained in the closed state, thereby kinetically trapping the induced helicity and transferring helicity control from the closed to the open form. Essentially, helicity is controlled through a molecular energy ratchet mechanism, in which light acts as a balance‐breaking energy input and helix inversion in the closed state provides the directional bias (power‐stroke‐like step). This example represents a comparatively rare implementation of a light‐driven energy ratchet mechanism operating within a single covalently connected molecule [37]. It further provides a conceptual blueprint for light‐driven, kinetically trapped helicity control in adaptive chiral molecular systems and for the generation of out‐of‐equilibrium dynamic structures.

## Author Contributions

**Eric Sidler**: conceptualization, funding acquisition, investigation, project administration, visualization, writing – original draft, writing – review and editing. **Jinghao Wang**: investigation, visualization, writing – original draft, writing – review and editing. **Christina Vallina**: investigation, writing – review and editing. **Ben L. Feringa**: conceptualization, funding acquisition, project administration, supervision, writing – review and editing.

## Conflicts of Interest

The authors declare no conflicts of interest.

## Supporting information




**Supporting File 1**: anie74233‐sup‐0001‐SuppMat.pdf.

**Supporting File 2**: anie74233‐sup‐0002‐SuppMat.zip.

## Data Availability

The data that support the findings of this study are available in the Supplementary material of this article.
