# Supplementary material for: Light‐Driven Out‐of‐Equilibrium Helicity Control in a Diarylethene–Helicene Photosystem
Source: Angew Chem Int Ed Engl. 2026 Aug 19;65(45):e5252700. doi: 10.1002/anie.5252700 (PMC13725563; doi:10.1002/anie.5252700)
Supplement: Supplementary file 1 [file ANIE-65-e5252700-s002.pdf]

Supporting Information

Light-Driven Out-of-Equilibrium Helicity Control in a Diarylethene–Helicene Photosystem

Eric Sidler,*[a,b] Jinghao Wang,[a] Christina Vallina[a] and Ben L. Feringa*[a]

*To whom correspondence should be addressed

b.l.feringa@rug.nl

eric.sidler@chem.uzh.ch

[a] Stratingh Institute for Chemistry, University of Groningen, Nijenborgh 3, 9747 AG Groningen (The Netherlands)

[b] Current affiliation: Department of Chemistry, University of Zurich, Winterthurerstrasse 190, 8057 Zürich (Switzerland)

Table of Contents

General Remarks 3

Synthesis & Analytical Data 4

HPLC Separation 8

UV-Vis and CD Spectroscopy 10

Quantum Yield of Photoswitching 15

NMR Irradiation Experiments 20

Computational Analysis 35

References 42

General Remarks

NMR spectra were recorded on a Bruker Avance Neo with Cryoprobe Prodigy BBO (^{1}H: 600 MHz, ^{13}C: 151 MHz), Varian Mercury Plus (^{1}H: 500 MHz) and a Varian Mercury Plus (^{1}H: 400 MHz) instrument. Chemical shifts (δ) are in parts per million (ppm) relative to TMS. Irradiation experiments were performed using fiber-coupled LEDs (340 nm, 365 nm and 505 nm) powered with a T-CubeTM LEDD1B driver obtained from Thorlabs Inc. Photon flux of the 340 nm, 365 nm and 505 nm LEDs were determined to be $6.5\cdot10^{-6}\cdot mE\cdot s^{-1}$, $9.0\cdot10^{-5}\cdot mE\cdot s^{-1}$ and $1.9\cdot10^{-4}\cdot mE\cdot s^{-1}$ for the UV-Vis and CD measurements, and $2.3\cdot10^{-5}\cdot mE\cdot s^{-1}$, $4.2\cdot10^{-4}\cdot mE\cdot s^{-1}$ and $2.7\cdot10^{-4}\cdot mE\cdot s^{-1}$ for the in-situ NMR irradiation experiments, respectively. HPLC analysis was performed using a Shimadzu Prominence System equipped with a diode array detector using CHIRALPAK columns (Daicel Corporation) and a column temperature of 40 °C. UV/Vis absorption spectra were recorded on an Agilent Cary 8454 spectrophotometer in a 1 cm quartz cuvette. CD spectra were obtained on a Jasco J715 spectropolarimeter. DFT calculations were performed using the Orca 6.1.0. package.[1]

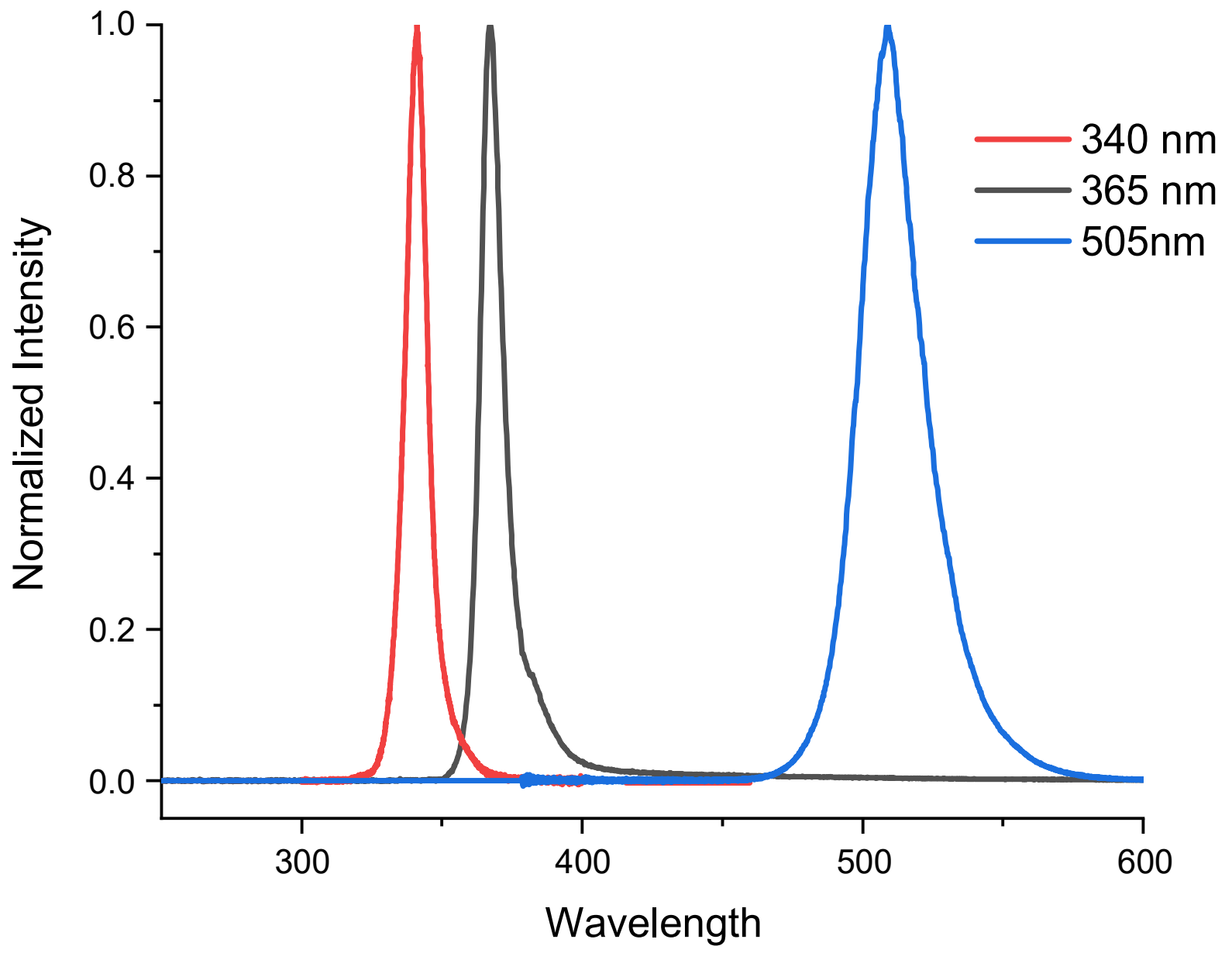

Figure S 1. Emission profile of the used LEDs 340 nm (red), 365 nm (black) and 505nm (blue).

Synthesis & Analytical Data

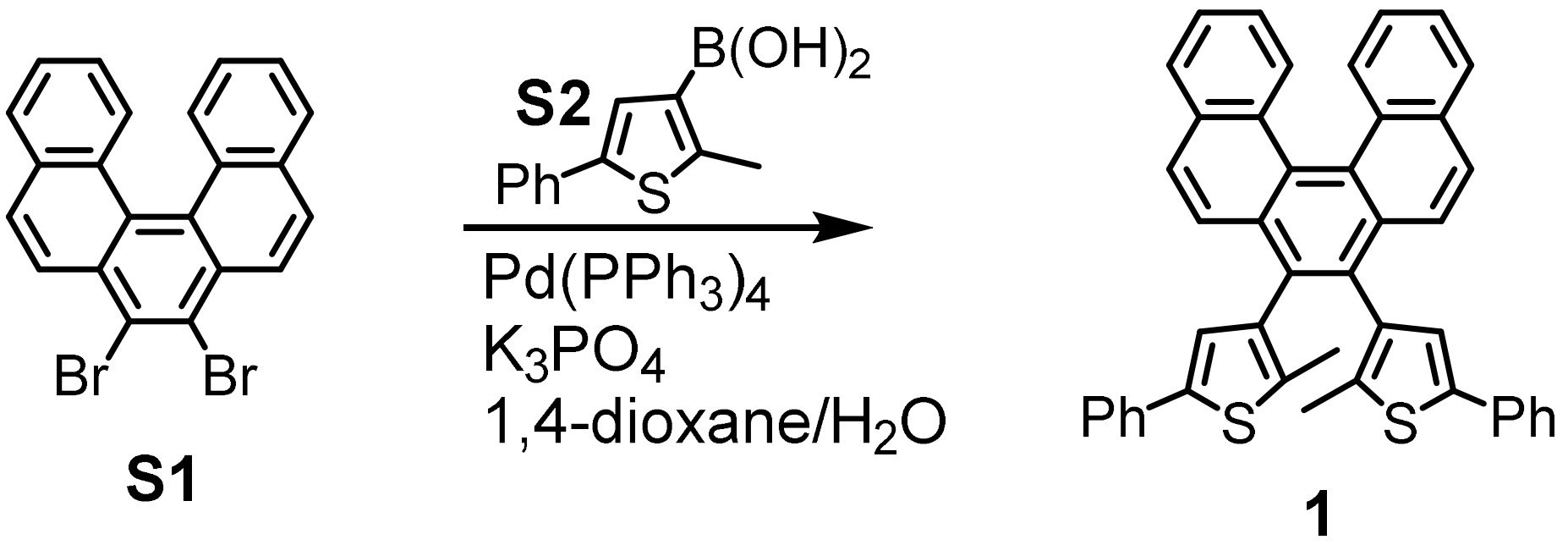

Switch **1** was synthesized according to a reported literature procedure.[2] Both starting materials **S1**[3] and **S2**[4] were synthesized according to literature procedures.

The analytical data of the obtained thermally relaxed composition of switch **1** matched the literature report (Figure S2, S3 and S4).[2]

The individual diastereomeric pair of enantiomers **M1p**, **M1ap** and **P1ap** of switch **1** were separated via HPLC.

M1ap:

^{1}H NMR (400 MHz, $CDCl_3$) δ 8.49 (d, *J* = 8.6 Hz, 2H), 7.95 (d, *J* = 8.0 Hz, 2H), 7.87 (d, *J* = 8.9 Hz, 2H), 7.73 (d, *J* = 8.8 Hz, 2H), 7.53 (t, *J* = 7.4 Hz, 2H), 7.48 (dd, *J* = 7.5, 1.6 Hz, 4H), 7.38 – 7.27 (m, 6H), 7.22 (t, *J* = 7.4 Hz, 2H), 7.00 (s, 2H), 2.23 (s, 6H).

P1ap:

^{1}H NMR (400 MHz, $CDCl_3$) δ 8.46 (d, *J* = 8.5 Hz, 2H), 8.07 – 7.92 (m, 2H), 7.86 (d, *J* = 8.9 Hz, 2H), 7.71 (d, *J* = 8.9 Hz, 2H), 7.58 – 7.46 (m, 6H), 7.41 – 7.20 (m, 8H), 7.05 (s, 1H), 2.12 (s, 6H).

M1p:

^{1}H NMR (400 MHz, $cdcl_3$) δ 8.50 (d, *J* = 8.5 Hz, 1H), 8.47 (d, *J* = 8.6 Hz, 1H), 7.96 (t, *J* = 7.8 Hz, 2H), 7.88 (dd, *J* = 8.9, 3.6 Hz, 2H), 7.80 (dd, *J* = 8.8, 2.4 Hz, 2H), 7.57 – 7.48 (m, 4H), 7.41 (d, *J* = 7.6 Hz, 2H), 7.34 – 7.17 (m, 8H), 7.14 (s, 1H), 6.97 (s, 1H), 2.20 (s, 3H), 2.00 (s, 3H).

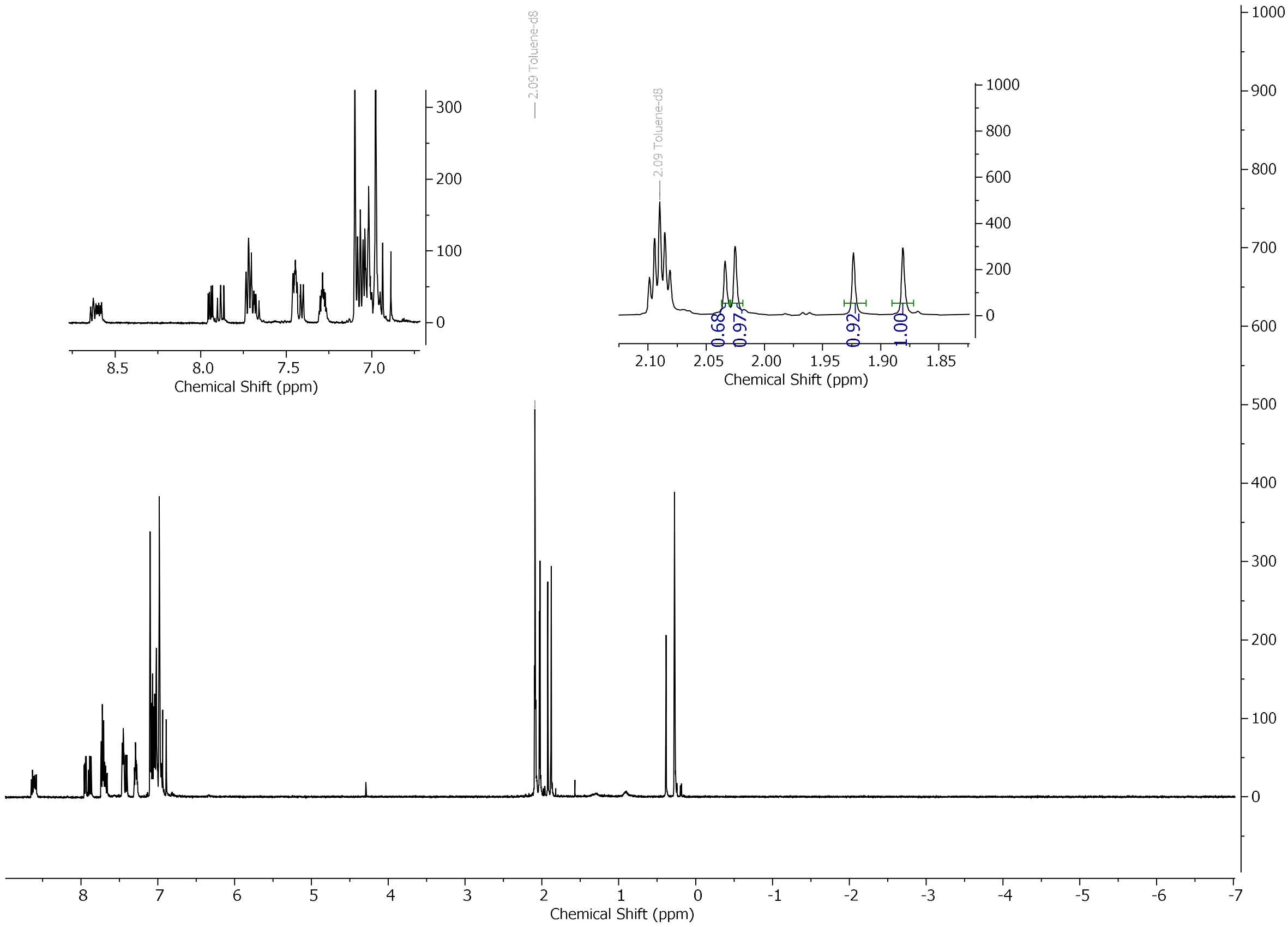

Figure S 2. ^{1}H NMR spectrum of a thermally relaxed mixture of isomers of **1** (500 MHz, toluene-d_8, 25 °C).

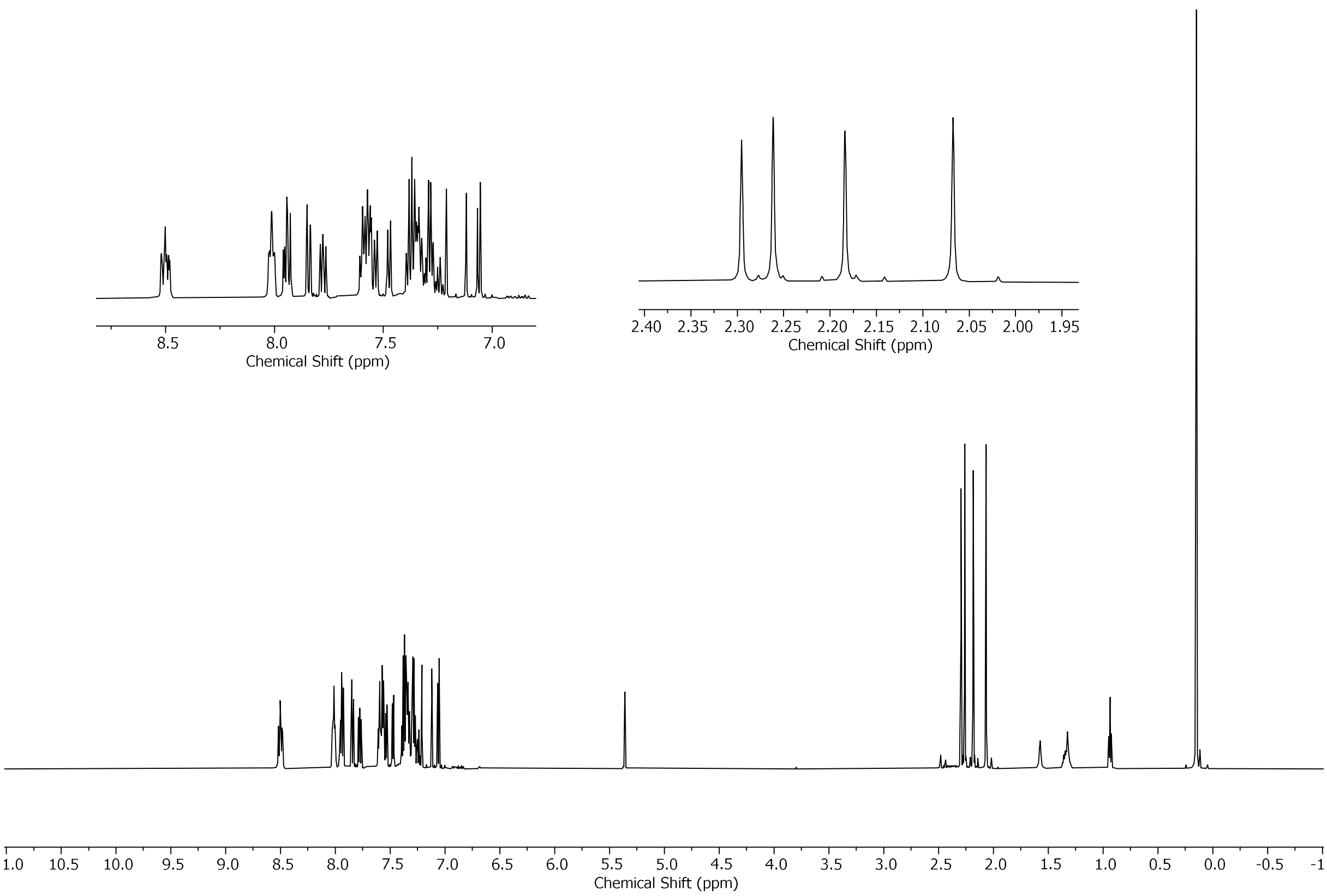

Figure S 3. ^{1}H NMR spectrum of a thermally relaxed mixture of isomers of **1** (600 MHz, CD_2Cl_2, 25 °C).

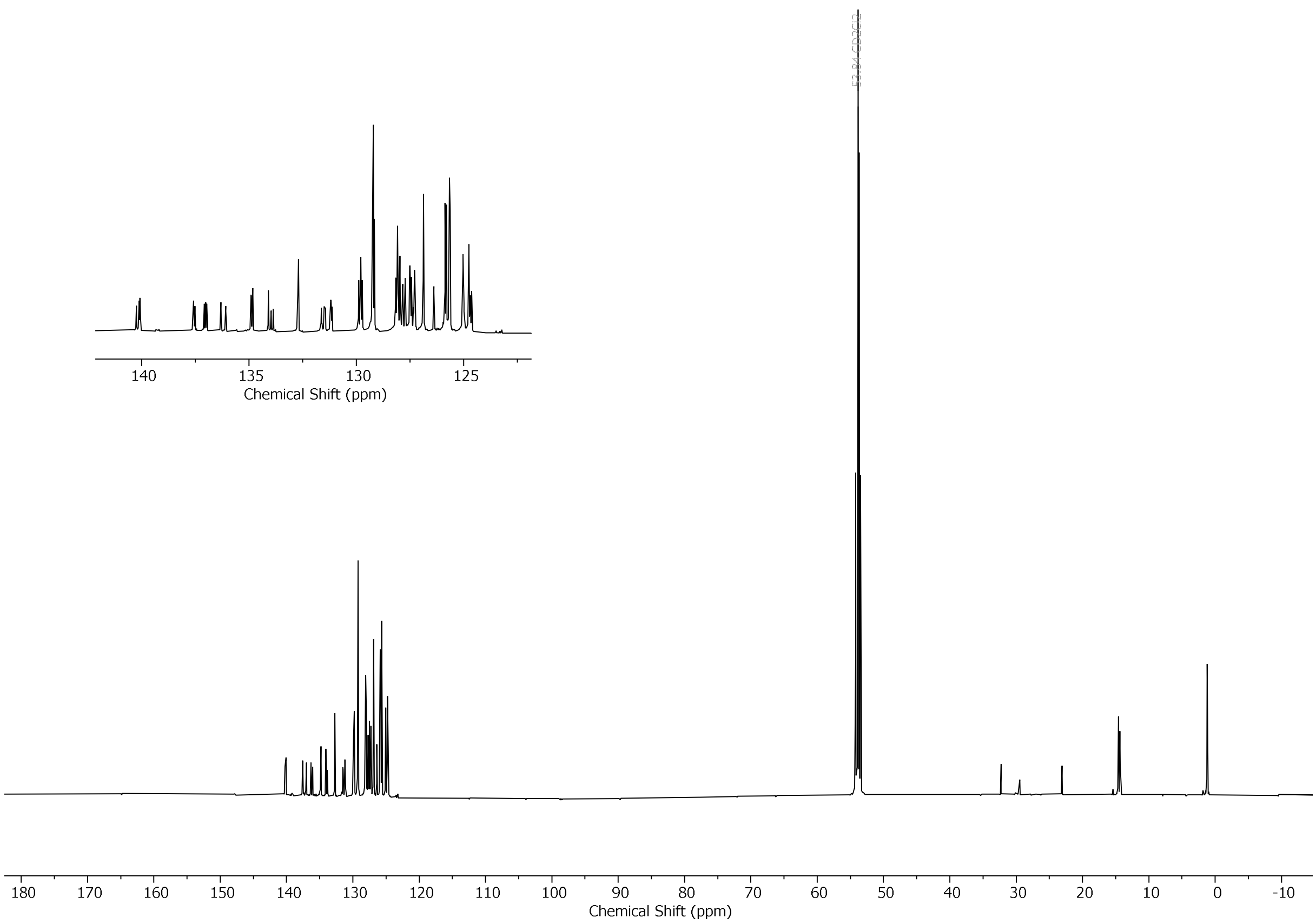

Figure S 4. ^{13}C NMR spectrum of a thermally relaxed mixture of isomers of **1** (151 MHz, CD_2Cl_2, 25 °C).

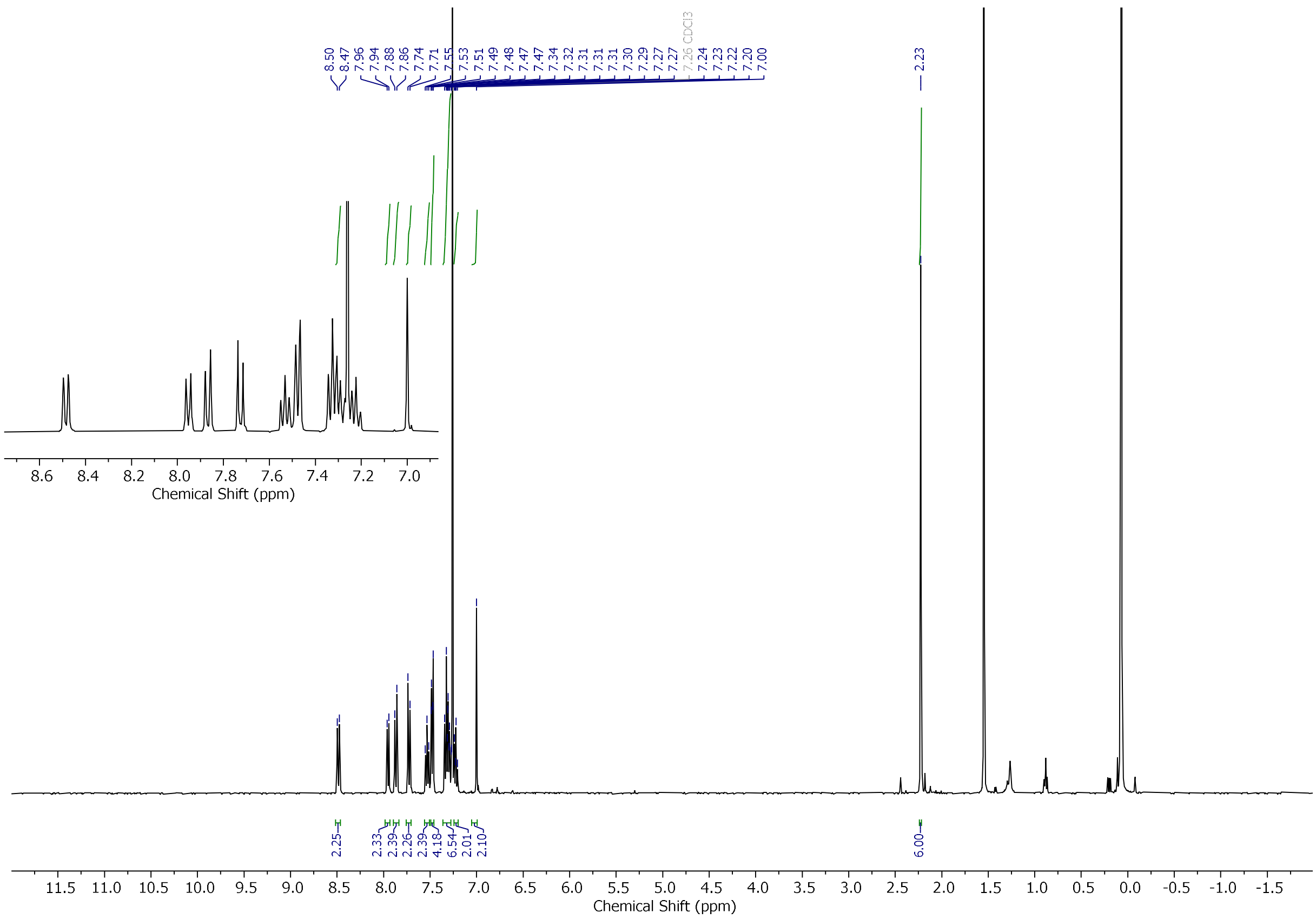

Figure S 5. ^{1}H NMR spectrum of **M1ap** isolated via HPLC (400 MHz, $CDCl_3$, 25 °C).

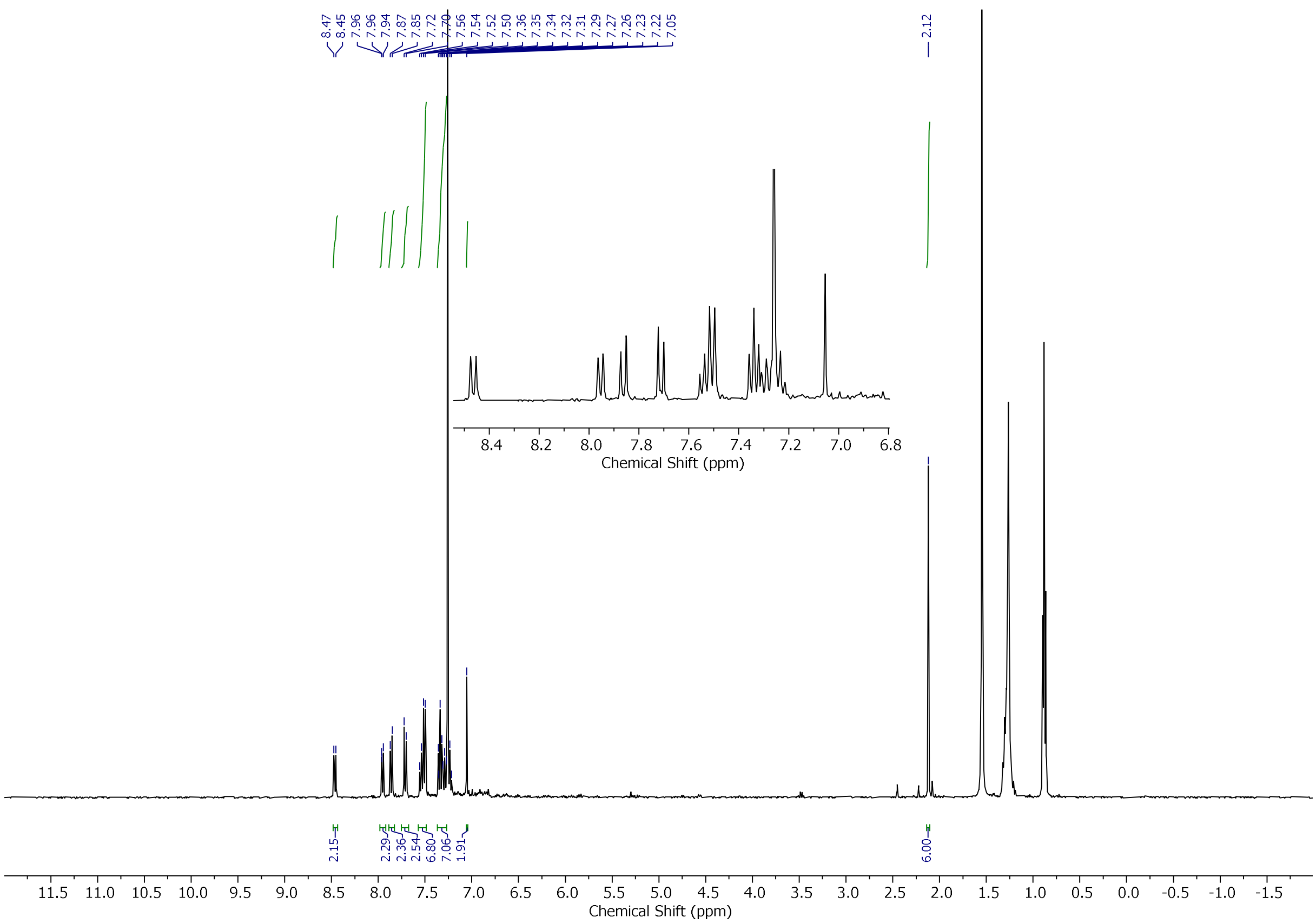

Figure S 6. ^{1}H NMR spectrum of **P1ap** isolated via HPLC (400 MHz, $CDCl_3$, 25 °C).

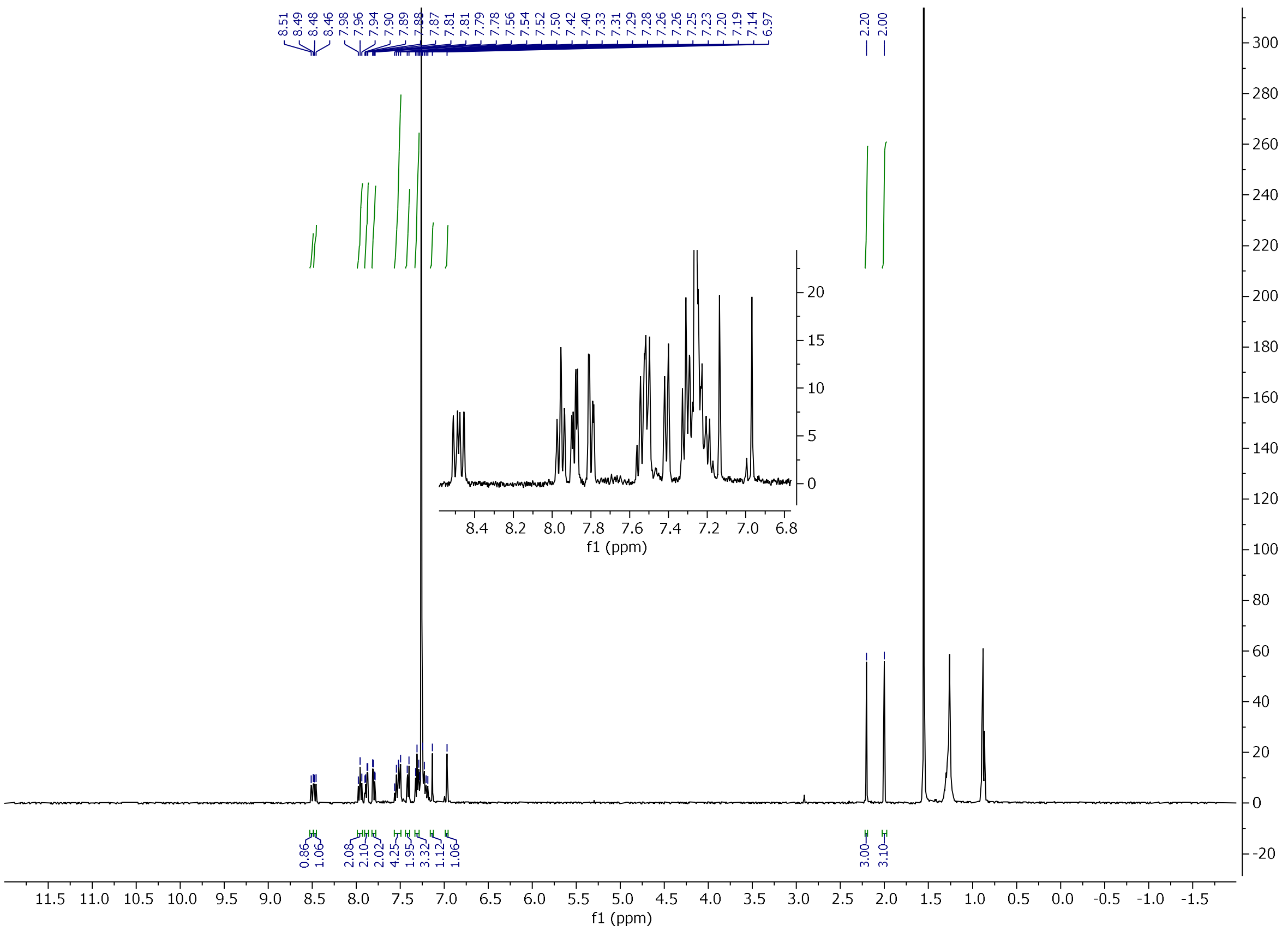

Figure S 7. ^{1}H NMR spectrum of **M1p** isolated via HPLC (400 MHz, $CDCl_3$, 25 °C).

HPLC Separation

Separation of **1** into different isomers was achieved using HPLC on a chiral stationary phase. More precisely, a solution of **1** in *n*-heptane was subjected to separation conditions on a semipreparative CHIRALPAK IA column (10 x 250 mm, 5µm particle size), using *n*-heptane/isopropanol 99:1 as a mobile phase (Figure S8). The first two peaks correspond to the two enantiomers of **M1ap** (see maintext for denotation). Both peaks were collected collectively to maximize the quantity obtained. The two succeeding peaks correspond to a mixture of **P1ap** and **M1p** and were poorly resolved, which is why they were collected collectively as well. **P1ap** can be obtained at a decent purity, when the peaks are cut-off manually during the separation procedure. This was done for the irradiation experiment at −80 °C of **P1ap.**

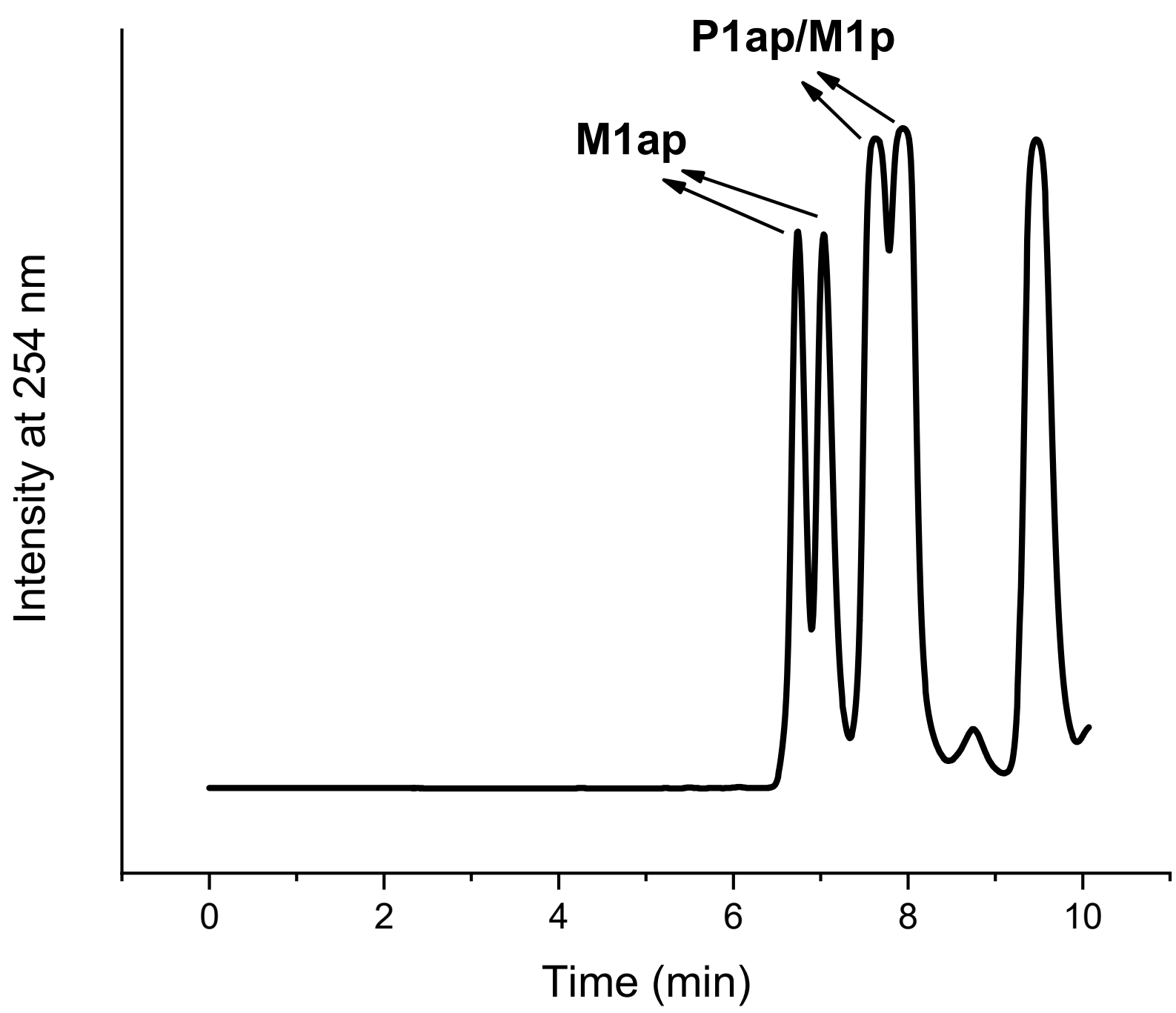

Figure S 8. HPLC chromatogram of a mixture of isomers of **1**. Conditions: CHIRALPAK IA, *n*-heptane/isopropanol 99 :1.

Separation into all enantiopure species can be achieved using a less polar mobile phase, as previously reported (Figure S9),[2] which was done for CD analysis (see Figure S16 and S17). However, the elution time and thus quantity of solvent needed for semipreparative separation increases significantly, which is why this was not done for NMR experiments. Separation using HPLC on an achiral column material was unsuccessful.

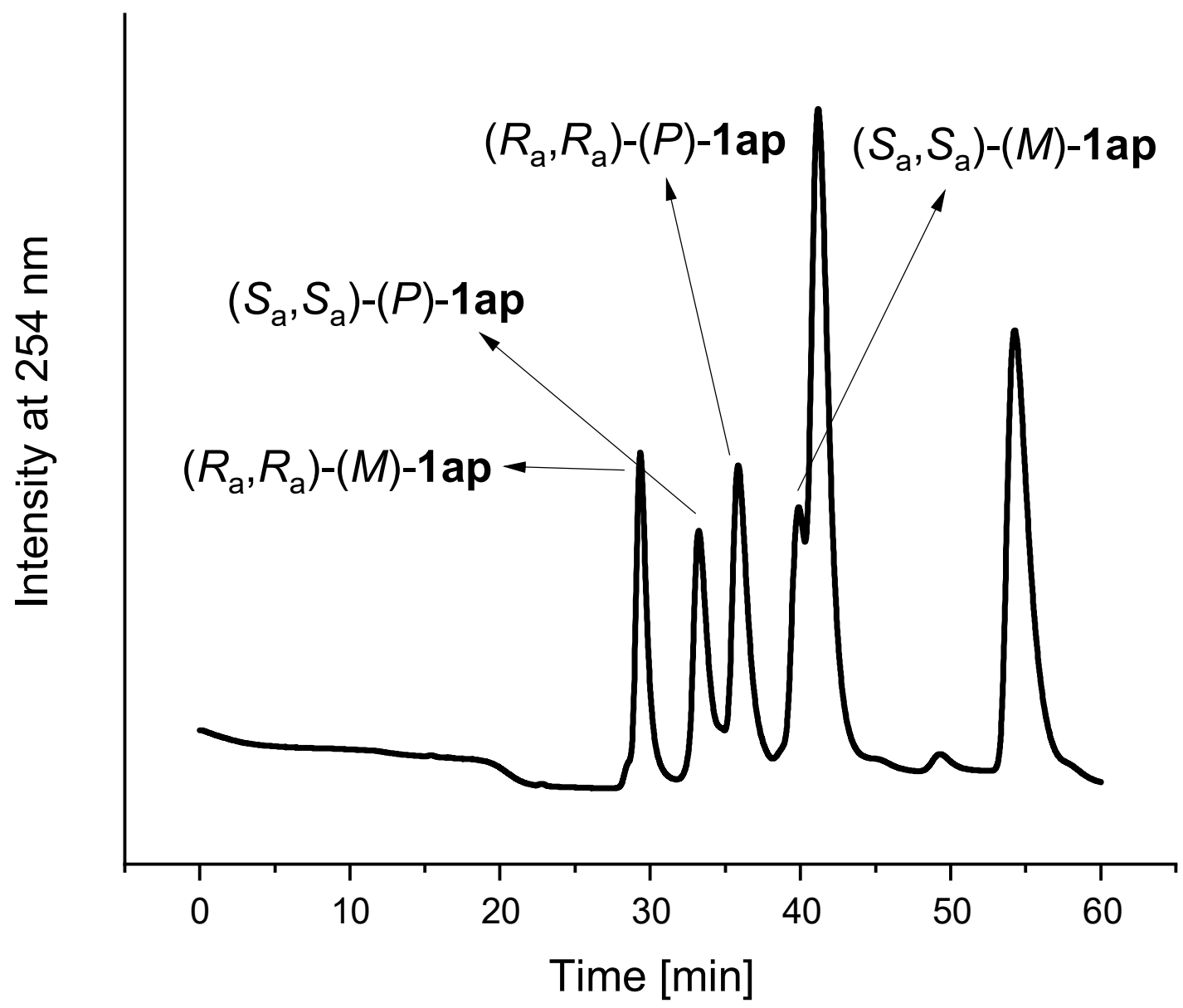

Figure S 9. HPLC chromatogram of a mixture of isomers of **1**. Conditions: CHIRALPAK IA, *n*-heptane/isopropanol 99.5 :0.5.

UV-Vis and CD Spectroscopy

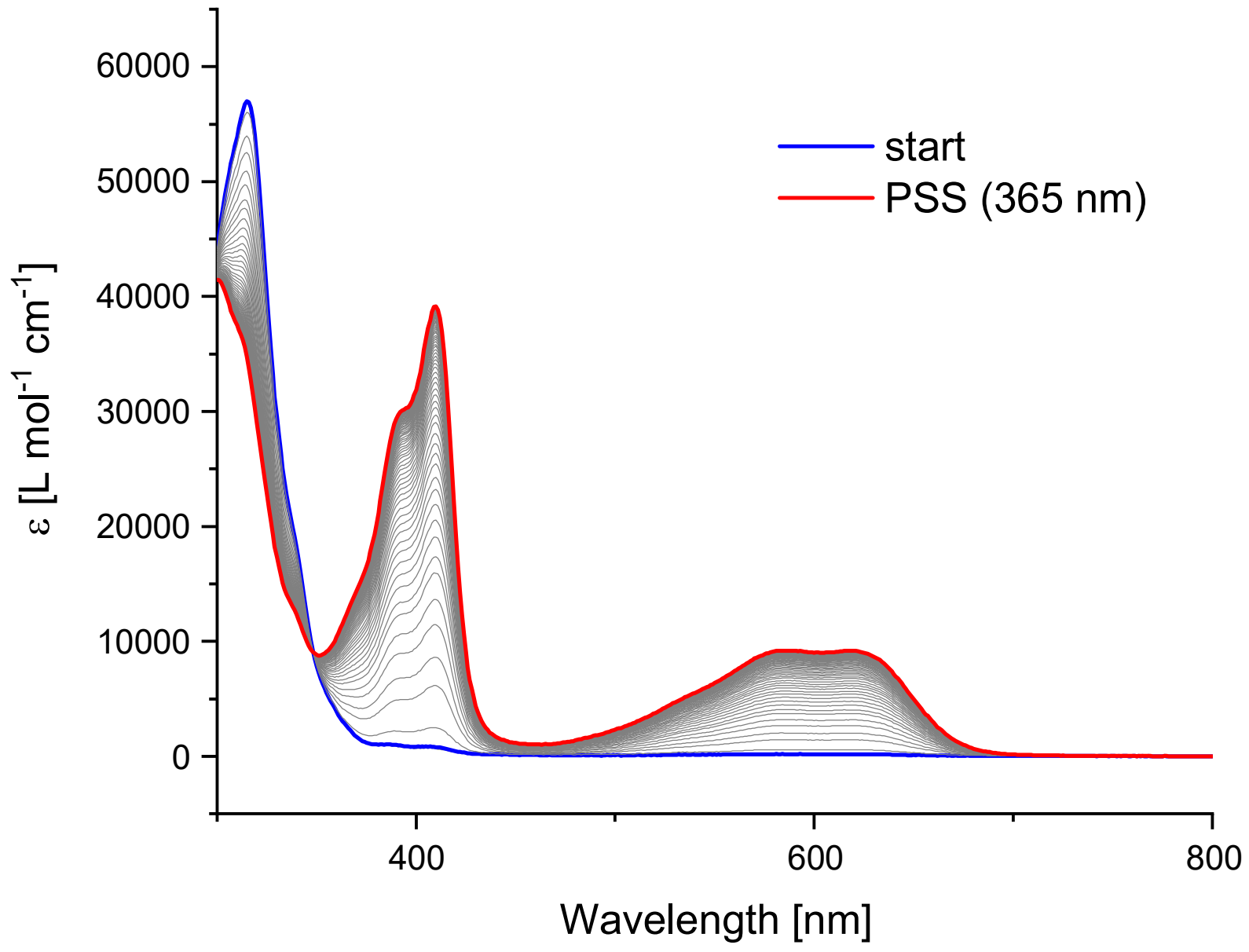

Figure S 10. Changes in the UV-Vis absorption spectrum of **M1ap** upon irradiation with 365 nm at 20 °C in toluene (c ~ 10^{-5} M).

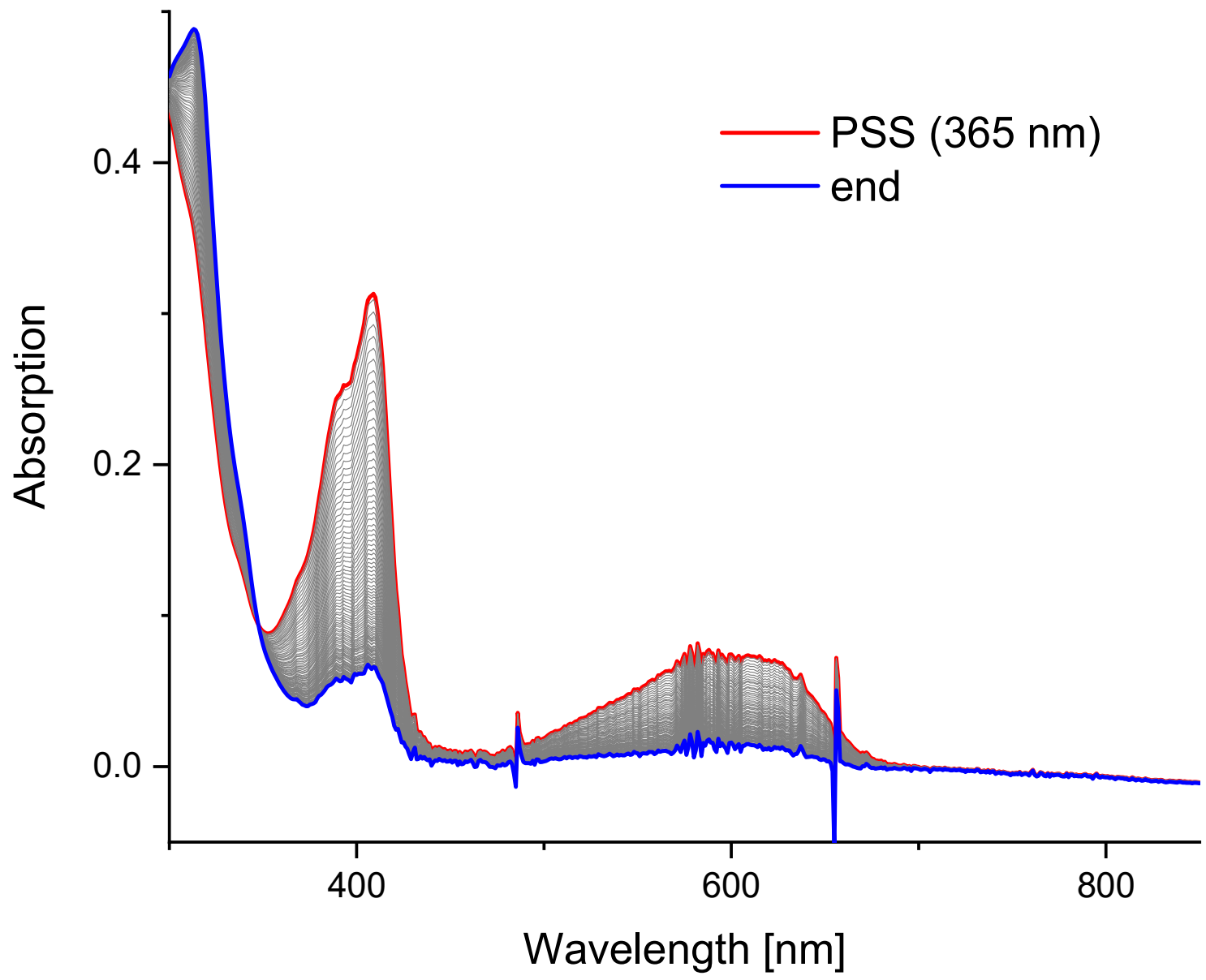

Figure S 11. Changes in the UV-Vis absorption spectrum upon thermal ring-opening from **M1c** to **M1ap** at 20 °C in toluene (c ~ 10^{-5} M).

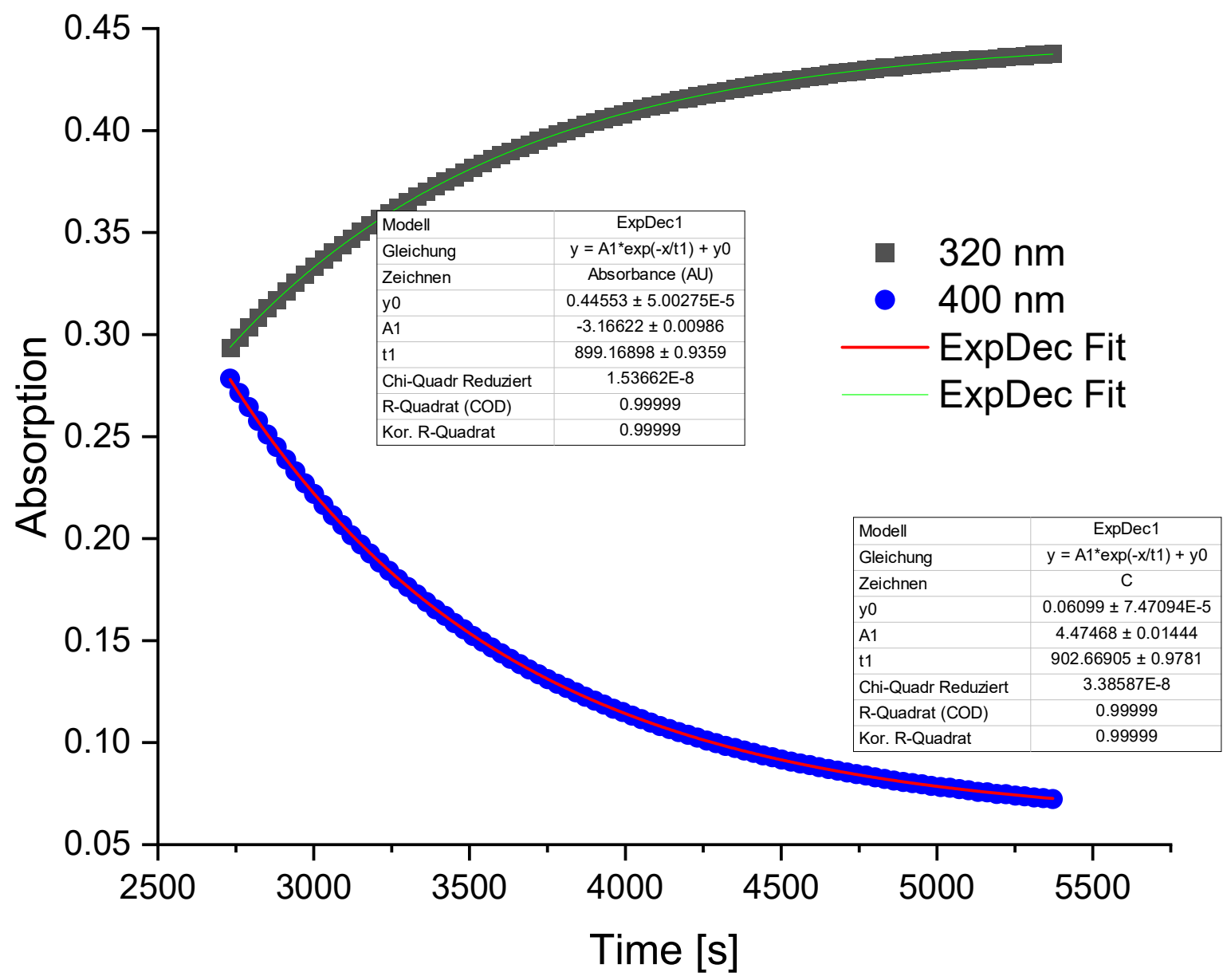

Figure S 12. Time-traces of the absorption values at 400 nm (blue) and 320 nm (black) upon thermal ring-opening of **M1c** to **M1ap** at 20°C. The exponential decay fit parameters can be found in the table within the plot.

With

$$k = \frac{1}{t1}$$

and

$$\Delta G_T^{\ddagger} = -RTln\left(\frac{hk}{Tk_B}\right)$$

the barrier for the thermal backswitching of **M1c** was determined to be

$$\Delta G_{293.15}^{\ddagger} = 88\ kJ/mol$$

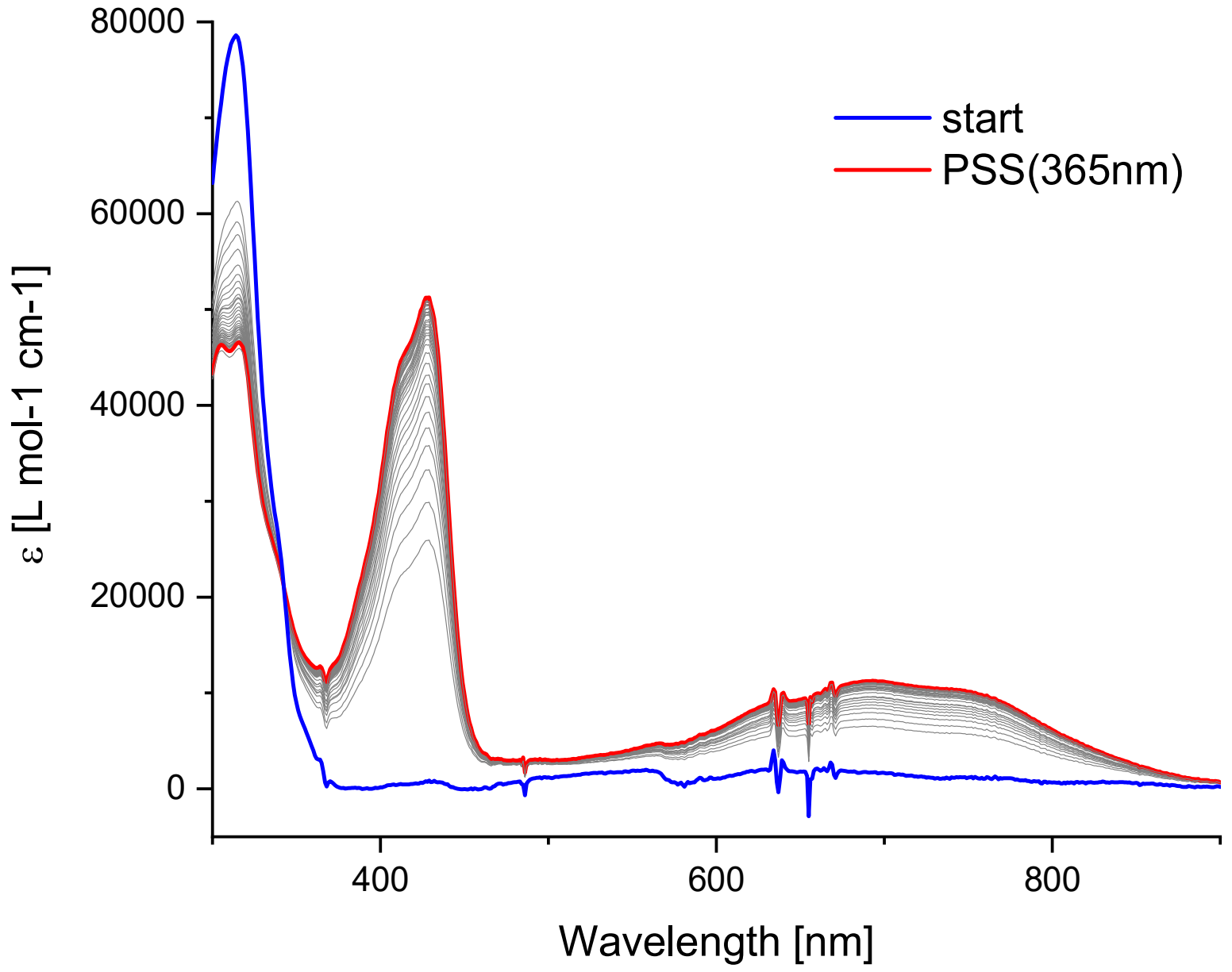

Figure S 13. Changes in the UV-Vis absorption spectrum of **P1ap** upon irradiation with 365 nm at −80 °C in toluene (c ~ 10^{-5} M).

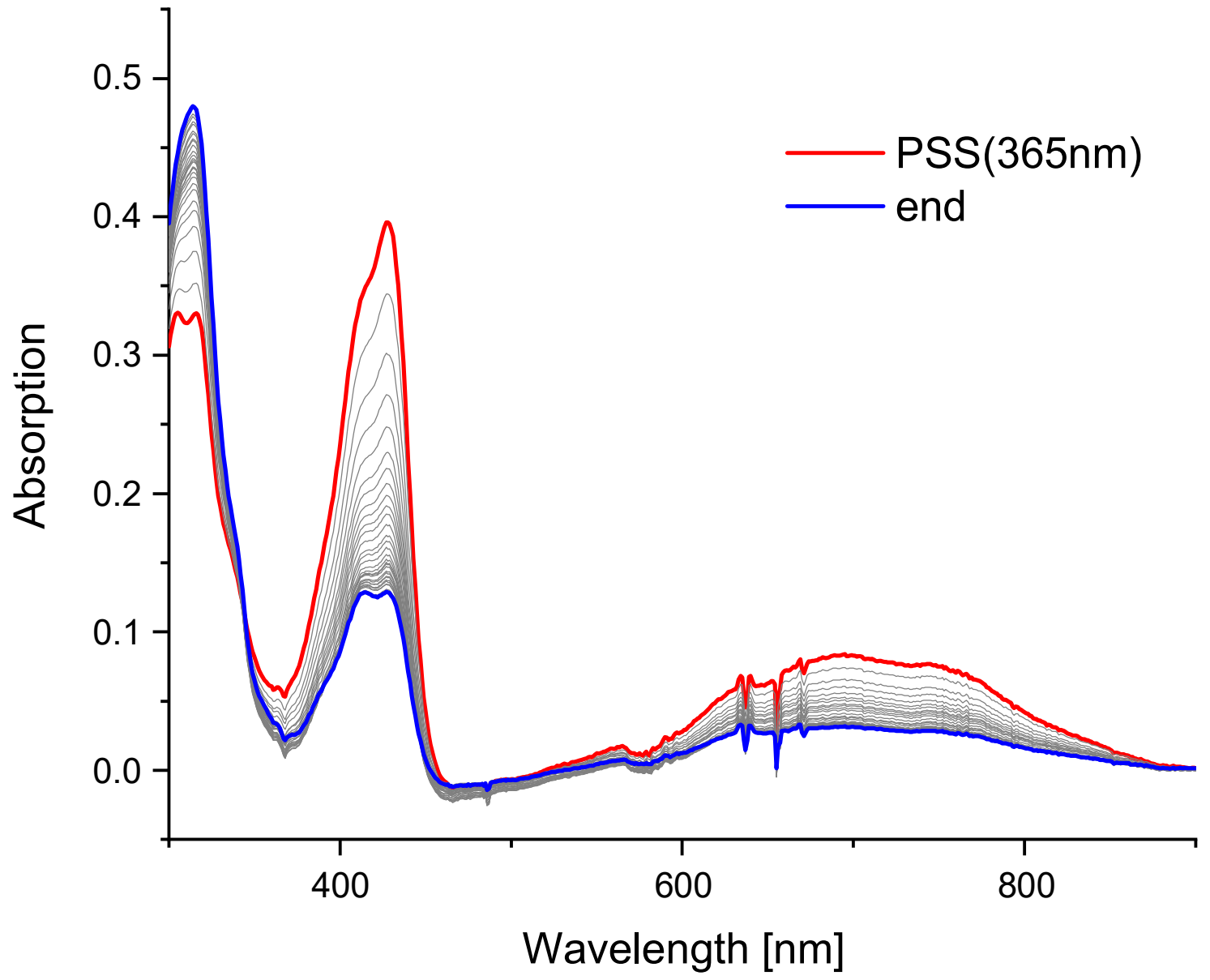

Figure S 14. Changes in the UV-Vis absorption spectrum upon thermal ring-opening from **P1c** to **P1ap** at −80 °C in toluene (c ~ 10^{-5} M).

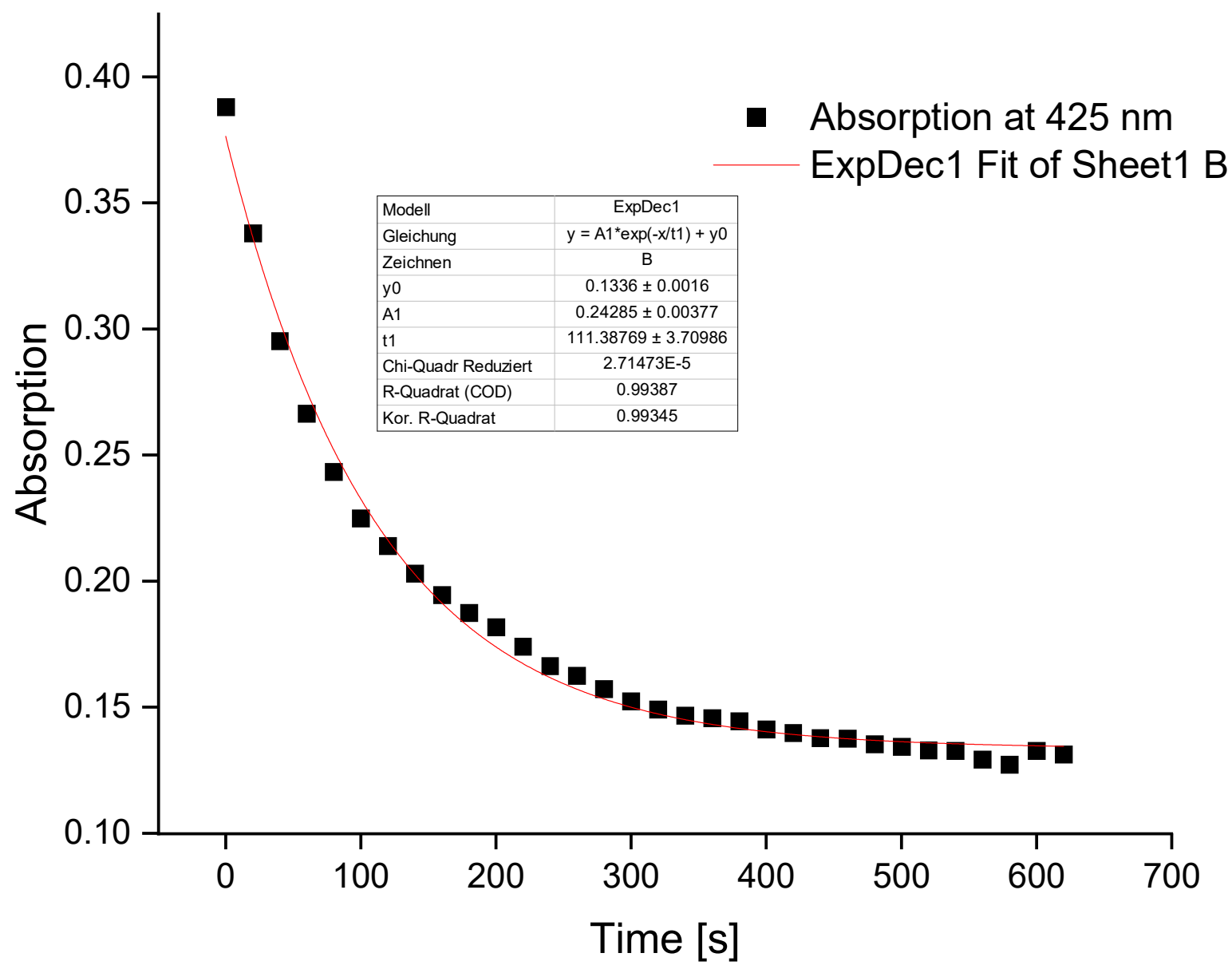

Figure S 15. Time-traces of the absorption values at 425 nm upon thermal ring-opening of **P1c** to **P1ap** at −80 °C. The exponential decay fit parameters can be found in the table within the plot.

With

$$k = \frac{1}{t1}$$

and

$$\Delta G_T^{\ddagger} = -RTln\left(\frac{hk}{Tk_B}\right)$$

the barrier for the thermal backswitching of **P1c** was determined to be

$$\Delta G_{193.15}^{\ddagger} = 54\ kJ/mol$$

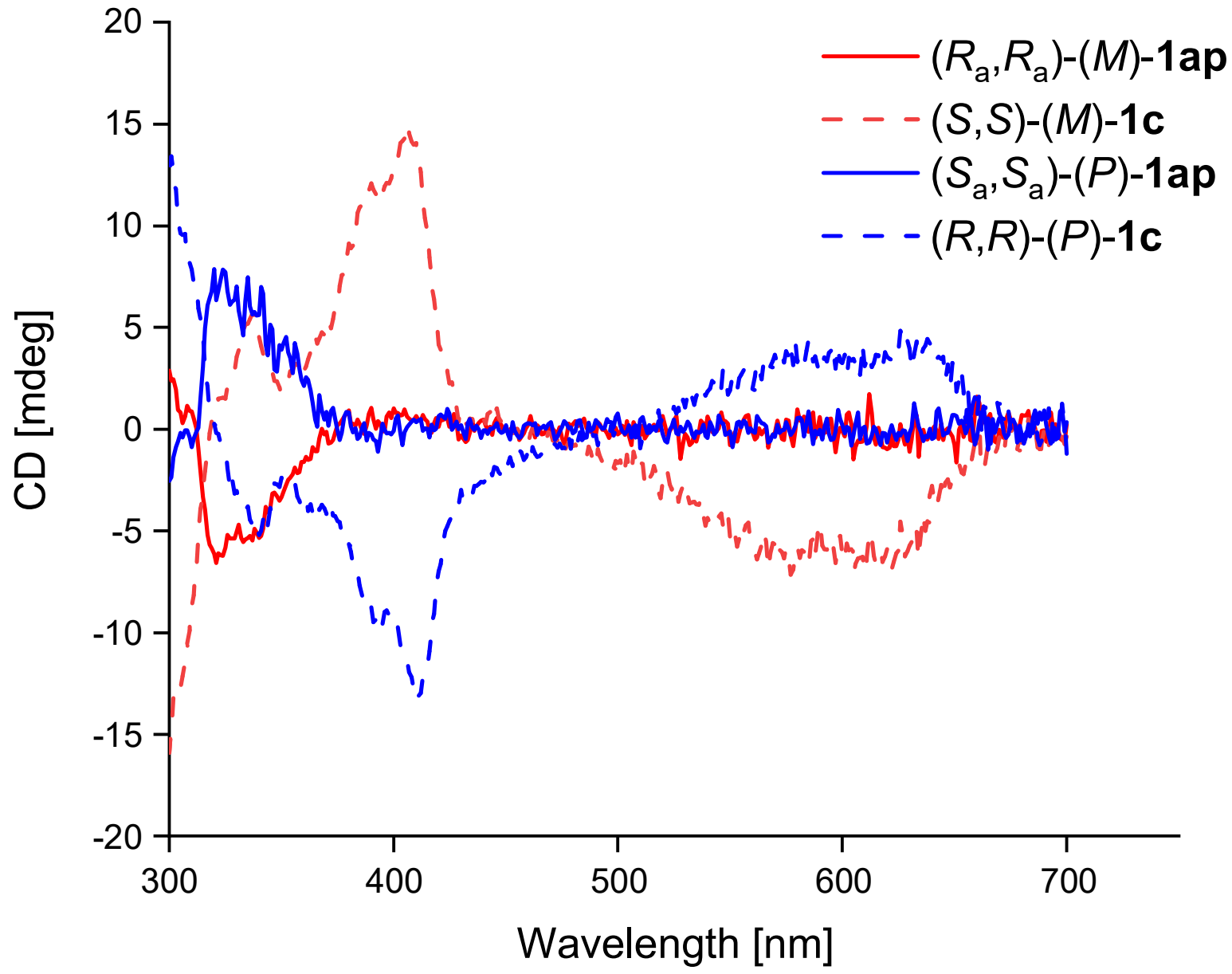

Figure S 16. CD spectra of (R_a,R_a)-(*M*)-**1ap** (red), (*S*,*S*)-(*M*)-**1c** (red, dashed), (S_a,S_a)-(*P*)-**1ap** (blue) and (*R*,*R*)-(*P*)-**1c** (blue, dashed) in toluene at 20°C. Assignment of the enantiomers was performed according to TDDFT calculations and comparing their CD spectra (see Figure S41 and S42). The enantiomers were isolated using the HPLC conditions reported in Figure S9, and the respective closed species were formed by irradiation at 20°C.

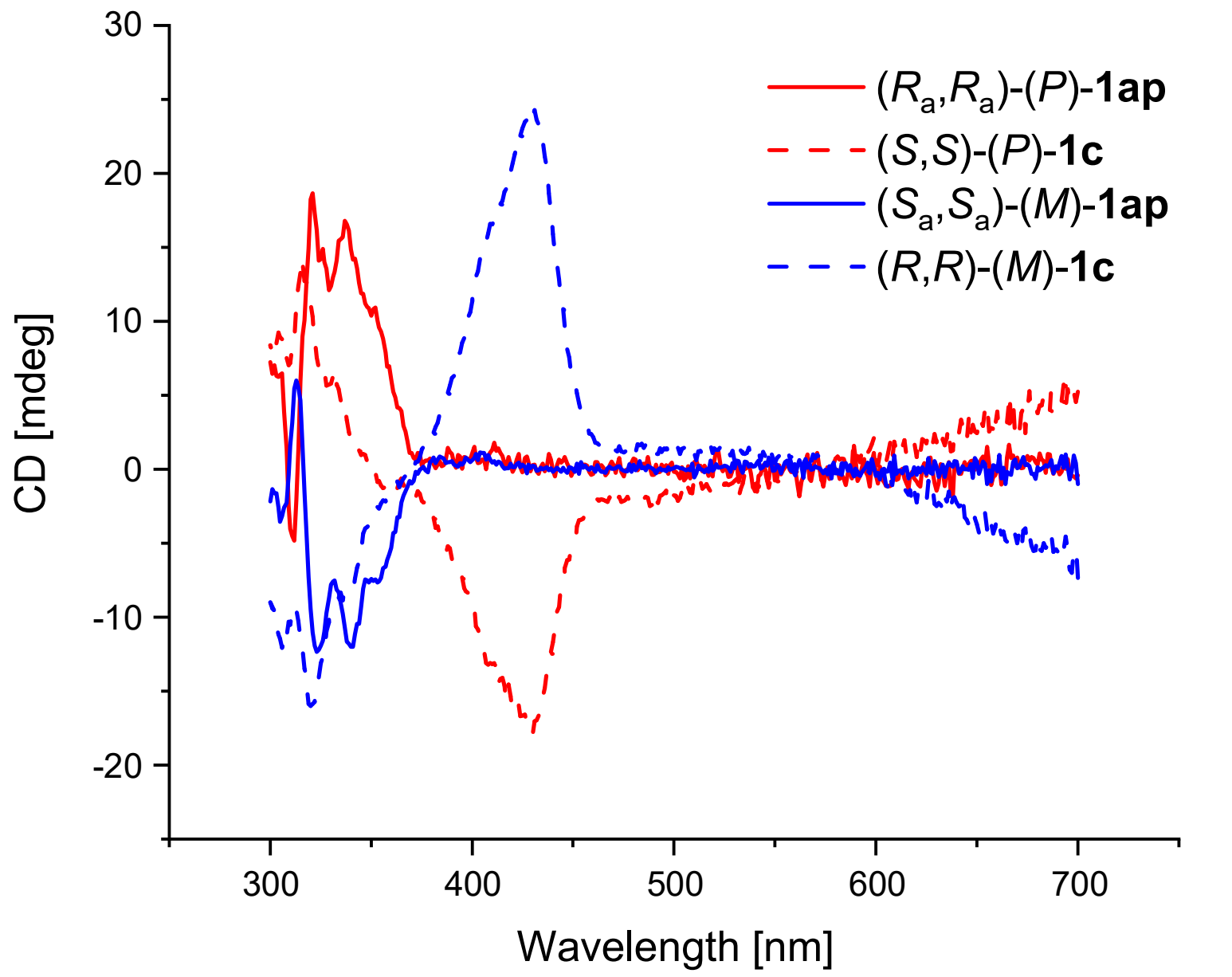

Figure S 17. CD spectra of (R_a,R_a)-(*P*)-**1ap** (red), (*S*,*S*)-(*P*)-**1c** (red, dashed), (S_a,S_a)-(*M*)-**1ap** (blue) and (*R*,*R*)-(*M*)-**1c** (blue, dashed) in toluene at 20°C. Assignment of the enantiomers was performed according to TDDFT calculations and comparing their CD spectra (see Figure S43 and S44). The enantiomers were isolated using the HPLC conditions reported in Figure S9, and the respective closed species were formed by irradiation at −80 °C.

Quantum Yield of Photoswitching

Ferrioxalate Chemical Actinometry

A modification of a standard protocol was applied for the determination of the photon flux.[5,6] An aqueous H_2SO_4 solution (0.05 M) containing freshly recrystallized $K_3[Fe(C_2O_4)_3]$ (41 mM, 2 mL, 1 cm quartz cuvette) was irradiated at 20 °C for a given period in the dark with an LED at 395/415/420/455 nm. The solution was then diluted with 1.0 mL of an aqueous H_2SO_4 solution (0.5 M) containing phenanthroline (1 g/L) and NaOAc (122.5 g/L) and left to react for 10 min. The absorption at λ= 510 nm was measured and compared to an identically prepared non-irradiated sample. The concentration of $[Fe(phenanthroline)_3]^{2+}$ complex was calculated using its molar absorptivity (ε = 11100 M^{-1} cm^{-1}) and considering the dilution. The quantity of Fe^{2+} ions expressed in mol was plotted versus time (expressed in seconds) and the slope, obtained by linear fitting the data points to the equation y = ax +b, equals the rate of formation of the Fe^{2+} ion at the given wavelength. This rate can be converted into the photon flux (I) by dividing it by the quantum yield of $[Fe(phenanthroline)_3]^{2+}$ complex at the wavelength of interest (Φ^{365nm} = 1.20) and by the probability of photon absorption of the Fe^{3+} complex (approximated to 1 as we were working in the total absorption regime). The obtained molar photon fluxes were (I^{365nm} = $9.0474 \cdot 10^{-5}$ mmol s^{-1} for the room temperature set-ups and I^{365nm} = $4.884 \cdot 10^{-5}$ mmol s^{-1} for the cryogenic set-ups). These values are calculated assuming a single wavelength light source. Due to the possible wavelength dependency of the quantum yields, this point represents a source of uncertainty.

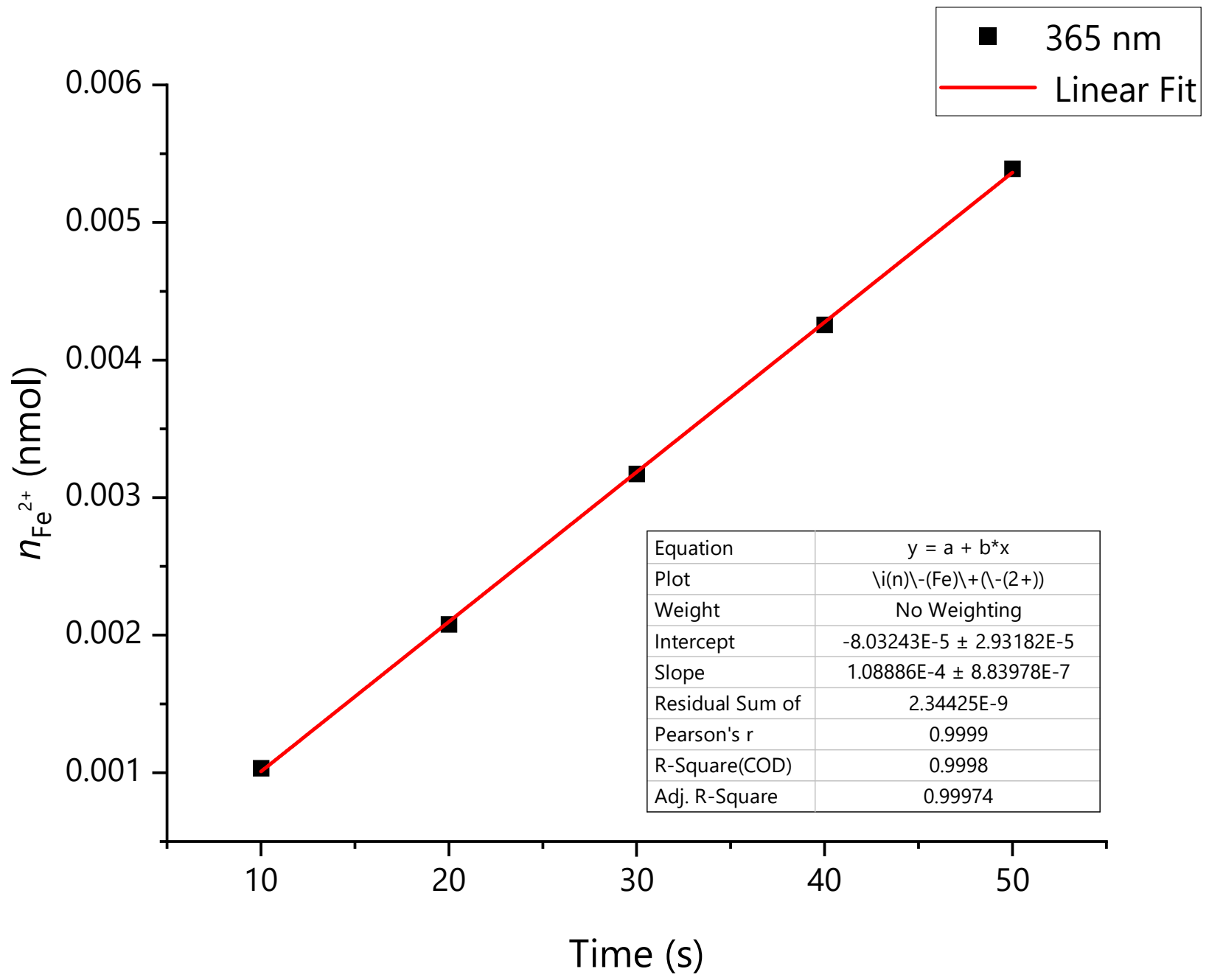

Figure S 18. Linear fitting of the Fe^{2+} moles generated upon irradiation of the $[Fe(C_2O_4)_3]^{2+}$ complex 365 nm for the room temperature set-ups at different irradiation times.

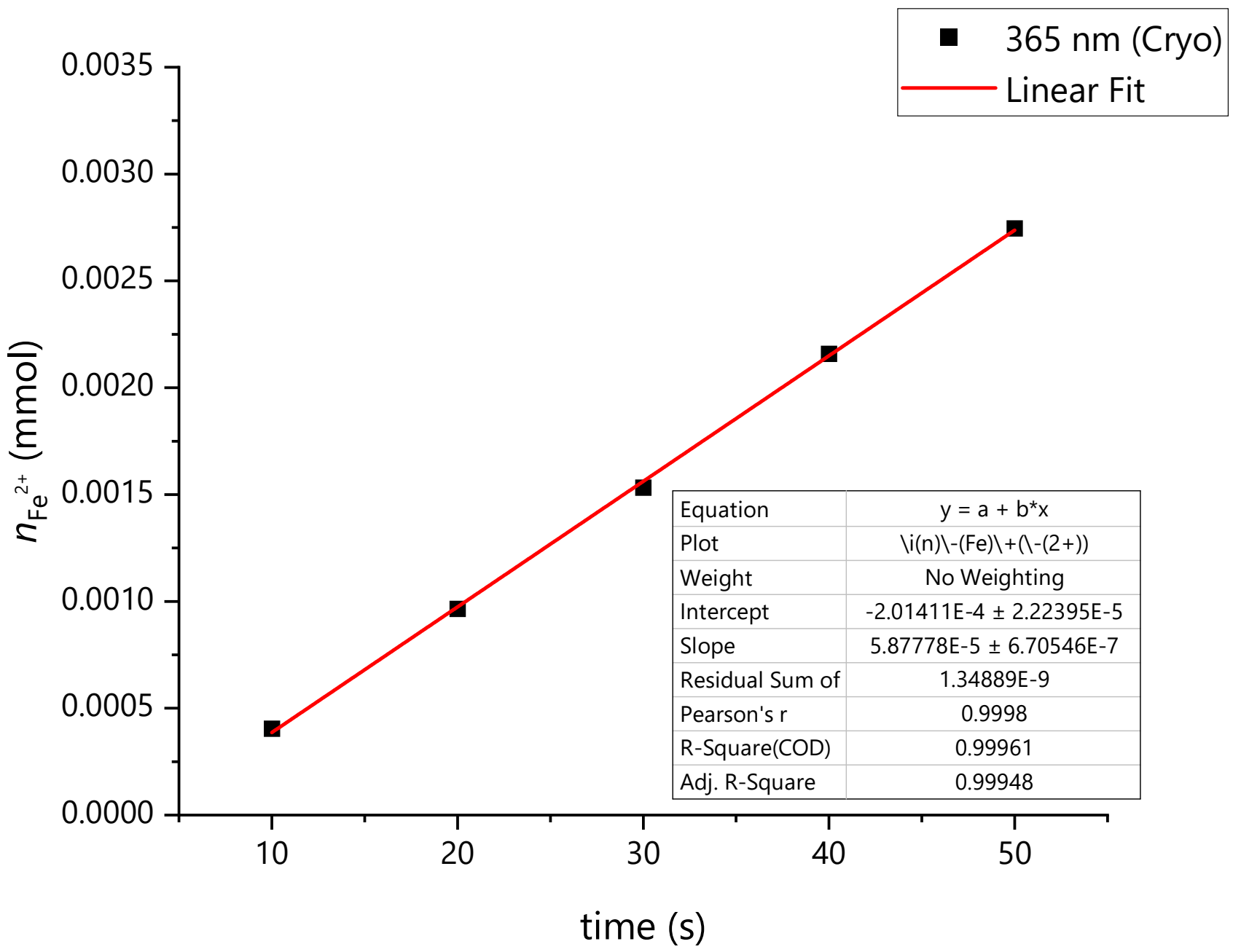

Figure S 19. Linear fitting of the Fe^{2+} moles generated upon irradiation of the $[Fe(C_2O_4)_3]^{2+}$ complex 365 nm for the cryogenic set-ups at different irradiation times

Quantum Yield

Solutions of the open state (*o*) **M1ap** and **P1ap** (~ 10 μM) in toluene were irradiated with an LED of a selected wavelength (365nm) to lead to the formation of the ring-closed state (*c*). The spectra were collected following the evolution of the absorption at the wavelength of irradiation. Due to the different thermal stabilities of the two DAE derivatives, different experimental setups were used for the quantum yield measurements. The thermally stable compound **M1c** was measured using the standard room-temperature quantum yield setup, whereas the thermally less stable compound **P1c** was measured using a cryogenic setup at −80 °C to minimize thermal ring opening during irradiation and spectral monitoring. The data was subsequently fitted using COPASI 4.29[7] following the same approach developed by Stranius & Börjesson. Equiv. 14 in the original article

$$\frac{d[o]}{dt} = -\frac{QY_{o\to c} \cdot I \cdot \beta_o(t)}{N_A \cdot V} + \frac{QY_{c\to o} \cdot I \cdot \beta_c(t)}{N_A \cdot V} + k_{c\to o}[c]$$

was used to determine both approximated QYs ($QY_{o\to c}$ for the formation of the ring-closed state and $QY_{c\to o}$ for the opposite photochemical reaction). The kinetic constant $k_{c\to o}$ related to the thermal formation of the ring-open state. *I* is the photon flux, previously determined with ferrioxalate actinometry, N_A the Avogadro number, *V* the total volume of the irradiated solution (2 mL) and β the fractions of photons absorbed by either the open or the closed state. The decay was fitted by the ODE solver present in COPASI, using a Levenberg-Marquardt algorithm with randomized initial conditions. To obtain physically sound results, the boundaries for the QYs values were fixed between 10^{-6} and 1.

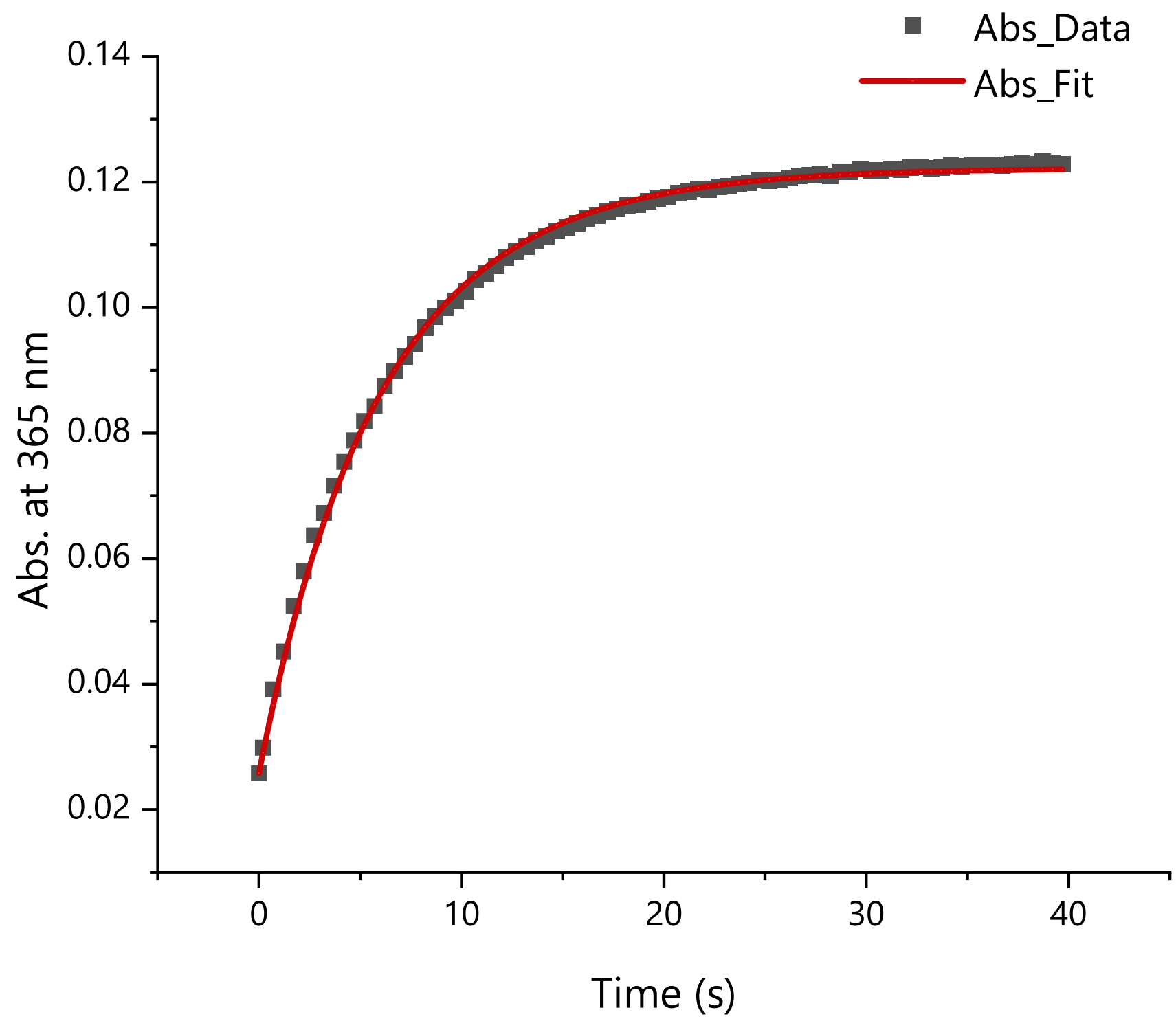

Figure S 20. Evolution of the absorption at 365 nm during the irradiation of **M1ap** in toluene at 365 nm at 20 ºC. The red line represents the fit obtained with the ODE solver from COPASI.

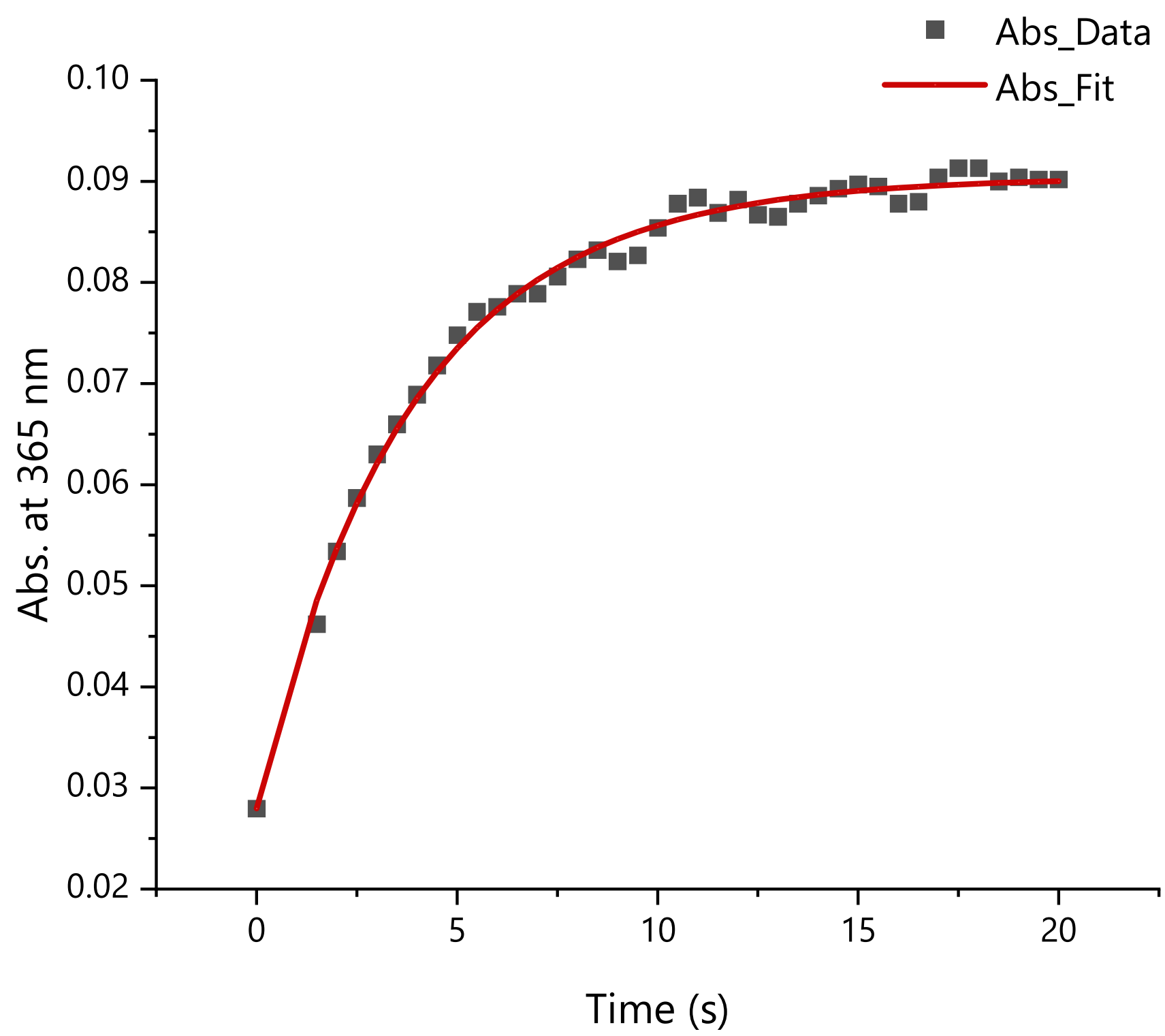

Figure S 21. Evolution of the absorption at 365 nm during the irradiation of **P1ap** in toluene at 365 nm at -80 ºC. The red line represents the fit obtained with the ODE solver from COPASI.

Table S 1. Quantum yield and photo-stationary state ratio of **M1ap** and **P1ap**. [a]

Compound	PSS ratio ($\boldsymbol{c}$: $\boldsymbol{o}$) [b]	$\boldsymbol{k_{c\rightarrow o}}$ [c]	$\boldsymbol{QY_{o\rightarrow c}}$ (%)	$\boldsymbol{QY_{c\rightarrow o}}$ (%)
**M1ap**	> 99:1	1.108×10^{-3}	42.9 ± 0.3	0.1 ± 0.1
**P1ap**	67:33	1.880×10^{-4}	59.5 ± 0.6	6.7 ± 0.1

[a] The molar absorption coefficients for the ring-open states were obtained by UV-Vis measurements. [b] PSS ratio was extracted from the ^{1}H-NMR. [c] The ones for the ring-closure states were obtained by extracting the values from the UV-Vis spectrum at the PSS, knowing the concentration of ring-open and closed species as obtained by ^{1}H-NMR.

NMR Irradiation Experiments

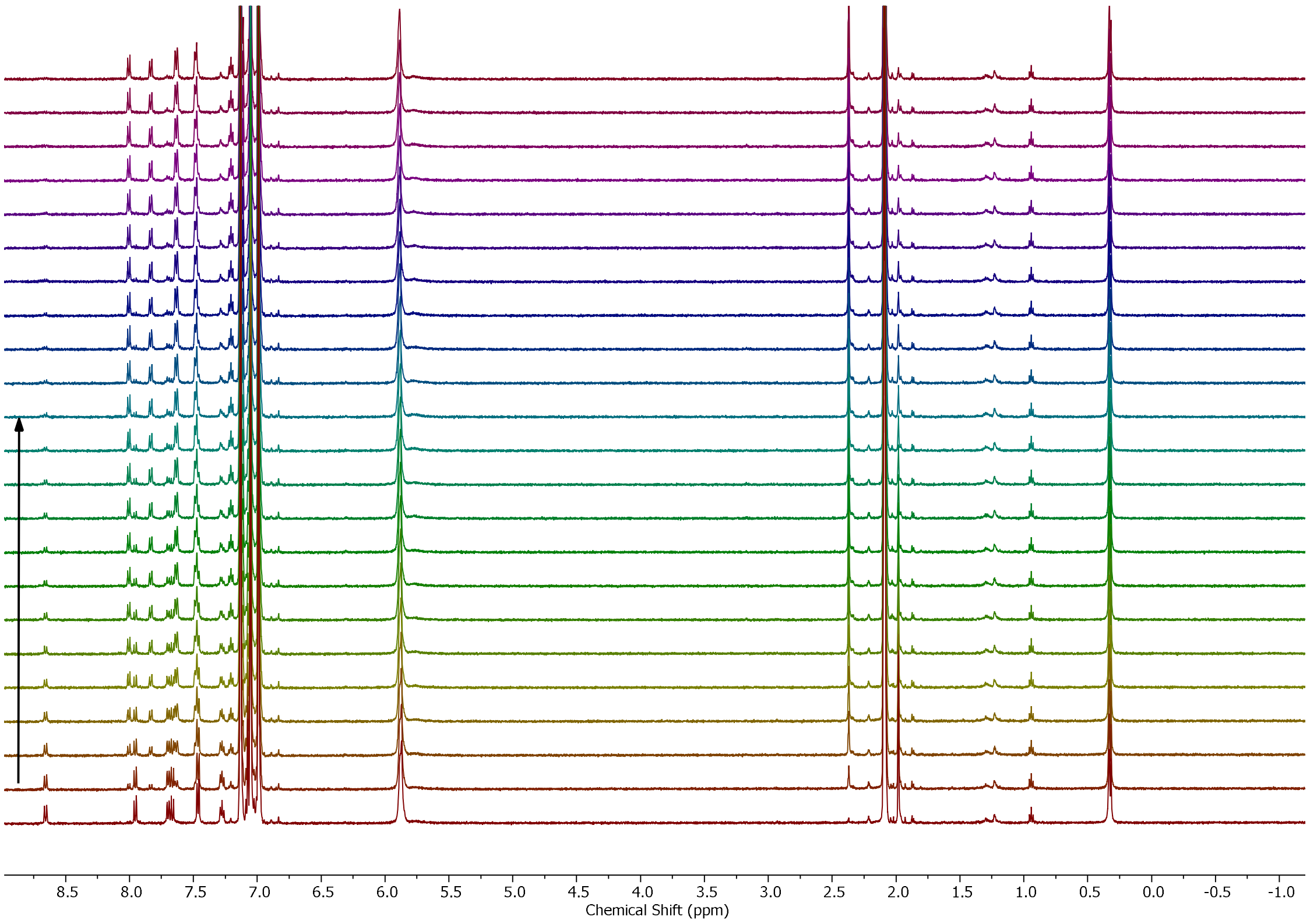

Figure S 22. Evolution of the full ^{1}H NMR spectrum of **M1ap** upon in-situ irradiation with 365 nm (500 MHz, toluene-d_8, −30 °C).

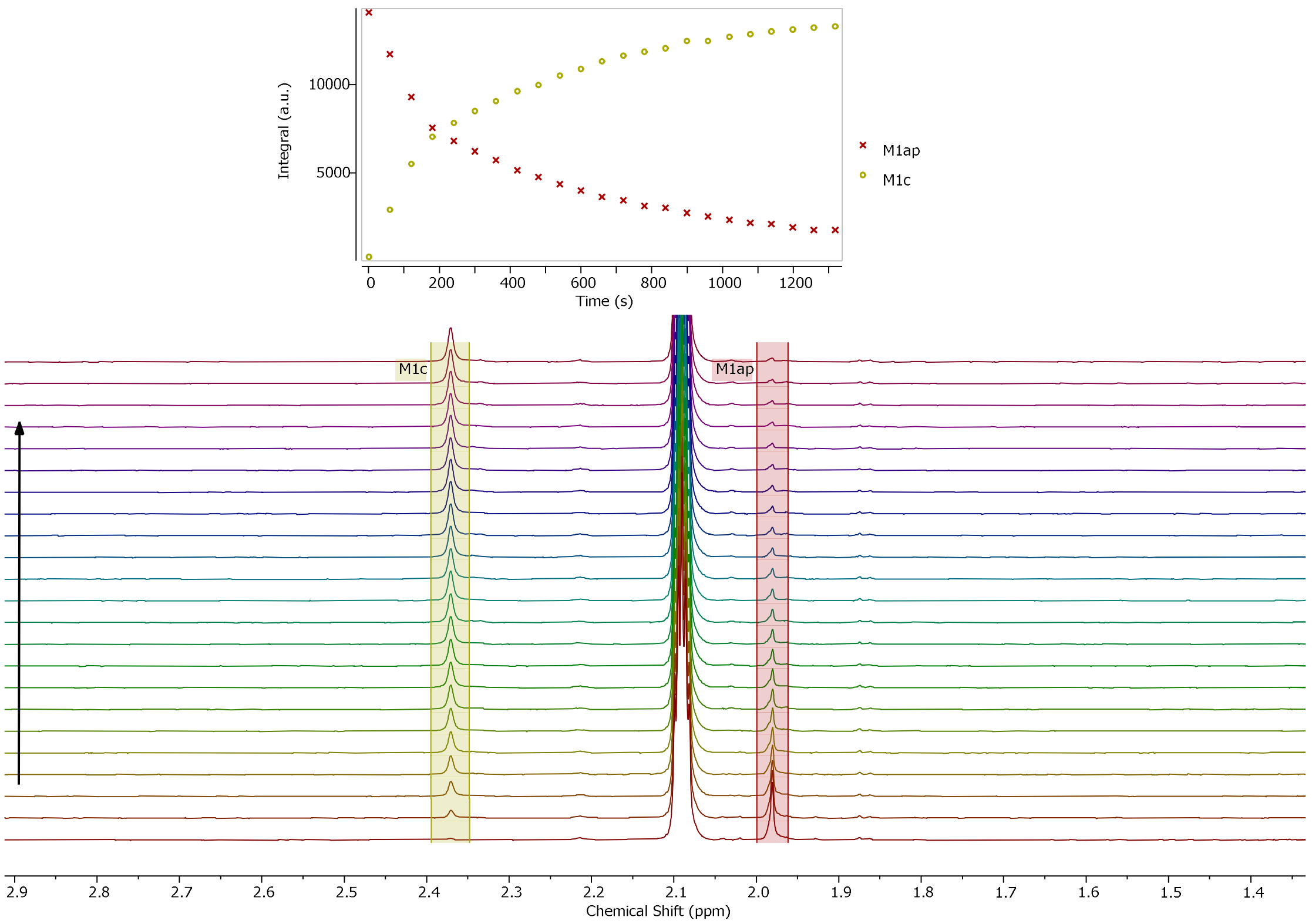

Figure S 23. Evolution of the methyl region of the ^{1}H NMR spectrum of **M1ap** upon in-situ irradiation with 365 nm and the time-profile of the integrals of **M1c** and **M1ap** (500 MHz, toluene-d_8, −30 °C).

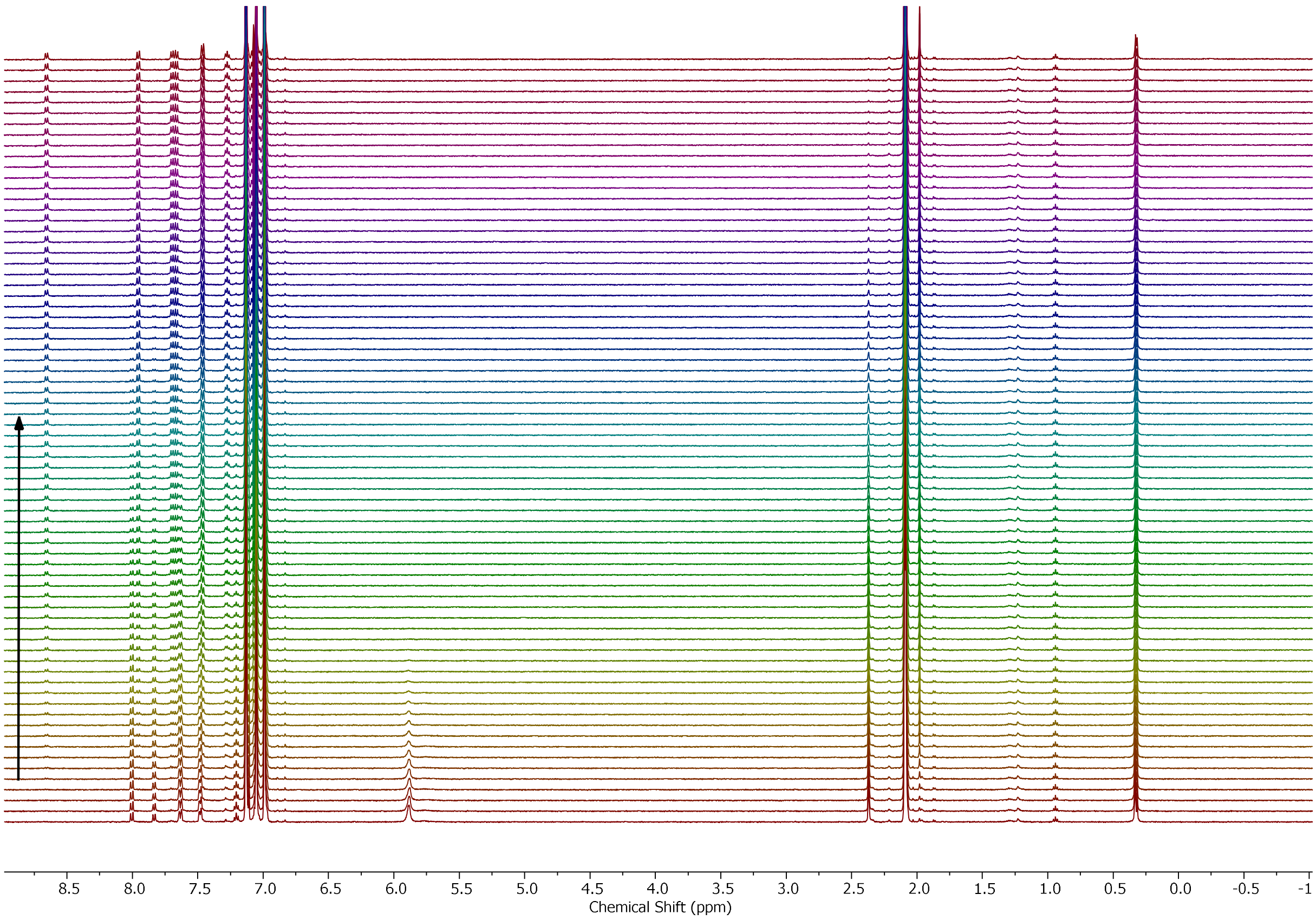

Figure S 24. Evolution of the full ^{1}H NMR spectrum of **M1c** (obtained by irradiating **M1ap** with 365 nm, Figure S22) upon in-situ irradiation with 505 nm (500 MHz, toluene-d_8, −30 °C).

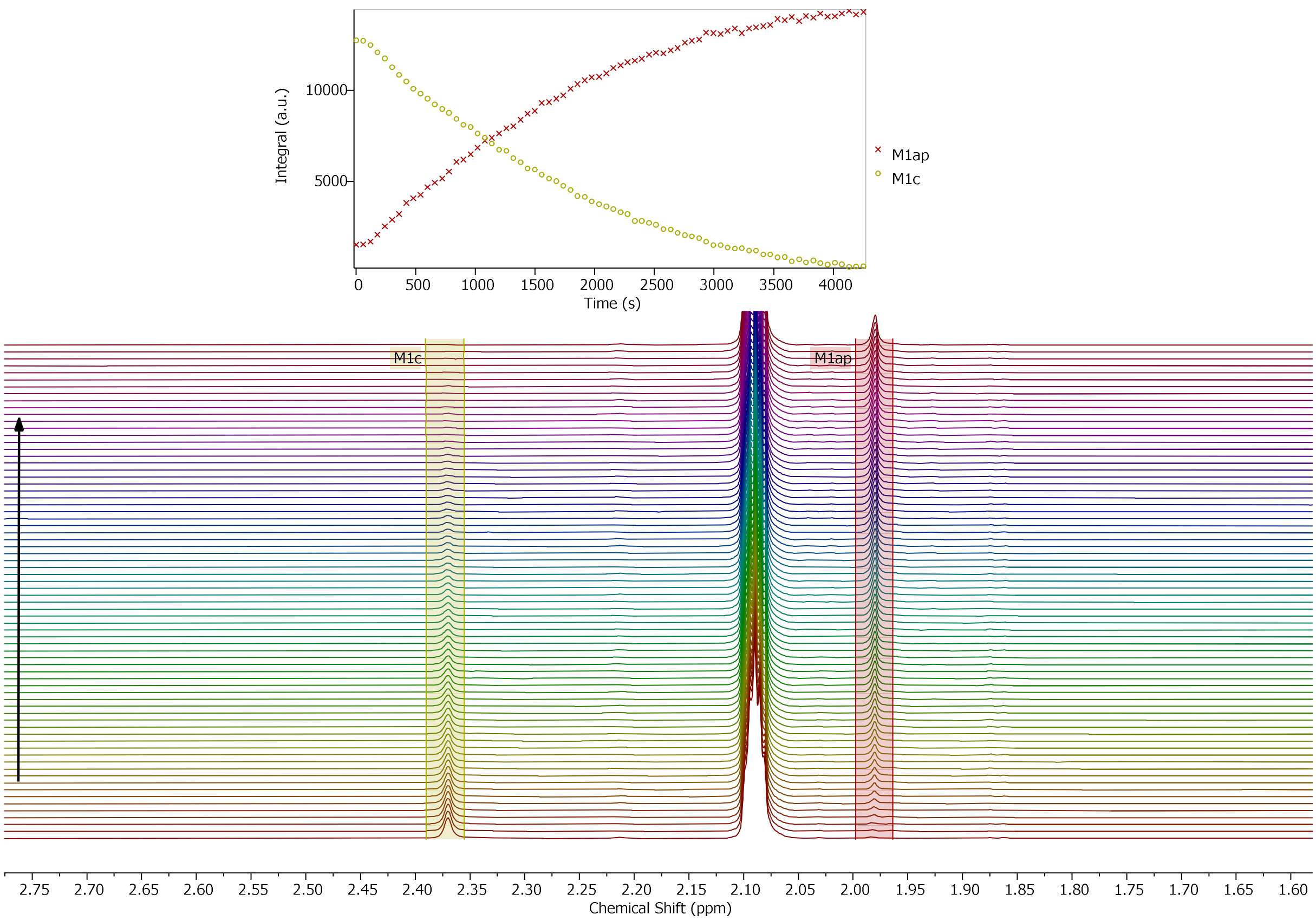

Figure S 25. Evolution of the methyl region of the ^{1}H NMR spectrum of **M1c** (obtained by irradiating **M1ap** with 365 nm, Figure S22) upon in-situ irradiation with 505 nm and the time-profile of the integrals of **M1c** and **M1ap** (500 MHz, toluene-d_8, −30 °C).

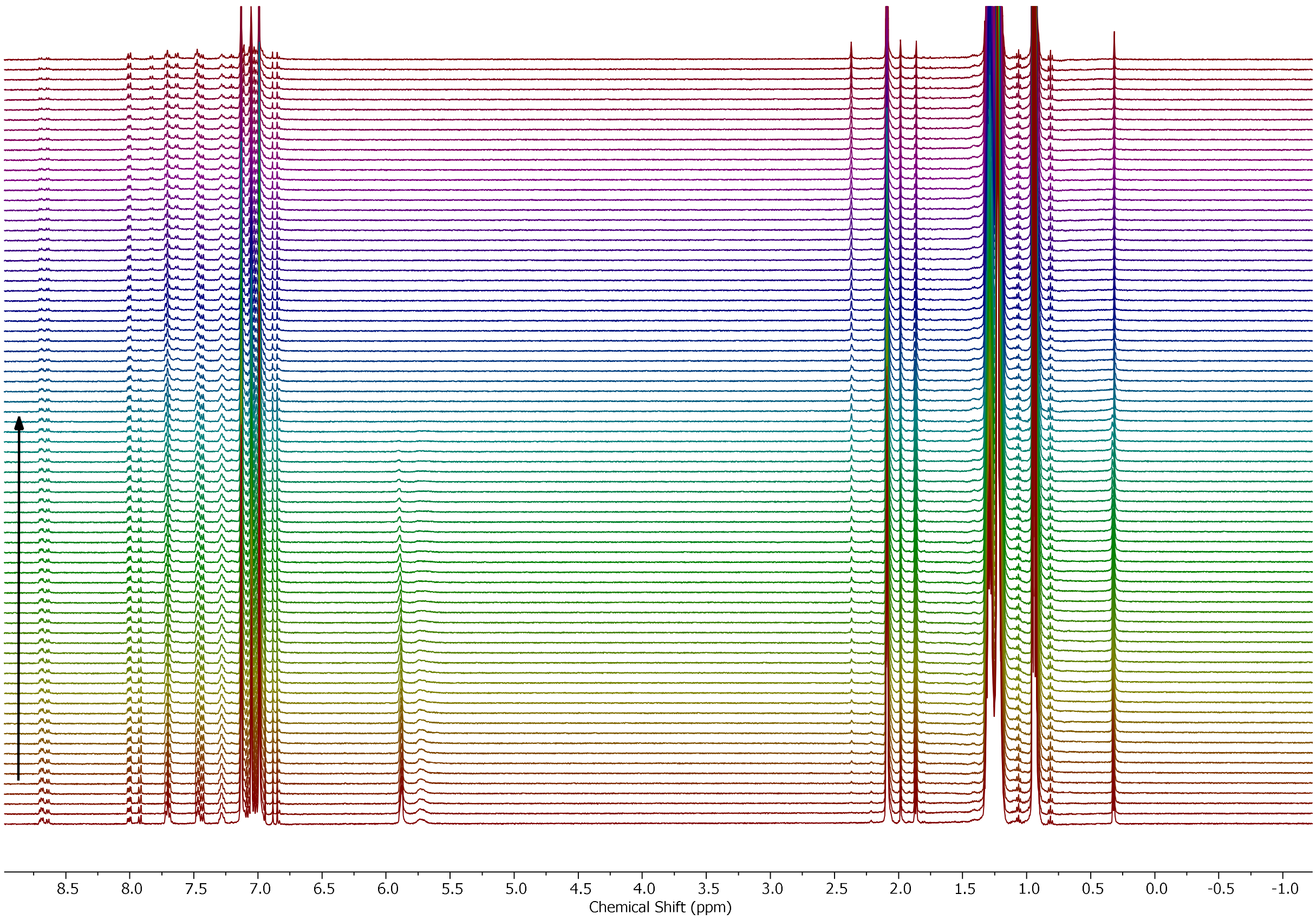

Figure S 26. Evolution of the full ^{1}H NMR spectrum of a mixture of **P1ap**/**M1p** upon in-situ irradiation with 340 and 365 nm (500 MHz, toluene-*d*$_8$, −30 °C). Irradiation wavelength was changed to 365 nm because the progress was very slow with 340 nm.

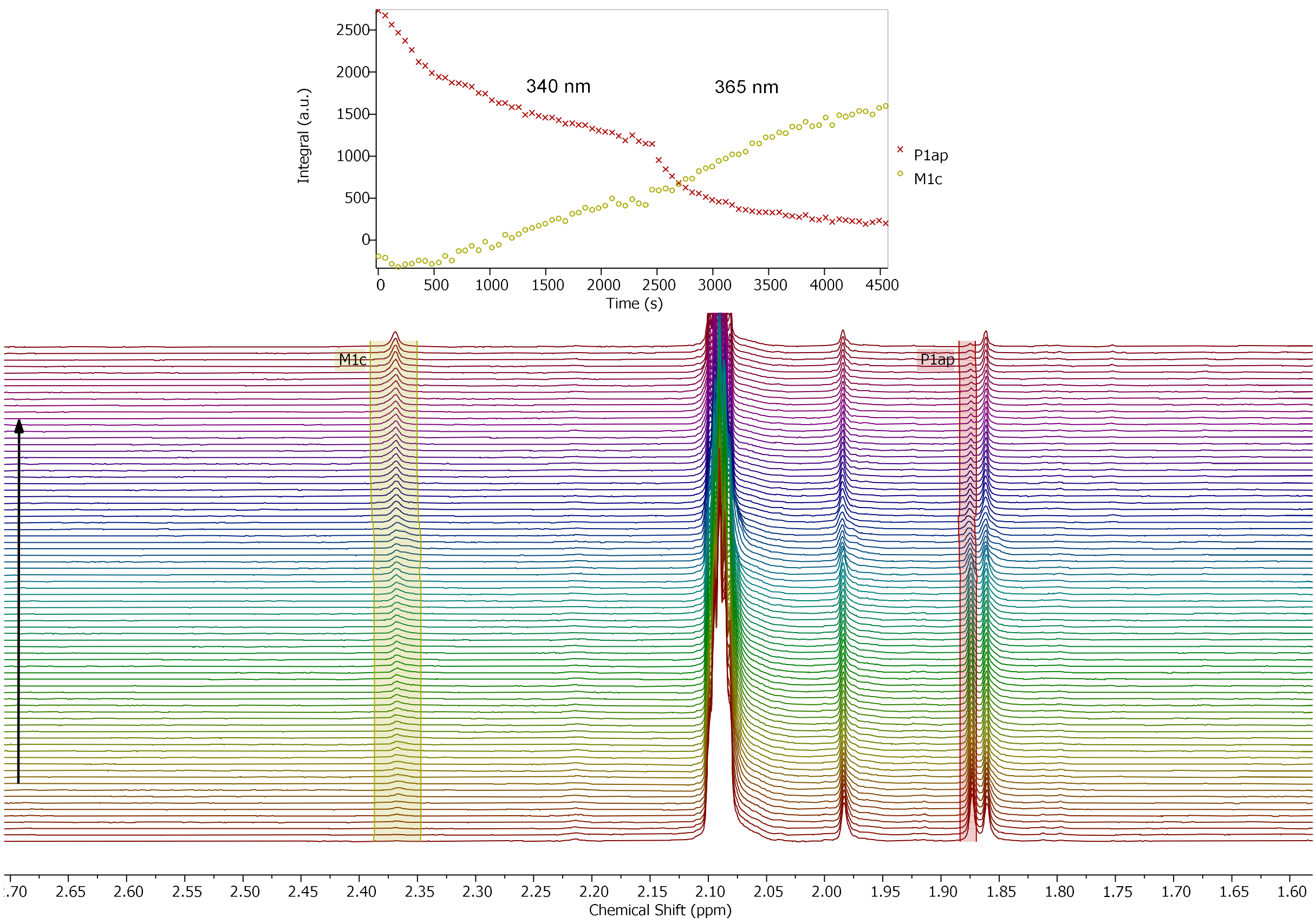

Figure S 27. Evolution of the methyl region of the ^{1}H NMR spectrum of a mixture of **P1ap**/**M1p** upon in-situ irradiation with 340 and 365 nm, and the time-profile of the integrals of **M1c** and **P1ap** (500 MHz, toluene-d_8, −30 °C). Irradiation wavelength was changed to 365 nm because the progress was very slow with 340 nm.

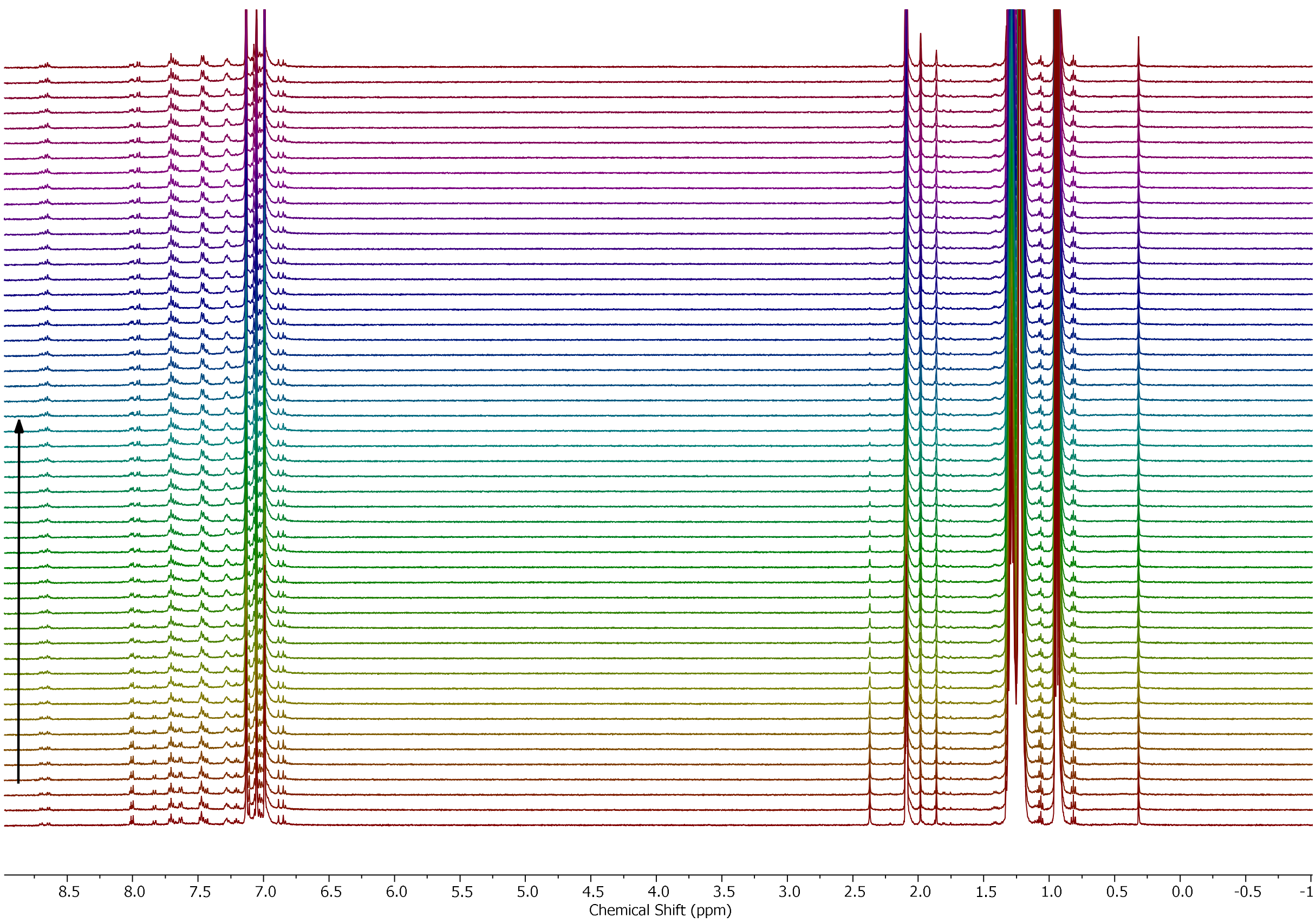

Figure S 28. Evolution of the full ^{1}H NMR spectrum of a mixture containing **M1c** (obtained by irradiating a mixture of **P1ap**/**M1p** with 340 and 365 nm, Figure S26) upon in-situ irradiation with 505 nm (500 MHz, toluene-d_8, −30 °C).

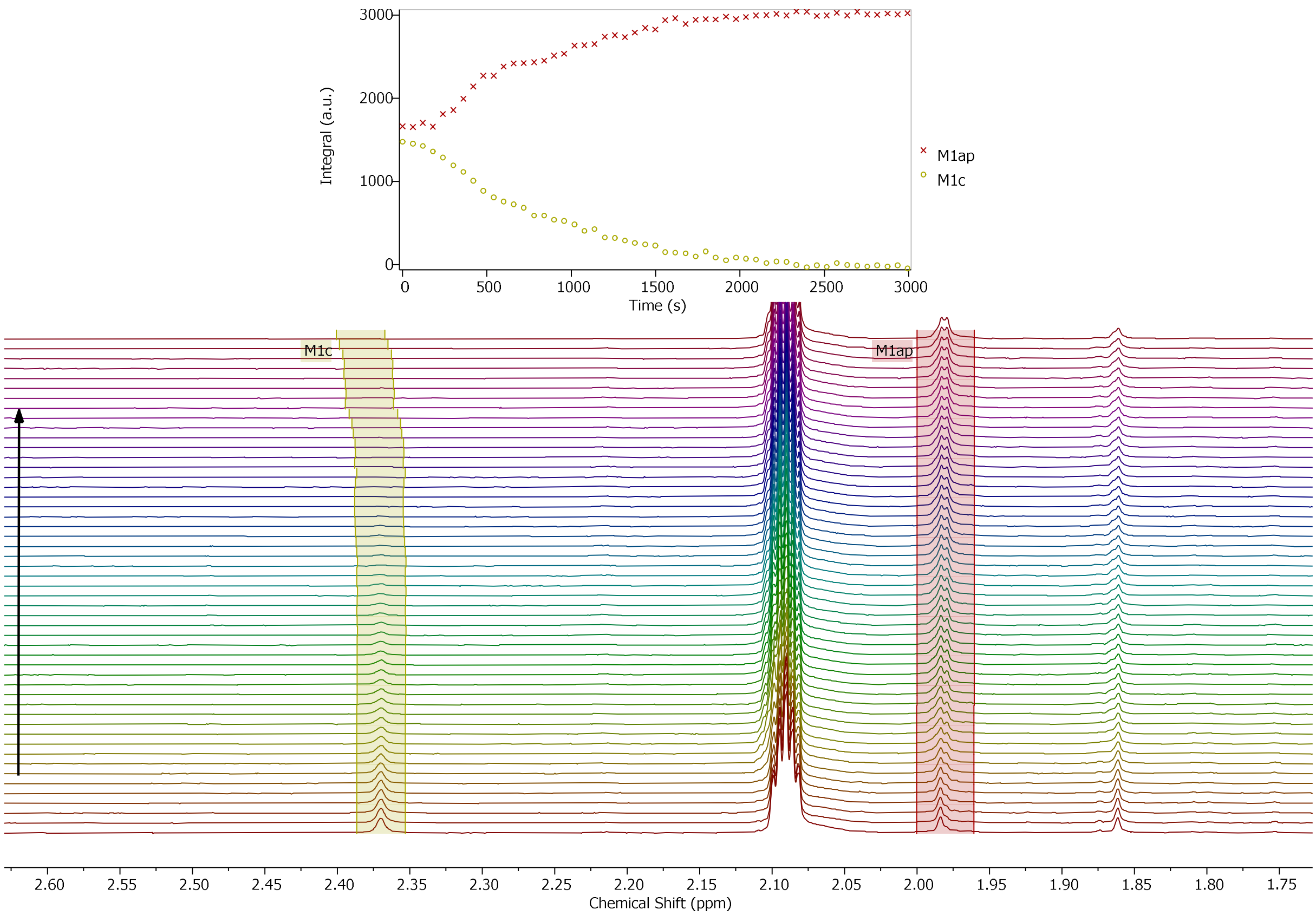

Figure S 29. Evolution of the methyl region of the ^{1}H NMR spectrum of a mixture containing **M1c** (obtained by irradiating **P1ap**/**M1p** with 340 and 365 nm, Figure S26) upon in-situ irradiation with 505 nm, and the time-profile of the integrals of **M1c** and **M1ap** (500 MHz, toluene-d_8, −30 °C).

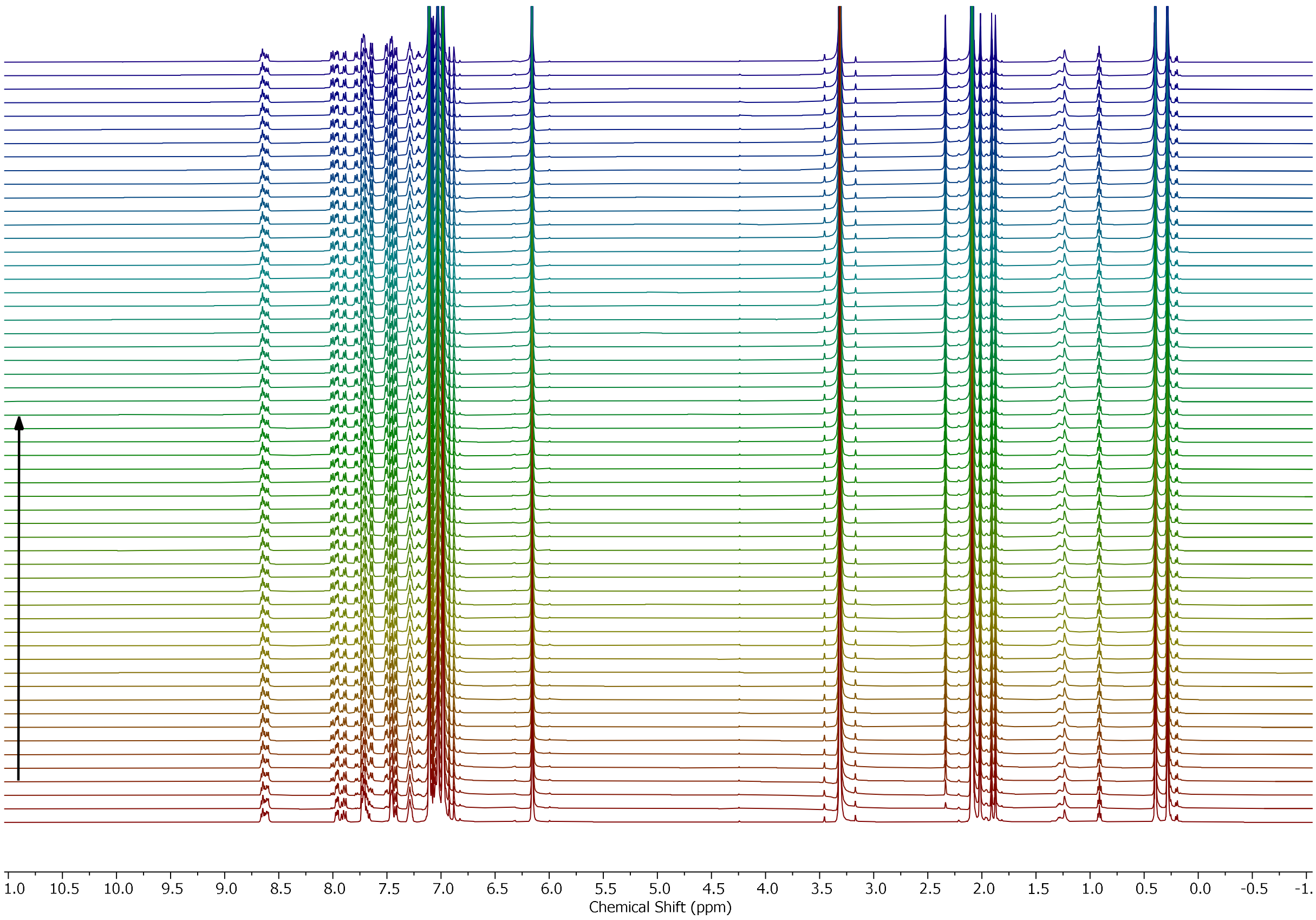

Figure S 30. Evolution of the full ^{1}H NMR spectrum of a mixture of isomers of **1** upon in-situ irradiation with 365 nm (500 MHz, toluene-d_8, 0 °C). 1,3,5-Trimethoxybenzene was used as an internal standard to check for decomposition.

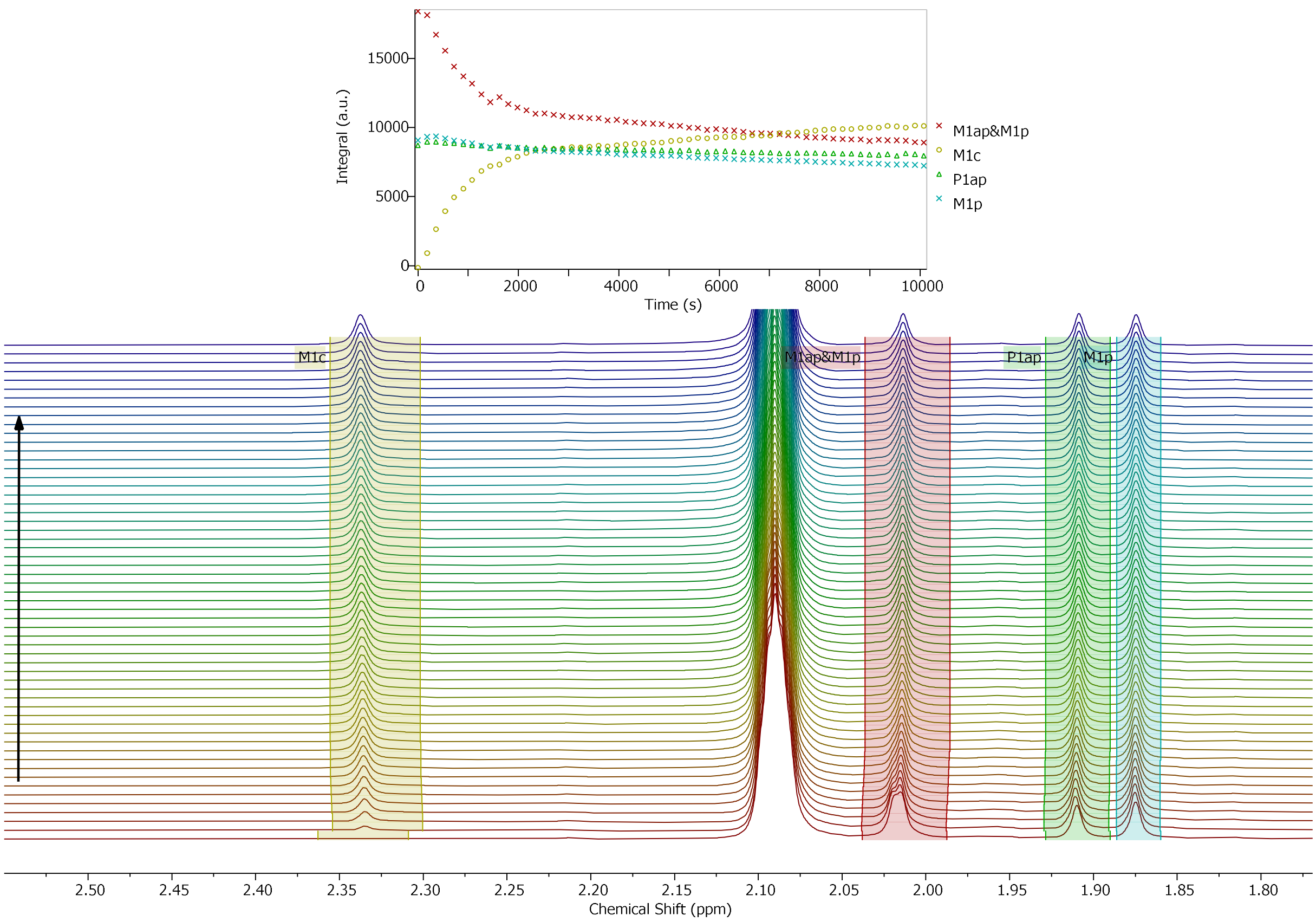

Figure S 31. Evolution of the methyl region of the ^{1}H NMR spectrum of a mixture of isomers of **1** upon in-situ irradiation with 365 nm, and the time-profile of the integrals of **M1c**, **M1ap**/**M1p**, **P1ap** and **M1p** (500 MHz, toluene-d_8, 0 °C).

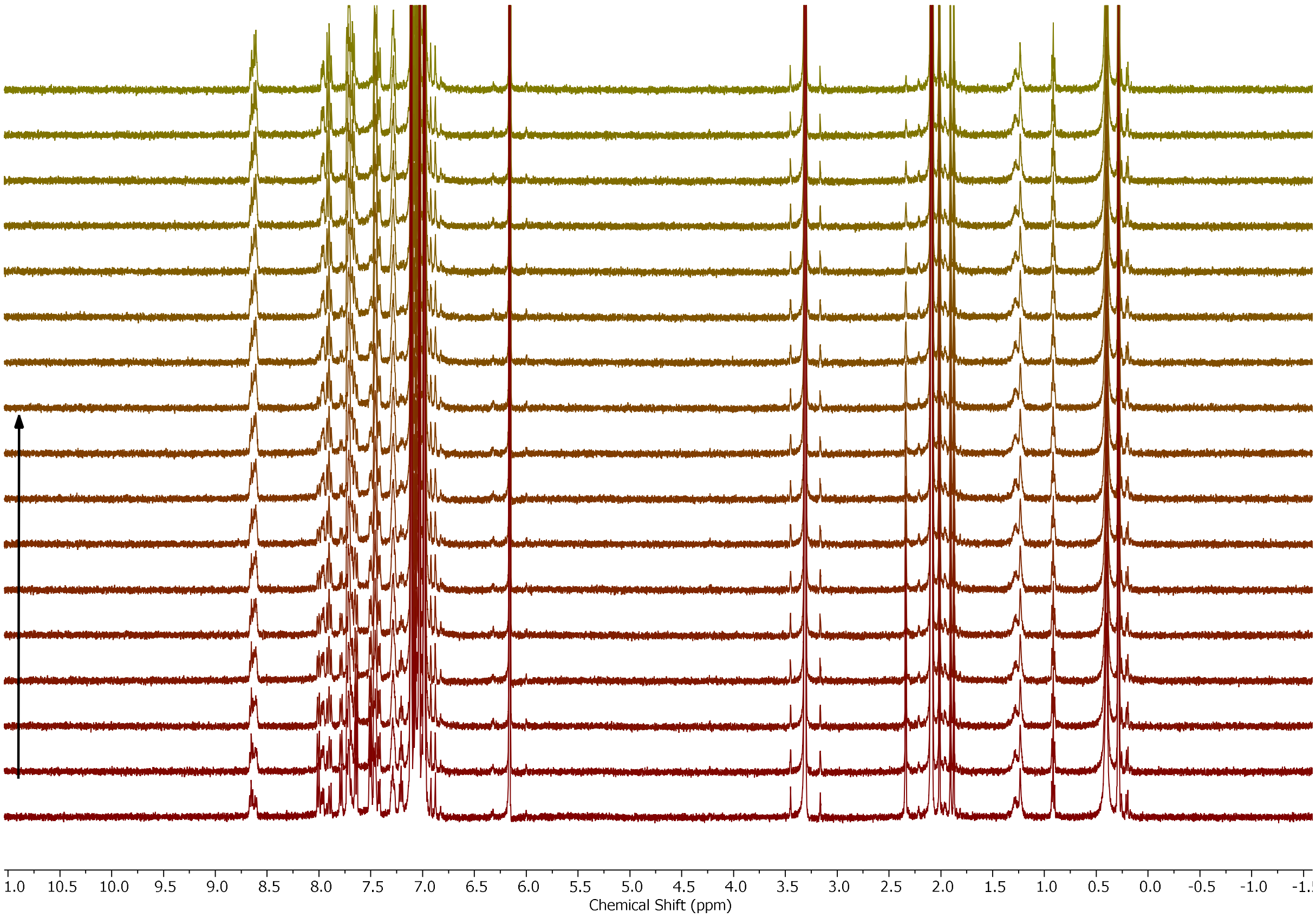

Figure S 32. Evolution of the full ^{1}H NMR spectrum of a mixture containing **M1c** (obtained by irradiating a mixture of isomers of **1** with 365 nm, Figure S30) upon in-situ irradiation with 505 nm (500 MHz, toluene-d_8, 0 °C). 1,3,5-Trimethoxybenzene was used as an internal standard to check for decomposition.

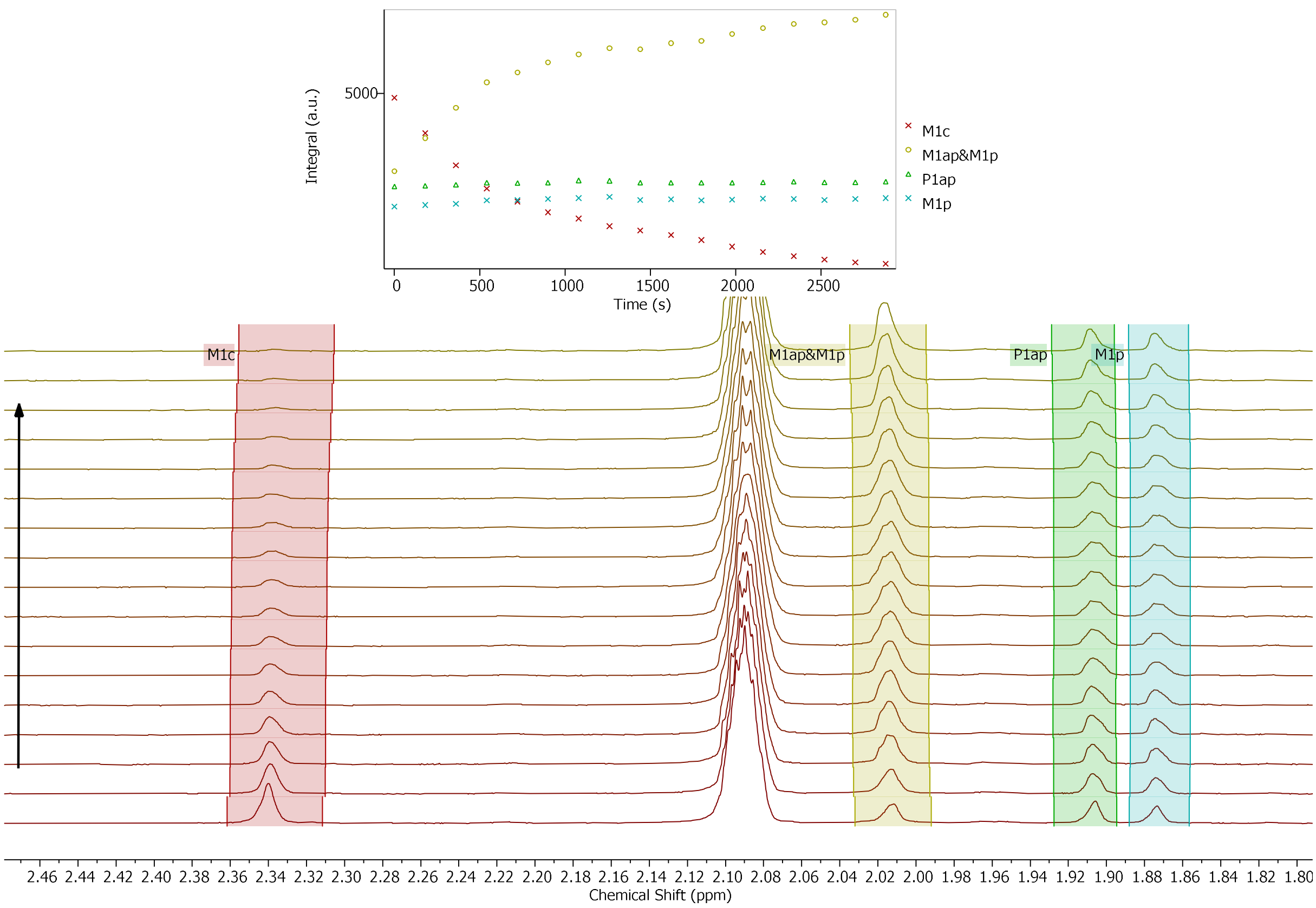

Figure S 33. Evolution of the methyl region of the ^{1}H NMR spectrum of a mixture containing **M1c** (obtained by irradiating a mixture of isomers of **1** with 365 nm, Figure S30) upon in-situ irradiation with 505 nm, and the time-profile of the integrals of **M1c**, **M1ap/M1p**, **P1ap** and **M1p** (500 MHz, toluene-d_8, 0 °C).

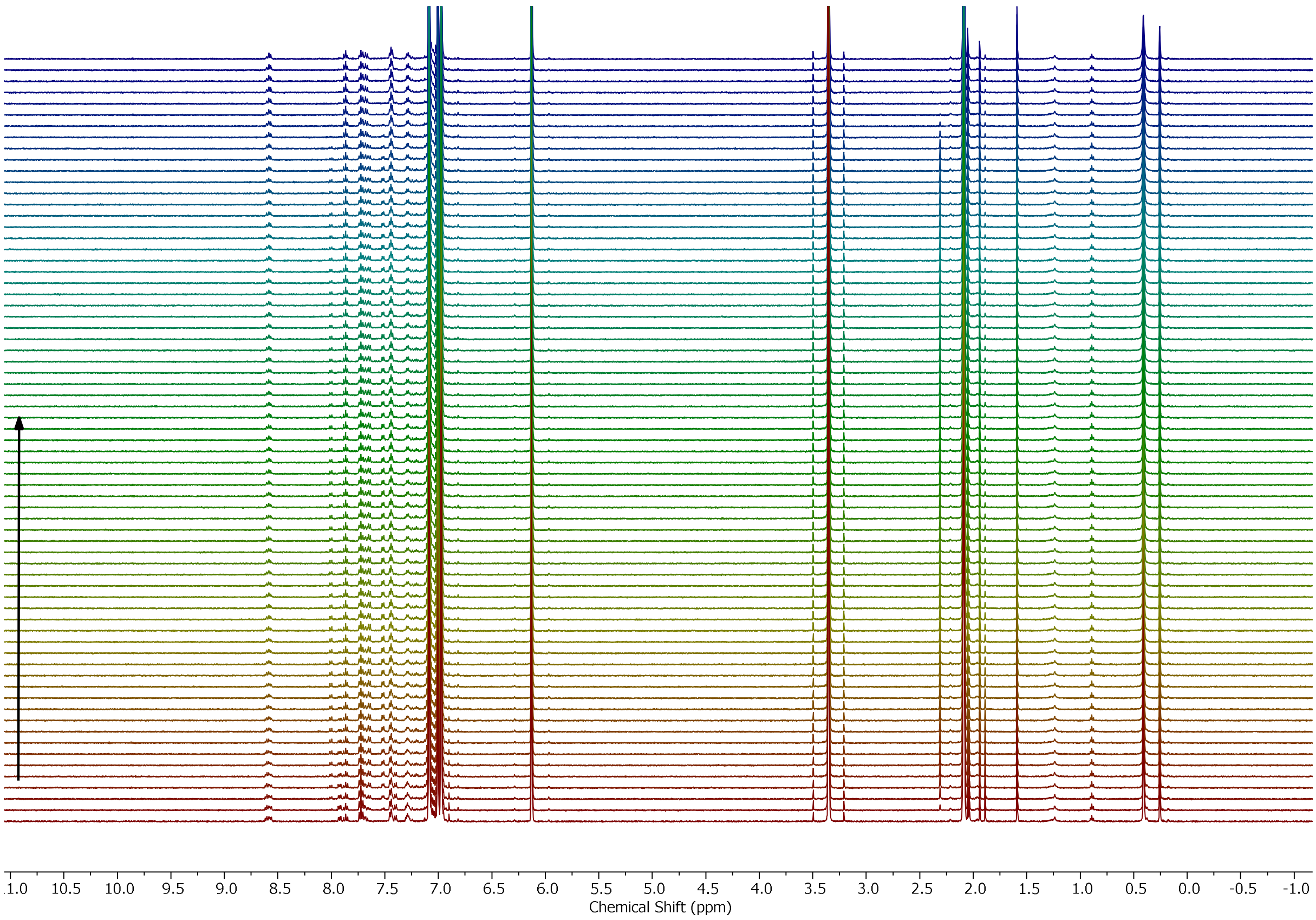

Figure S 34. Evolution of the full ^{1}H NMR spectrum of a mixture of isomers of **1**) upon in-situ irradiation with 365 nm and followed by 505 nm (500 MHz, toluene-d_8, 45 °C). 1,3,5-Trimethoxybenzene was used as an internal standard to check for decomposition.

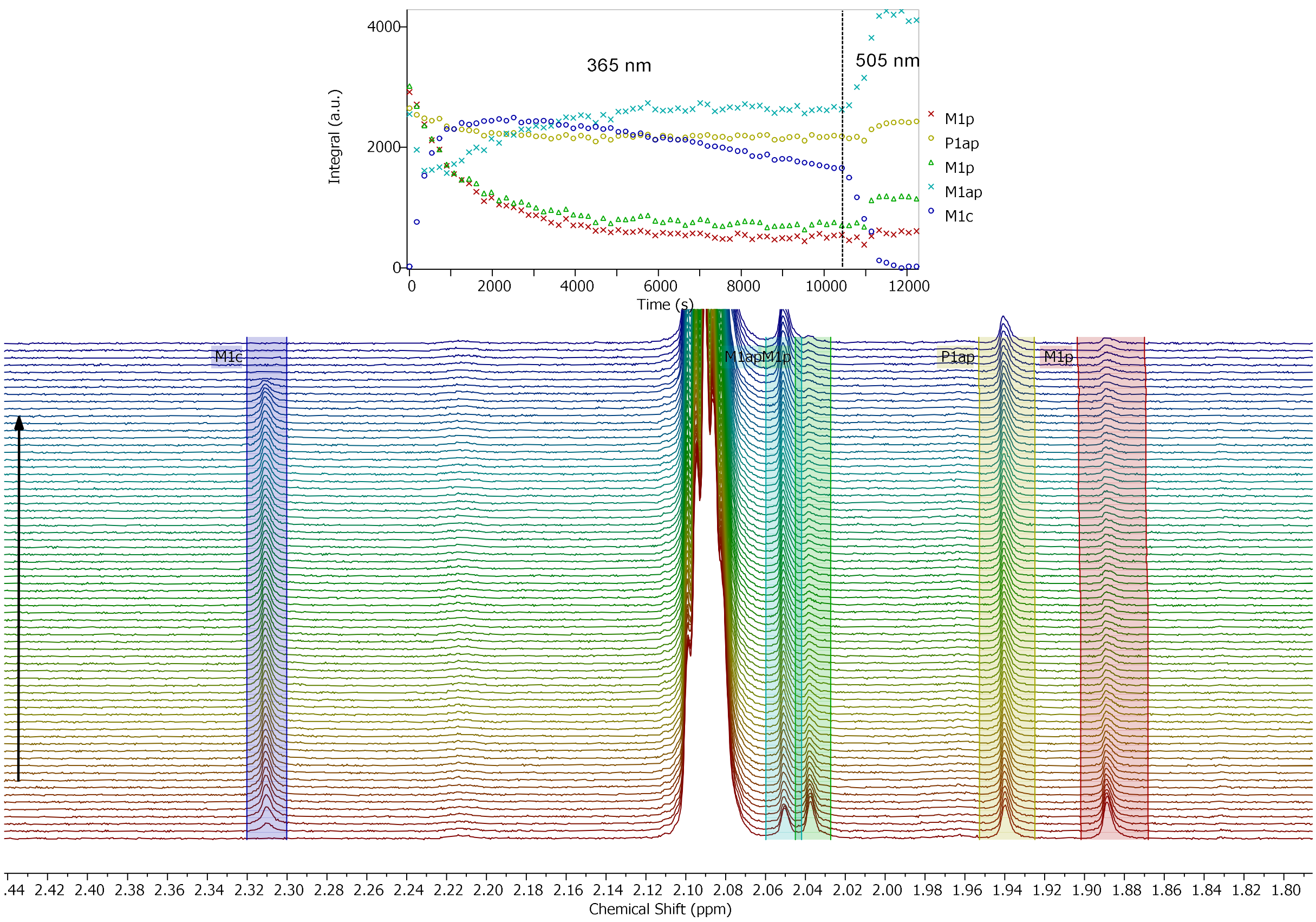

Figure S 35. Evolution of the methyl region of the ^{1}H NMR spectrum of a mixture of isomers of **1** upon in-situ irradiation with 365 nm followed by 505 nm, and the time-profile of the integrals of **M1c**, **M1ap**/**M1p**, **P1ap** and **M1p** (500 MHz, toluene-d_8, 45 °C).

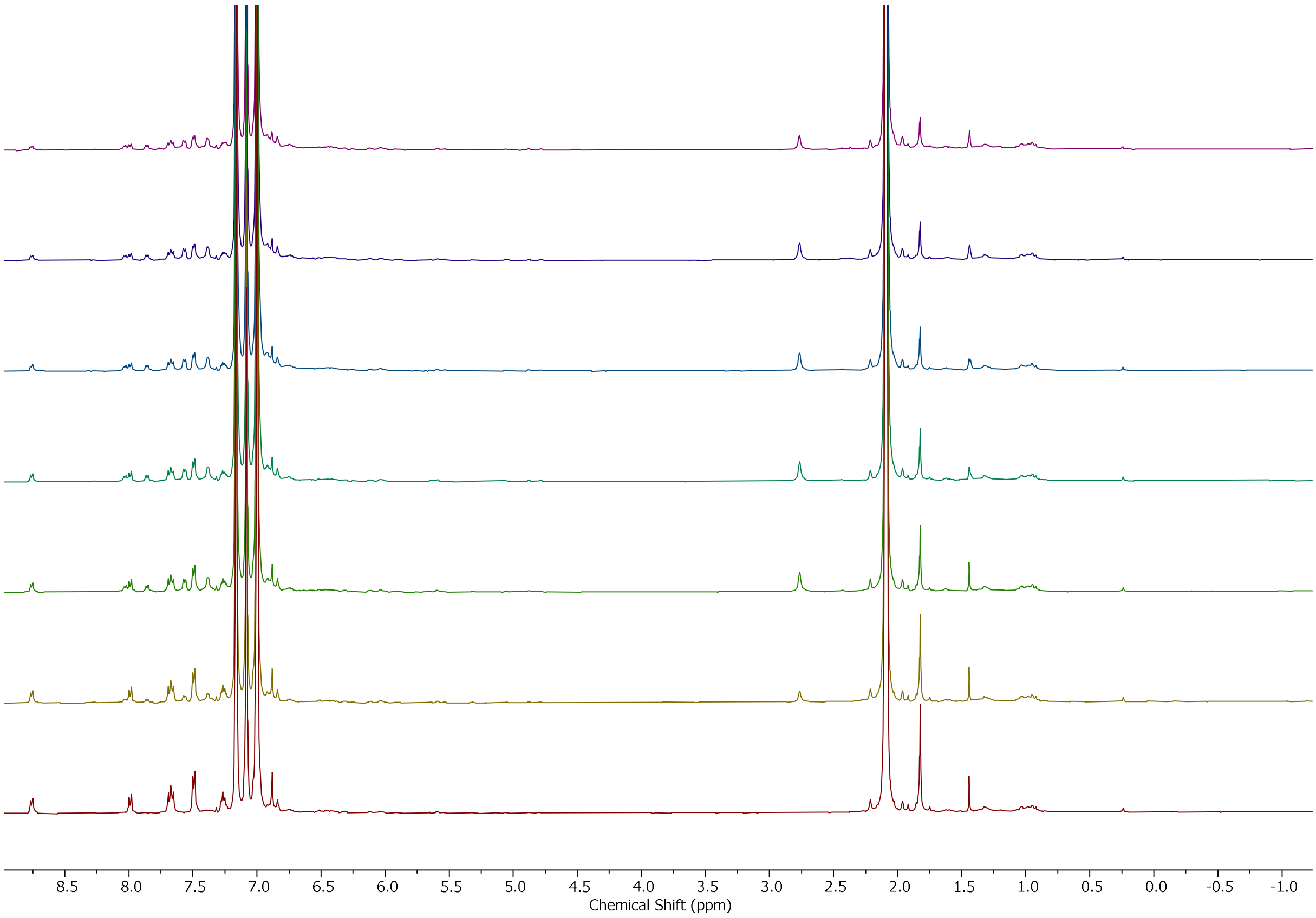

Figure S 36. Evolution of the full ^{1}H NMR spectrum of **P1ap** upon in-situ irradiation with 365 nm (500 MHz, toluene-*d*$_8$, −80 °C).

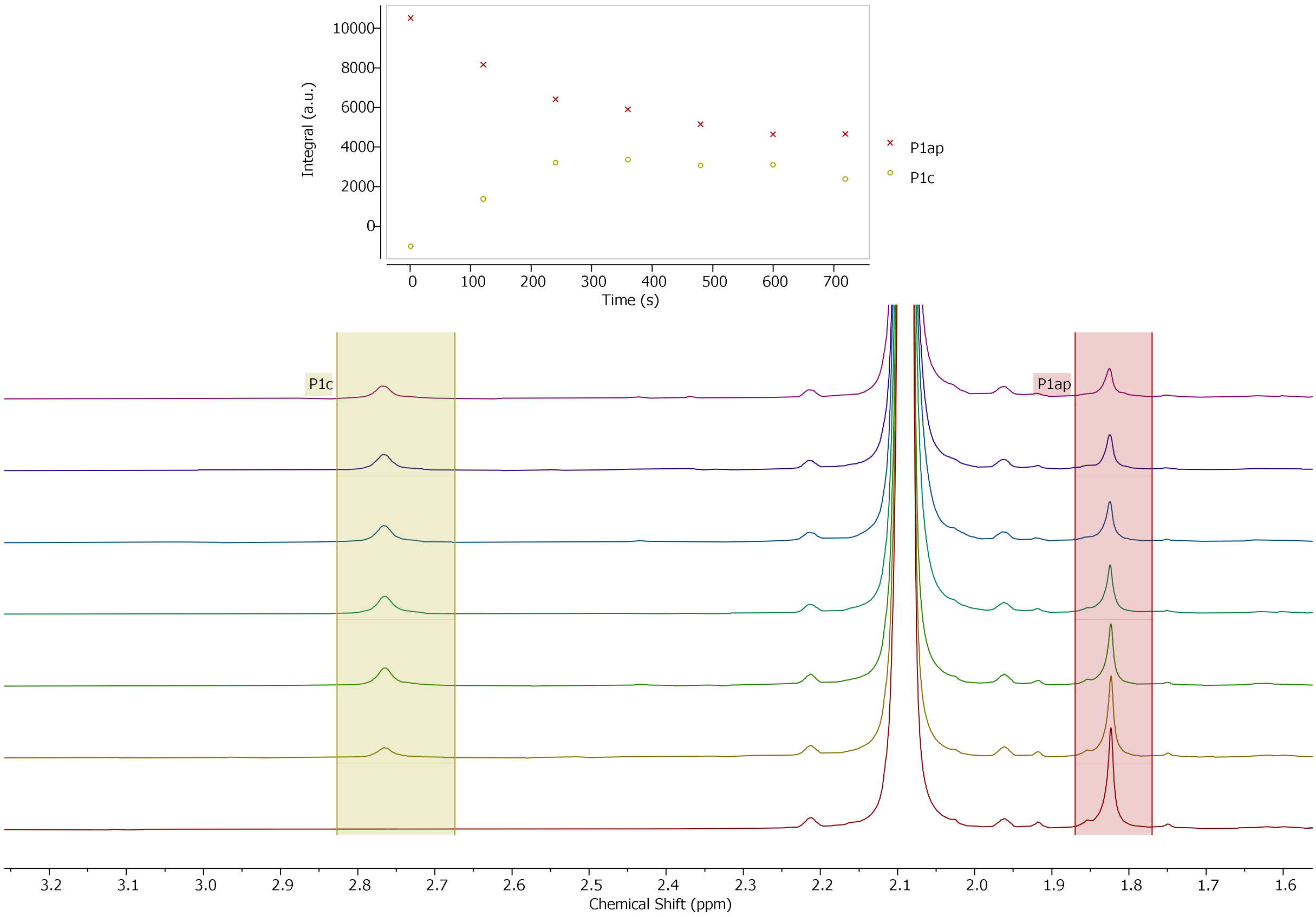

Figure S 37. Evolution of the methyl region of the ^{1}H NMR spectrum of **P1ap** upon in-situ irradiation with 365 nm, and the time-profile of the integrals of **P1c** and **P1ap** (500 MHz, toluene-d_8, −80 °C).

Computational Analysis

Ground & Transition State Calculations

The ground state geometry optimizations of **M1ap**, **P1ap**, **M1c** and **P1c** were performed using DFT calculations at the r^2SCAN-3c/CPCM(toluene)[8,9] level of theory (Figure S38). The energy minima were confirmed upon subsequent frequency calculations. The energies of the optimized geometries were refined at the ωB97X-D4/def2-TZVPP/CPCM(toluene)[10,11] level of theory via single-point energy calculations (Figure S39).

Transition states of the helix inversion in the open (antiparallel, **TSap**) and closed (**TSc**) form were calculated using the Nudged Elastic Band with Transition State optimization (NEB-TS) included in the ORCA package.[1] Subsequently, the transition state geometries were optimized at the r^2SCAN-3c/CPCM(toluene) level of theory (Figure S38). The energy maxima of the transition states were confirmed by subsequent frequency calculations, verifying the presence of a single imaginary harmonic frequency. The energies of the optimized transition states were refined at the ωB97X-D4/def2-TZVPP/CPCM(toluene) level of theory via single-point energy calculations (Figure S39).

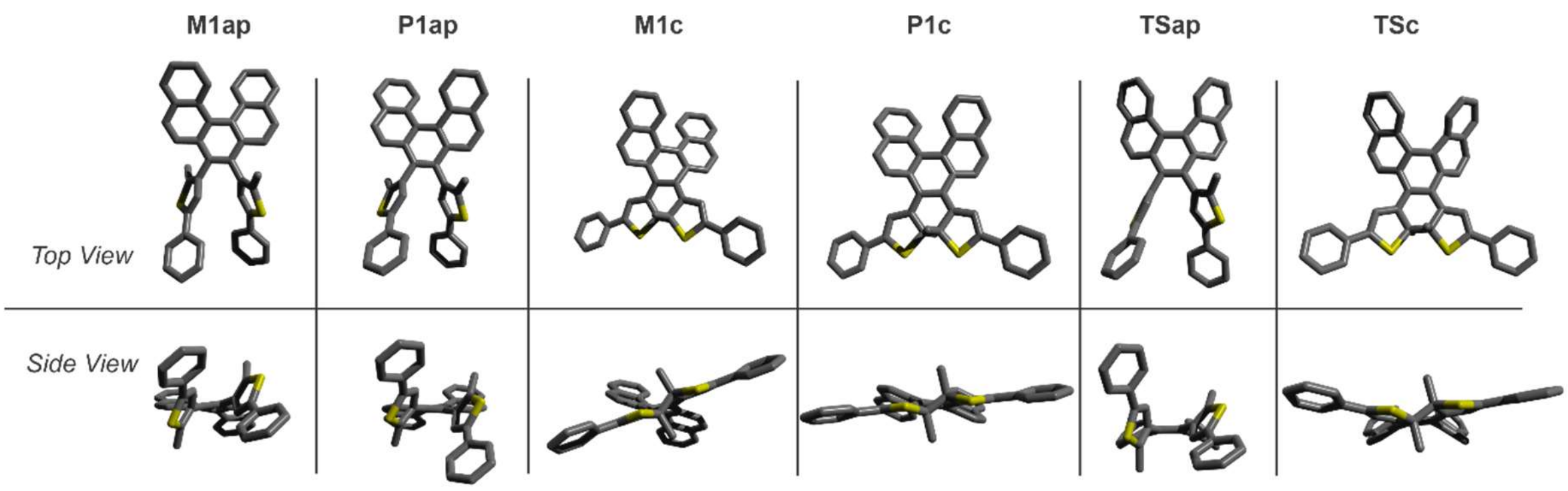

Figure S 38. Top and side views of the DFT-optimized lowest energy structures of **M1ap**, **P1ap**, **M1c**, **P1c**, **TSap** and **TSc**.

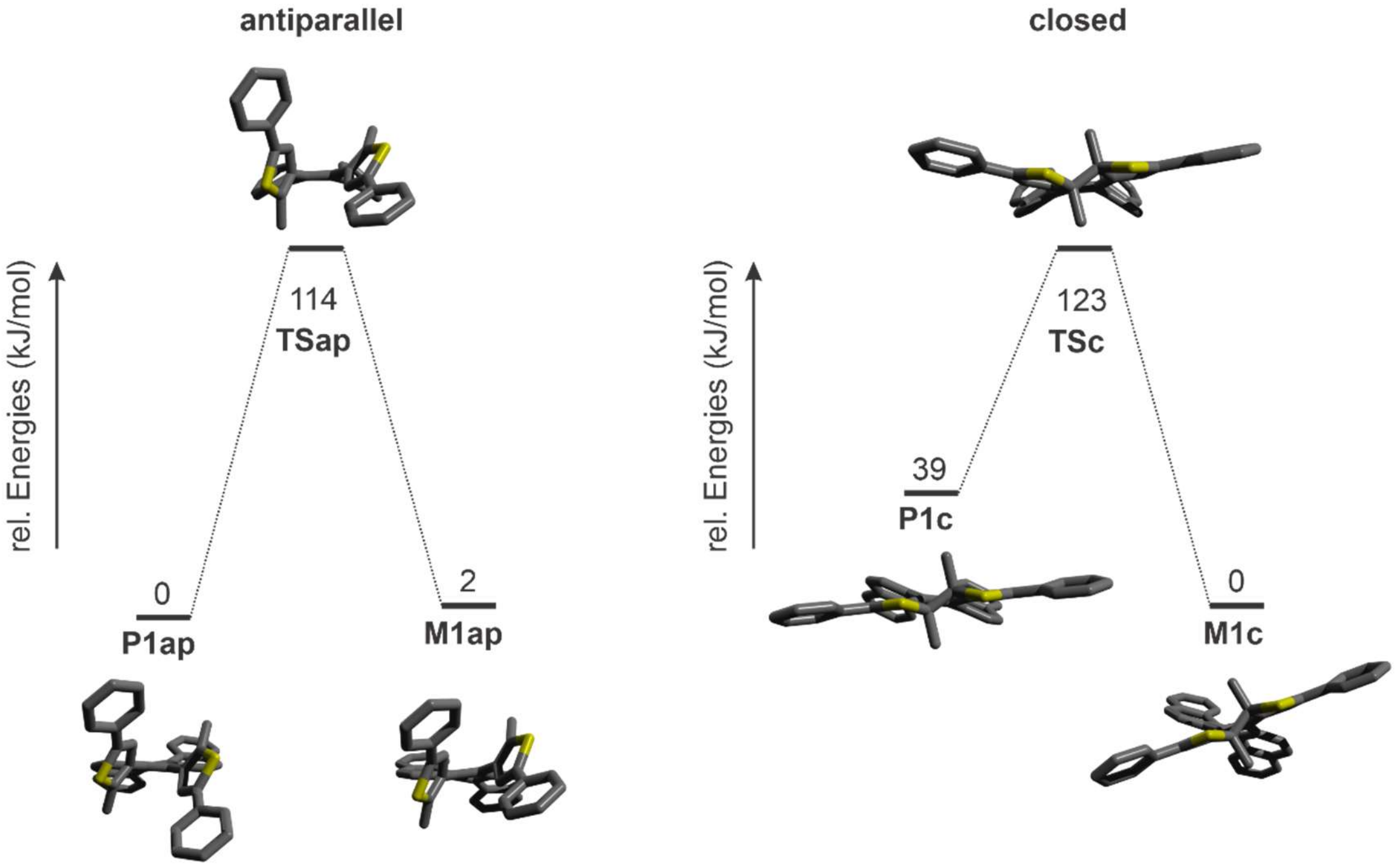

Figure S 39. Relative energy diagram of **P1ap**, **M1ap**, **TSap** (left), and **P1c**, **M1c**, **TSc** (right).

Conformational Analysis

A conformational analysis of **M1ap**, **P1ap**, **M1c** and **P1c** was performed using CREST[12]. Significantly contributing conformers were reoptimized at the r^{2}SCAN-3c/CPCM(toluene) level of theory and the energy minima were confirmed with subsequent frequency calculations. The energies of the optimized conformers were refined at the ωB97X-D4/def2-TZVPP/CPCM(toluene) level of theory via single-point energy calculations (Figure S40). These energies were then used for a subsequent Boltzmann analysis, which is summarized in Table S2.

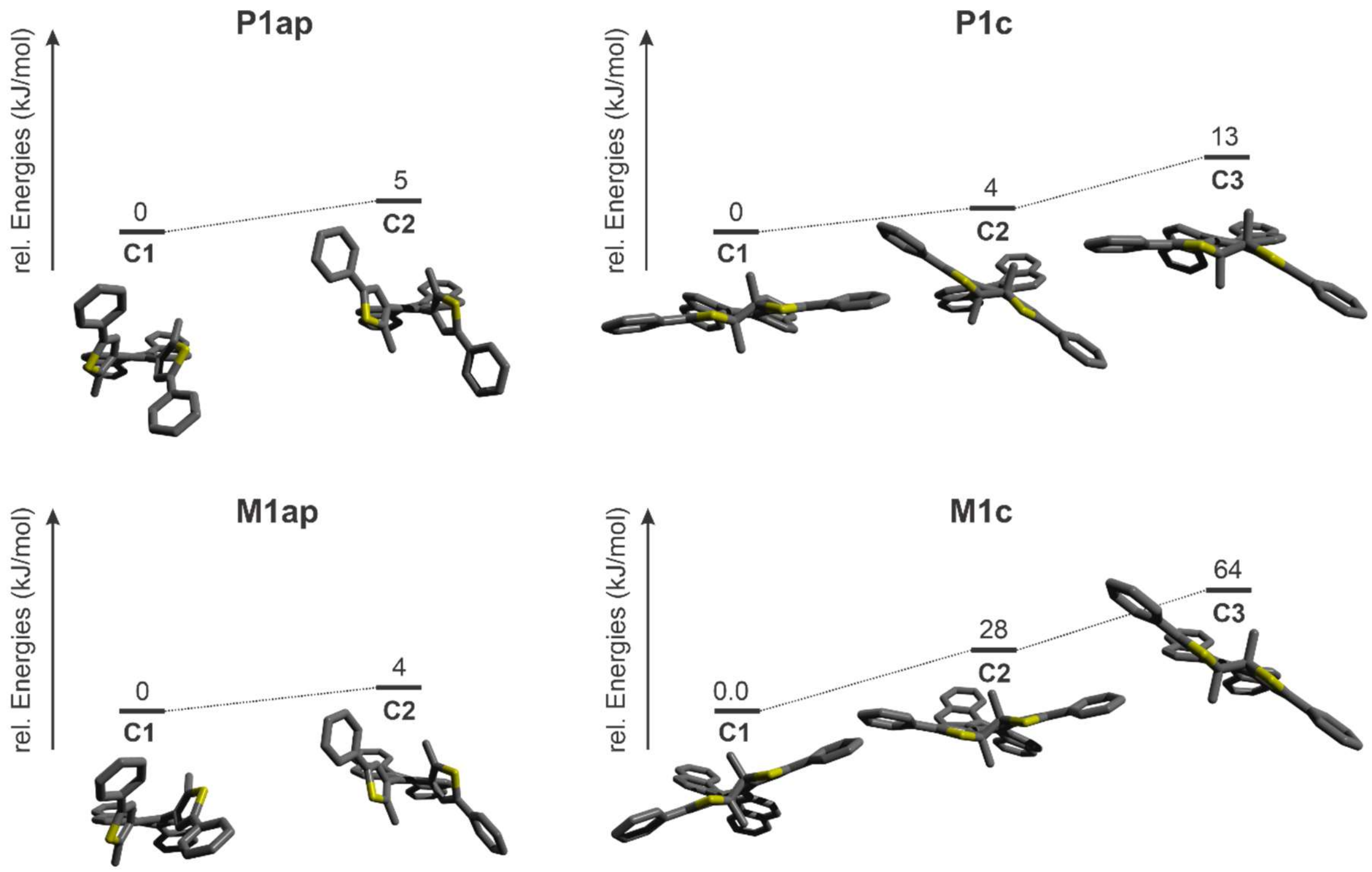

Figure S 40. Conformational analysis of **P1ap**, **P1c**, **M1ap** and **M1c**.

Table S 2. Summary of the energies (ωB97X-D4/def2-TZVPP/CPCM(toluene)) of the conformational analysis and the respective Boltzmann population at 20°C for **P1ap**, **M1ap** and **P1c**. For **M1c**, no Boltzmann analysis was performed because the conformers C2 and C3 were significantly higher in energy.

**P1ap**		
Conformer	Energy	Boltzmann population (20°C)
C1	-2492.148012 E_h	87.2%
C2	-2492.146228 E_h	12.8%
Boltzmann-averaged energy: -2492.147784 E_h<br>Boltzmann-averaged relative energy (vs. C1 = 0 kJ/mol): 0.6 kJ/mol		

**M1ap**		
Conformer	Energy	Boltzmann population (20°C)
C1	-2492.147372 E_h	81.5%
C2	-2492.145993 E_h	18.5%
Boltzmann-averaged energy: -2492.147117 E_h<br>Boltzmann-averaged relative energy (vs. C1 = 0 kJ/mol): 0.7 kJ/mol		

**P1c**		
Conformer	Energy	Boltzmann population (20°C)
C1	-2492.093712 E_h	83.8%
C2	-2492.092159 E_h	15.7%
C3	-2492.088904 E_h	0.5%
Boltzmann-averaged energy: -2492.093446 E_h<br>Boltzmann-averaged relative energy (vs. C1 = 0 kJ/mol): 0.7 kJ/mol		

NMR Shift Calculations

Table S 3. The isotropic shielding tensors σ of all conformers of **P1ap** and **M1ap**, as well as the Boltzmann-weighted tensors, calculated at the ωB97X-D4/def2-TZVPP/CPCM(toluene) level of theory, using the GIAO method.

**P1ap**			
	Average isotropic shielding tensors of both thiophene protons (σ)	Average isotropic shielding tensors of all CH_3 protons (σ)	Boltzmann-population (20°C)
C1	24.53	29.32	87.2%
C2	23.91	29.47	12.8%
**Boltzmann-weighted tensor**	**24.45**	**29.34**	

**M1ap**			
	Average isotropic shielding tensors of both thiophene protons (σ)	Average isotropic shielding tensors of all CH_3 protons (σ)	Boltzmann-population (20°C)
C1	24.59	29.23	81.5%
C2	23.91	29.40	18.5%
**Boltzmann-weighted tensor**	**24.46**	**29.26**	

TDDFT Calculations

Full TDDFT calculations of the ground states of **M1ap**, **P1ap**, **M1c**, and **P1c** were performed at the ωB97X-D4/def2-TZVPP/CPCM(toluene) level of theory, considering 30 singlet transitions. The calculated CD spectra (with an average shift of 40 nm) match well with the measured spectra, allowing the assignment of the absolute configuration (Figure S41-44).

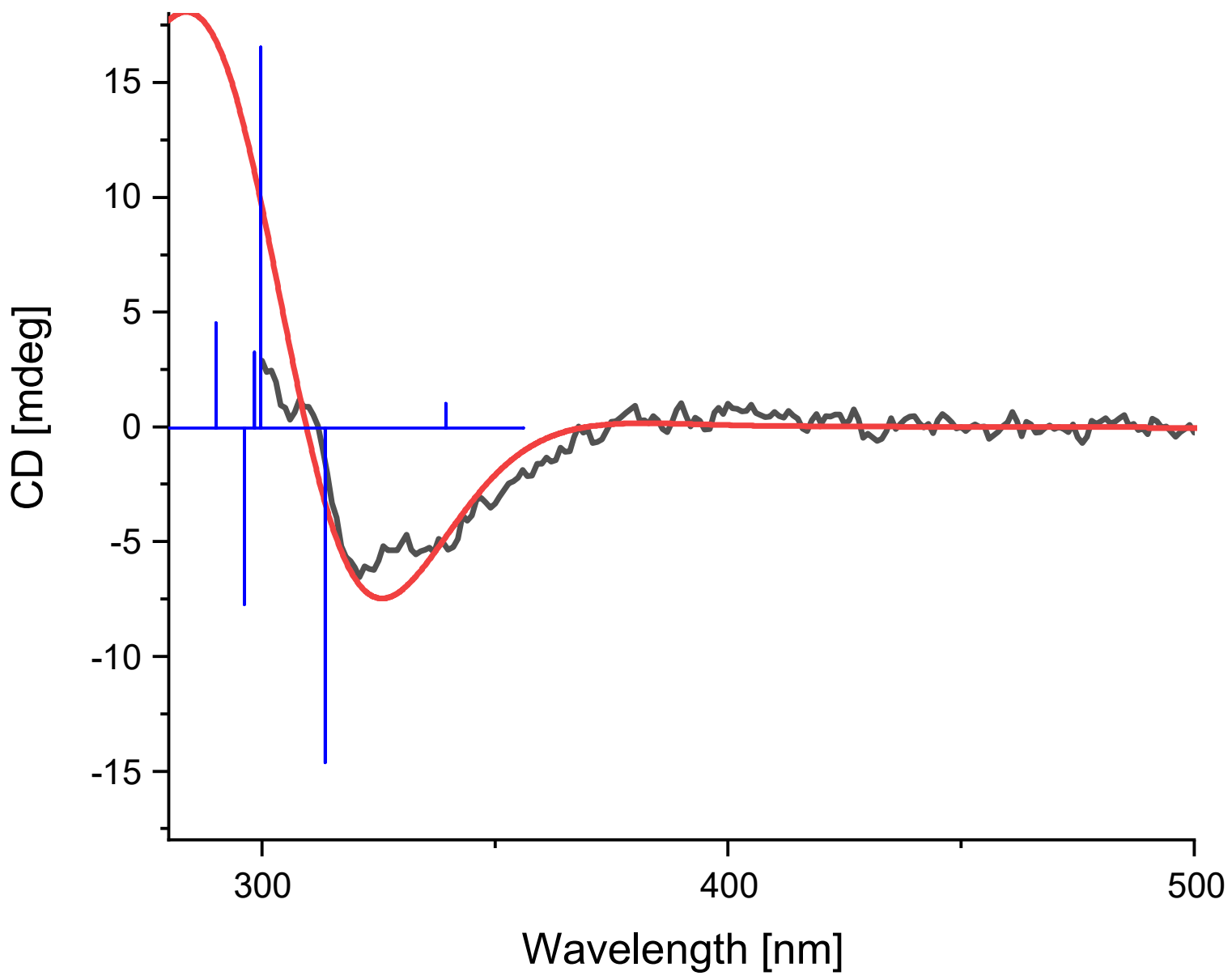

Figure S 41. Calculated CD spectrum of (R_a,R_a)-(M)-**1ap** (red), the calculated singlet transitions (blue) and the measured experimental spectrum of the first eluting peak of the HPLC (Figure S9).

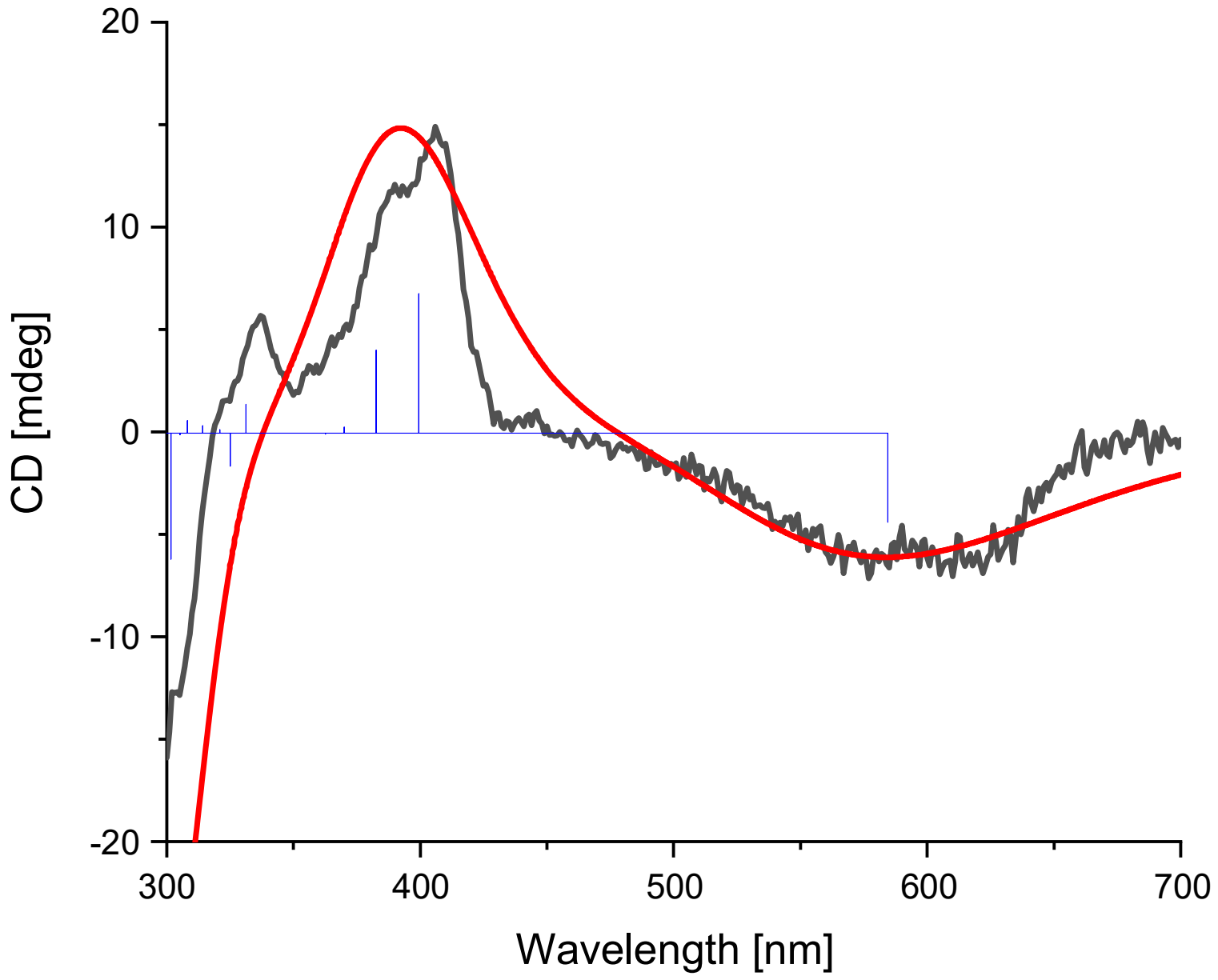

Figure S 42. Calculated CD spectrum of (S,S)-(M)-**1c** (red), the calculated singlet transitions (blue) and the measured experimental spectrum of the ring-closed species obtained by irradiating the first eluting peak of the HPLC at 20 °C (Figure S9).

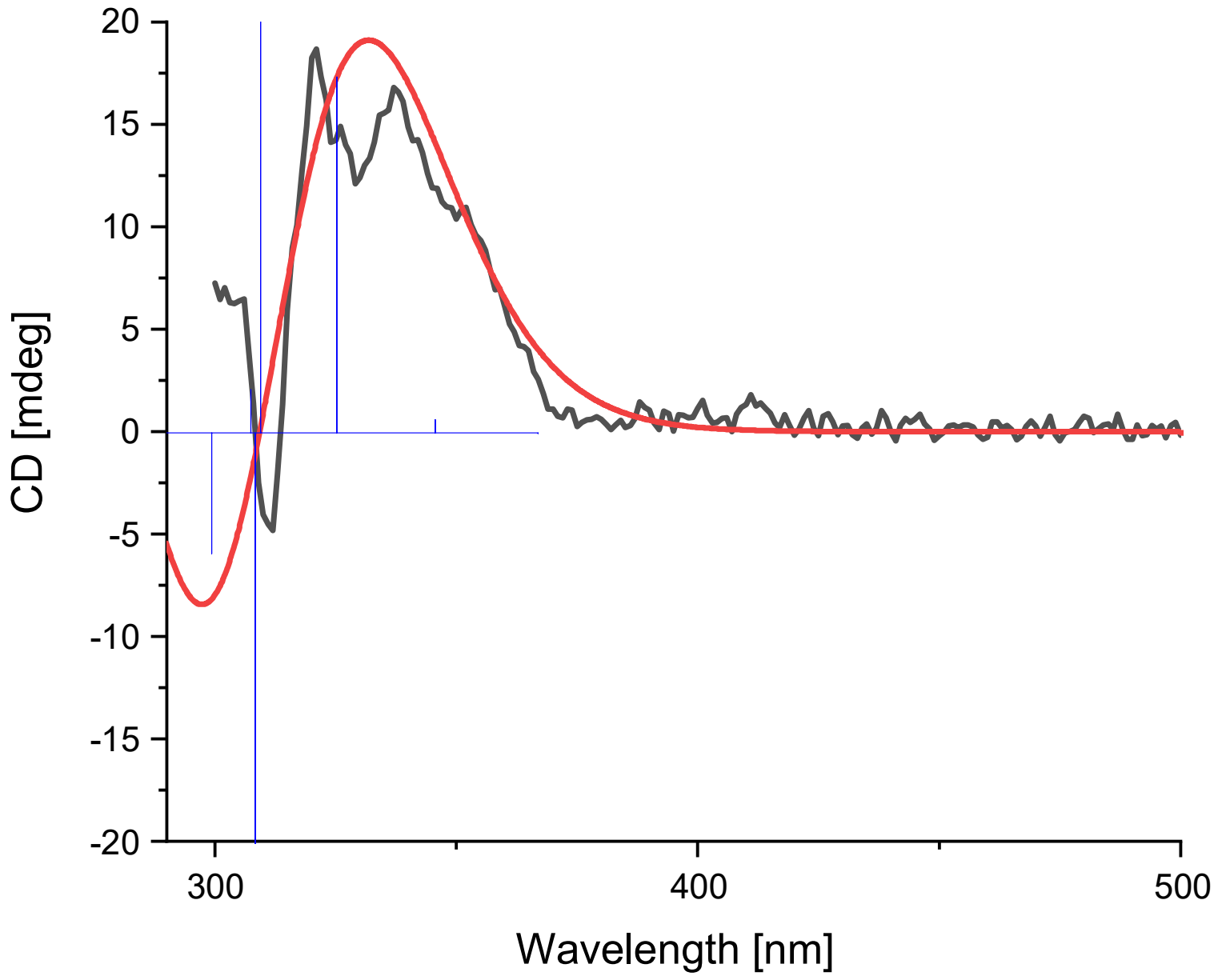

Figure S 43. Calculated CD spectrum of (R_a,R_a)-(P)-**1ap** (red), the calculated singlet transitions (blue) and the measured experimental spectrum of the third eluting peak of the HPLC (Figure S9).

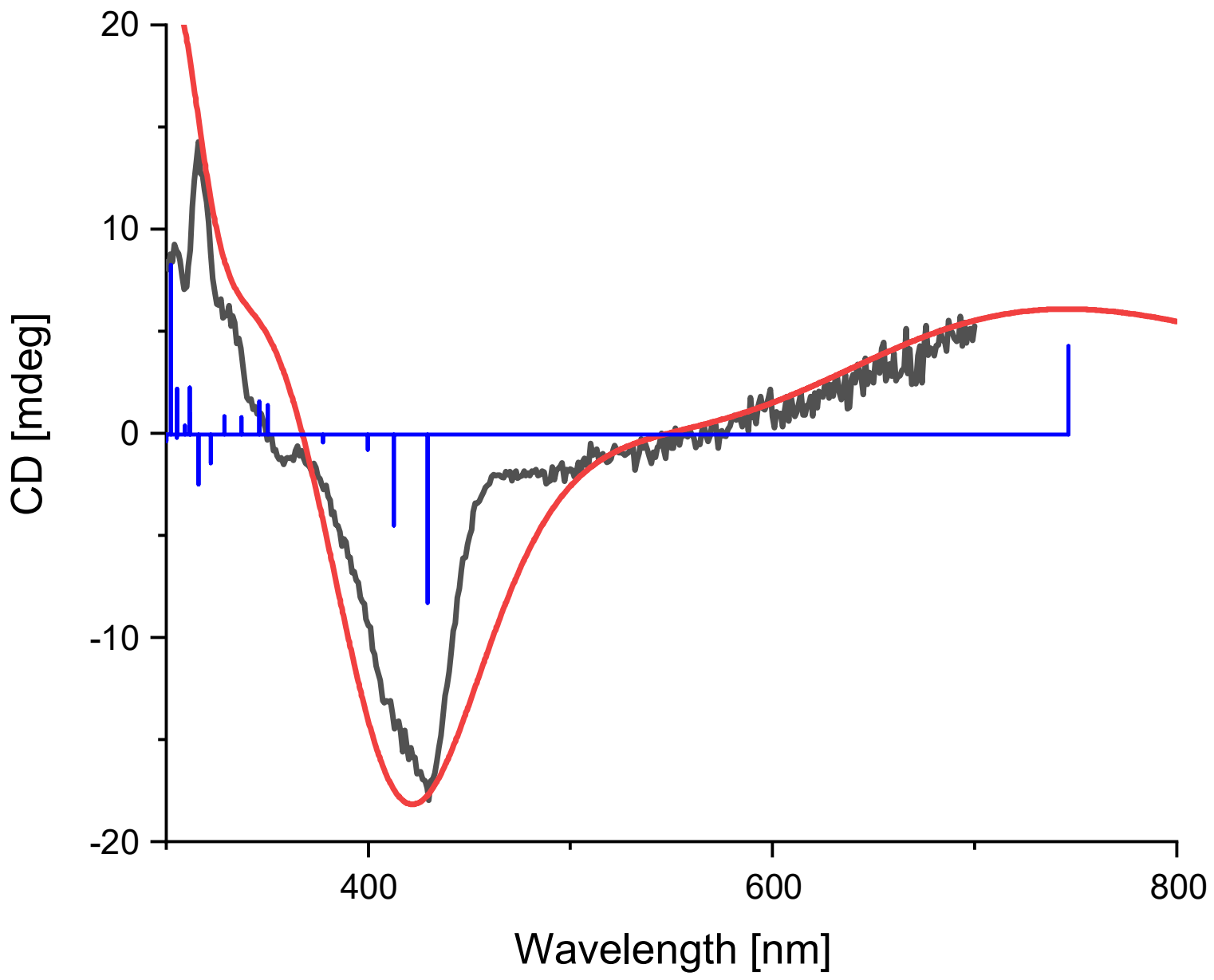

Figure S 44. Calculated CD spectrum of (S,S)-(P)-**1c** (red), the calculated singlet transitions (blue) and the measured experimental spectrum of the ring-closed species obtained by irradiating the third eluting peak of the HPLC at −80 °C(Figure S9).

References

(1) Neese, F.; Wennmohs, F.; Becker, U.; Riplinger, C. The ORCA Quantum Chemistry Program Package. *J. Chem. Phys.* **2020**, *152* (22), 224108.

(2) Li, R.; Fang, Q.; Chen, M.; Yamada, M.; Tsuji, Y.; Kugai, Y.; Li, W.; Kawai, T. Synthesis and Photochromic Properties of Diaryl [5]Helicene Derivatives. *Chem. – Eur. J.* **2023**, *29* (72), e202302693.

(3) Goretta, S.; Tasciotti, C.; Mathieu, S.; Smet, M.; Maes, W.; Chabre, Y. M.; Dehaen, W.; Giasson, R.; Raimundo, J.-M.; Henry, C. R.; Barth, C.; Gingras, M. Expeditive Syntheses of Functionalized Pentahelicenes and NC-AFM on Ag(001). *Org. Lett.* **2009**, *11* (17), 3846–3849.

(4) Wilm, L. F. B.; Das, M.; Janssen-Müller, D.; Mück-Lichtenfeld, C.; Glorius, F.; Dielmann, F. Photoswitchable Nitrogen Superbases: Using Light for Reversible Carbon Dioxide Capture. *Angew. Chem. Int. Ed.* **2022**, *61* (3), e202112344.

(5) Kuhn, H. J.; Braslavsky, S. E.; Schmidt, R. Chemical Actinometry. *Pure Appl. Chem.* **2004**, *76*, 2105–2146.

(6) Stranius, K.; Börjesson, K. Determining the Photoisomerization Quantum Yield of Photoswitchable Molecules in Solution and in the Solid State. *Sci. Rep.* **2017**, *7*, 41145.

(7) Hoops, S.; Sahle, S.; Gauges, R.; Lee, C.; Pahle, J.; Simus, N.; Singhal, M.; Xu, L.; Mendes, P. COPASI – a Complex Pathway Simulator. *Bioinformatics* **2006**, *22*, 3067–3074.

(8) Grimme, S.; Hansen, A.; Ehlert, S.; Mewes, J.-M. r2SCAN-3c: A "Swiss Army Knife" Composite Electronic-Structure Method. *J. Chem. Phys.* **2021**, *154* (6), 064103.

(9) Barone, V.; Cossi, M. Quantum Calculation of Molecular Energies and Energy Gradients in Solution by a Conductor Solvent Model. *J. Phys. Chem. A* **1998**, *102* (11), 1995–2001.

(10) Chai, J-D.; Head-Gordon, M. Systematic Optimization of Long-Range Corrected Hybrid Density Functionals. *J. Chem. Phys*, **2008**, *128*, 084106.

(11) Caldeweyher, E.; Bannwarth, C.; Grimme, S. Extension of the D3 Dispersion Coefficient Model. *J. Chem. Phys.* **2017**, *147*, 034112.

(12) Pracht, P.; Bohle, F.; Grimme, F. Automated exploration of the low-energy chemical space with fast quantum chemical methods. *Phys. Chem. Chem. Phys.* **2020**, *22*, 7169–7192.
